# Supplementary material for: Large-scale analysis of de novo mutations identifies risk genes for female infertility characterized by oocyte and early embryo defects
Source: Genome Biol. 2023 Apr 6;24:68. doi: 10.1186/s13059-023-02894-0 (PMC10080761; doi:10.1186/s13059-023-02894-0)
Supplement: Supplementary file 1 — Additional file 1. Supplementary Figures. [file 13059_2023_2894_MOESM1_ESM.pdf]

Large-scale analysis of de novo mutations identifies risk genes for female infertility characterized by oocyte and early embryo defects

<u>This file includes:</u>

Additional file 1: Supplementary Figures

Figure. S1. Quality control for total samples in this study.

Figure. S2. Single cell analysis of female fetal cells

Figure. S3. Dynamic expression of *de novo* mutant genes of probands in human female fetal germ cells, human folliculogenesis, human matured oocytes and early embryo development.

Figure. S4. Dynamic expression and pathways of *de novo* mutant genes among control group.

Figure. S5. Snapshot of IGV Viewer.

Figure. S6. Infertile parent-child trios with *de novo* mutations.

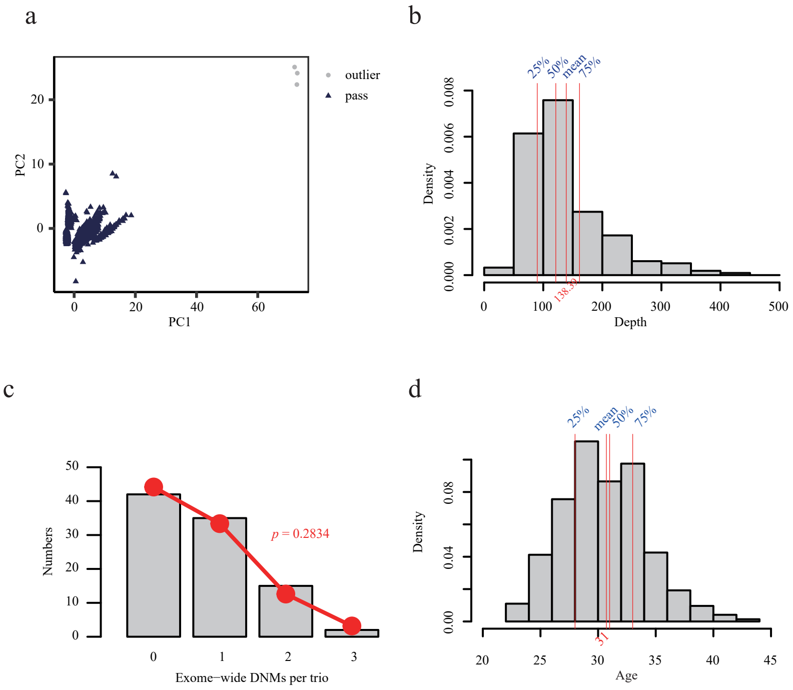

Fig. S1 . Quality control for total samples in this study.

(a) Principal component analysis (PCA) of total samples. Outliers were marked as grey circle and these were removed from downstream analysis.

(b) Distribution of sequence depth.

(c) Distribution of coding DNMs in siblings per trio.

(d) Distribution of age in all individuals.

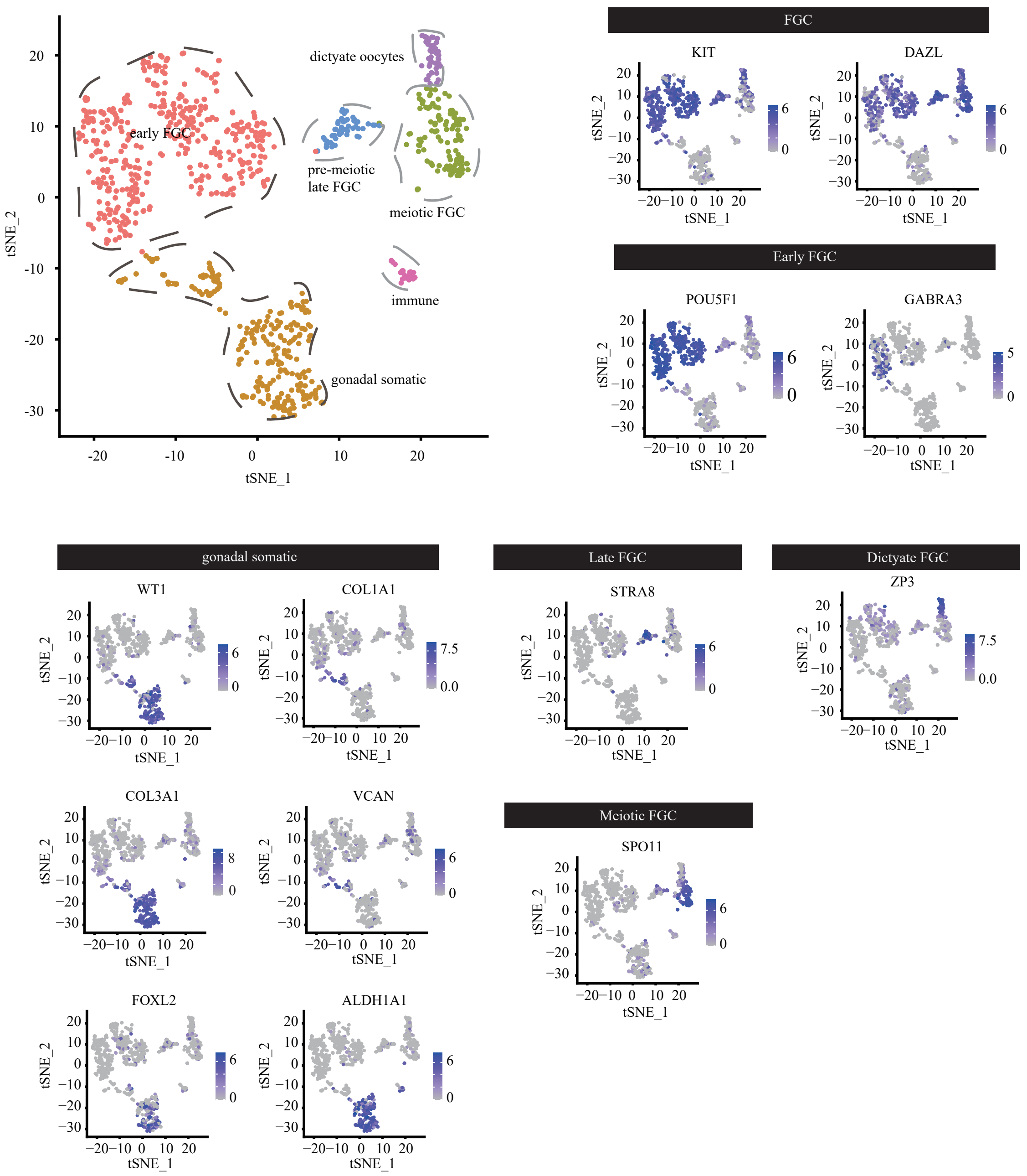

Fig. S2. Single cell analysis of female fetal cells

t-SNE analysis identified six clusters in female fetal cells (FGC), including immune, gonadal somatic, early FGC, pre-meiotic FGC, meiotic FGC and dictyate oocytes. Expression of markers for each major cell type are shown in cell cluster.

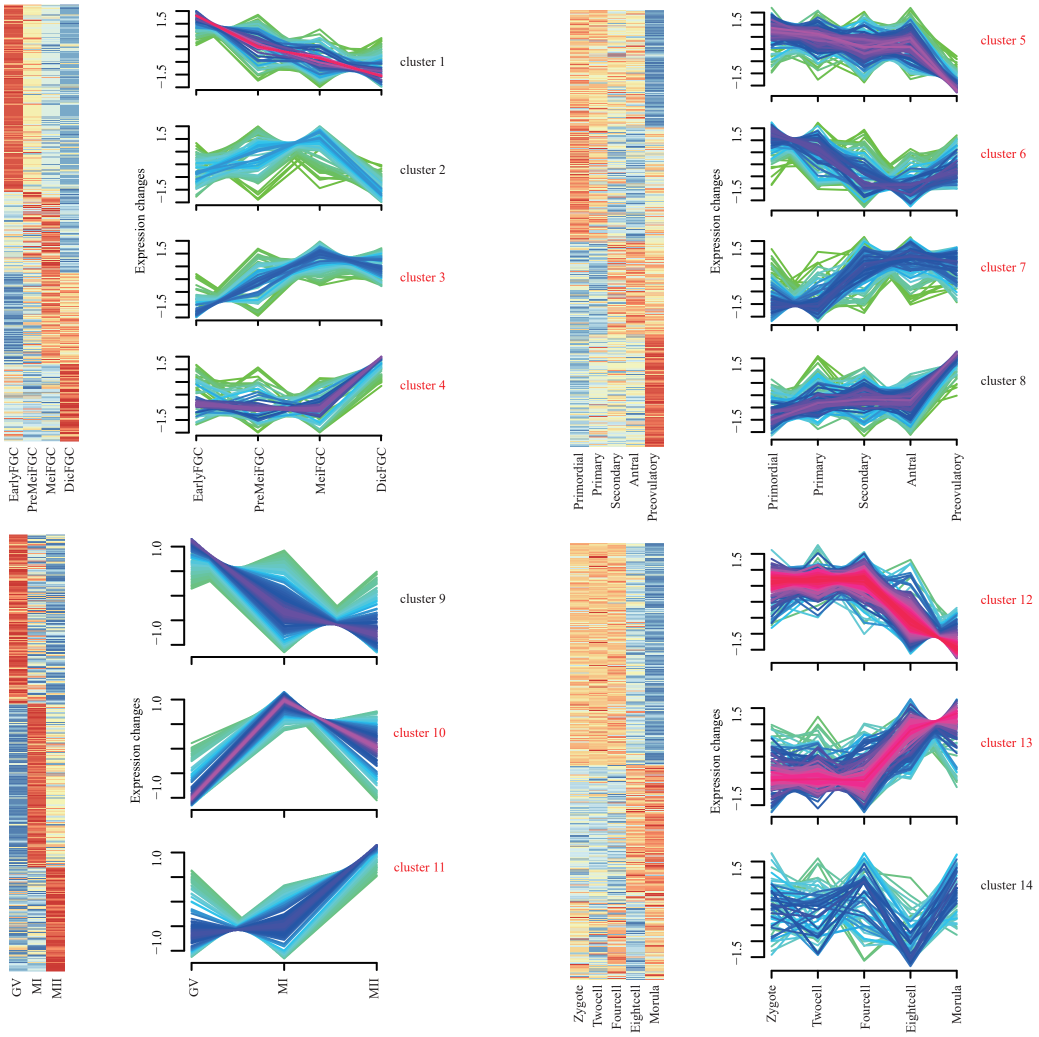

Cluster	Description	*p*	Gene
3	meiotic cell cycle	0.000185333	*ACTR3/CNTD1/DMRTC2/MEI1/TEX15/TTK/TUBGCP6*
3	spindle organization	0.001662723	*CLASP2/EML1/RMDN1/TTK/TUBGCP6*
3	female sex differentiation	0.002333831	*KMT2B/LHX8/MERTK/ZFPM2*
4	maintenance of cell number	0.000291956	*FZD7/JAG1/NOTCH1/PROX1/TP63*
4	urogenital system development	0.001114269	*JAG1/LGR4/NOTCH1/PCSK5/PROX1/TP63*
4	ectoderm development	0.002741358	*EDA2R/FZD7*
5	chromatin-mediated maintenance of transcription	0.002474848	*ZMIZ1/ZMIZ2*
5	homeostasis of number of cells	0.003723402	*CD74/JAK3/NKX2-3/NOTCH1/SRF/TCIRG1*
6	genitalia development	0.000149873	*BMP5/LGR4/ROR2/TP63*
6	genitalia morphogenesis	0.002824875	*ROR2/TP63*
6	mitochondrion distribution	0.003697403	*LRRK2/MAPT*
7	meiosis I cell cycle process	0.000230657	*CNTD1/DMRTC2/ESPL1/ING2/MEI1*
7	regulation of histone modification	0.000537202	*DMRTC2/GLYR1/ING2/KMT2B/TRIP12*
7	germ cell development	0.00768535	*DMRTC2/ING2/KMT2B/MEI1/TSSK1B*
7	sex differentiation	0.008272236	*ADCYAP1R1/DMRTC2/ING2/KMT2B/ROBO2*
10	in utero embryonic development	0.000281416	*ACVR1/DSC3/FURIN/MIB1/MYH6/NOTCH1/PLG/SKIL/TRIM28/ZFPM2/ZMIZ1*
10	positive regulation of cell morphogenesis involved in differentiation	0.001725174	*ARMCX5-GPRASP2/CUL7/MACF1/MAPT/PLXNA1/SKIL*
10	mitochondrion distribution	0.007185892	*LRRK2/MAPT*
10	reproductive structure development	0.009839197	*CUL7/KMT2B/LGR4/NOTCH1/PLG/PSAPL1/TP63/TRIM28/ZFPM2*
10	regulation of translational initiation	0.003810414	*EIF2AK1/EIF2AK4/FMR1/PPP1R15B*
11	meiotic chromosome segregation	0.000129629	*BUB1/MEI1/MLH3/TEX15/TTK*
12	female sex differentiation	0.000323751	*ADCYAP1R1/KMT2B/LHX8/MERTK/ROBO2/TP63/ZFPM2*
12	development of primary female sexual characteristics	0.005793356	*ADCYAP1R1/KMT2B/LHX8/ROBO2/ZFPM2*
12	positive regulation of cell morphogenesis involved in differentiation	0.001666915	*ARMCX5-GPRASP2/BAIAP2/EEF2K/MAPT/ROBO2/SRF/STK25*
13	positive regulation of Wnt signaling pathway	4.14E-06	*LGR4/LRRK2/MACF1/PSMA1/ROR2/RSPO1/USP47/WNK1/WNT10B*
13	positive regulation of cell morphogenesis involved in differentiation	0.000681659	*CUL7/LIMS1/MACF1/NEDD9/PLXNA2/SKIL*

Fig. S3. Dynamic expression of *de novo* mutant genes of probands in human female fetal germ cells, human folliculogenesis, human matured oocytes and early embryo development. Selected GO terms were listed below and genes related to female infertility were labeled as red color.

a

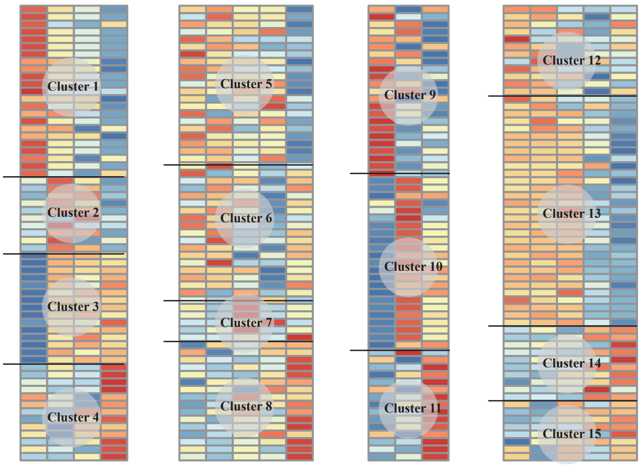

Cluster	Description	*p*	Gene
1	G protein-coupled receptor signaling pathway	0.000198379	*ADRA1A/CASR/MC5R/S1PR2*
2	positive regulation of mitochondrial calcium ion concentration	0.005236421	*ATP2A1*
3	nucleotide-excision repair, DNA damage recognition	0.000147847	*COPS8/CUL4B*
4	regulation of stem cell population maintenance	0.000283011	*SAV1/TP63*
5	positive regulation of cardiac muscle contraction	0.000128229	*ADRA1A/ATP2A1*
6	nucleotide-excision repair, DNA damage recognition	0.000147847	*COPS8/CUL4B*
7	regulation of endocytosis	0.001796058	*AAK1/ABCA7*
8	hippo signaling	9.42E-06	*SAV1/TEAD3/TJP1*
9	asymmetric stem cell division	7.54E-05	*ARHGEF2/TEAD3*
10	morphogenesis of an epithelial fold	0.000419299	*OVOL2/TP63*
11	vesicle docking	0.001035612	*EXOC6/STX17*
12	positive regulation of blood circulation	3.39E-05	*ADRA1A/ATP2A1/CASR*
14	nucleotide-excision repair, DNA damage recognition	0.000489852	*COPS8/CUL4B*
15	regulation of protein stability	0.003075043	*AAK1/APTX/GET4*

b

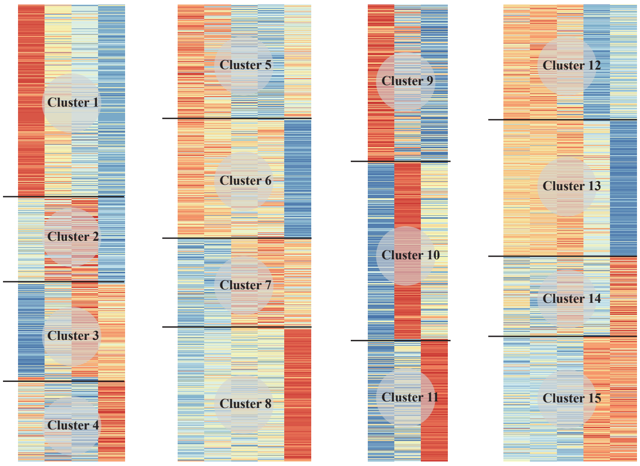

Cluster	Description	*p*	Gene
2	negative regulation of neuron differentiation	8.06E-05	*CERS2/EDNRB/EIF2AK4/EPHA7/NLGN3/ PAQR3/PLXNA3/SYNGAP1/THOC2*
4	membrane depolarization	1.04E-05	*ABL1/CACNA1E/P2RX7/PTPN3/SCN1A/SCN5A/SLC8A1*
7	semaphorin-plexin signaling pathway involved in axon guidance	2.91E-06	*PLXNA1/PLXNA3/PLXNA4/PLXND1*
11	retina development in camera-type eye	1.30E-05	*DCX/IGFN1/LRP5/LRP6/MYH10/PTPRM/TTLL5/TUB/VSX2/ZHX2*
12	cytoskeleton-dependent cytokinesis	0.000116868	*BIN3/MYH10/PRC1/SPAST/STAMBP/USP8/ZFYVE26*
13	microtubule-based movement	3.29E-05	*COPG1/COPG2/DNAH10/DYNC2H1/HTT/IFT57/KIF24/KIF6/ KLC2/NPHP4/RFX3/STARD9/SYNE2/TTLL9/TUB/WASF1*
15	selective autophagy	8.03E-05	*ATG2A/BAG3/BECN1/RB1CC1/SPTLC2/TSC2*

c

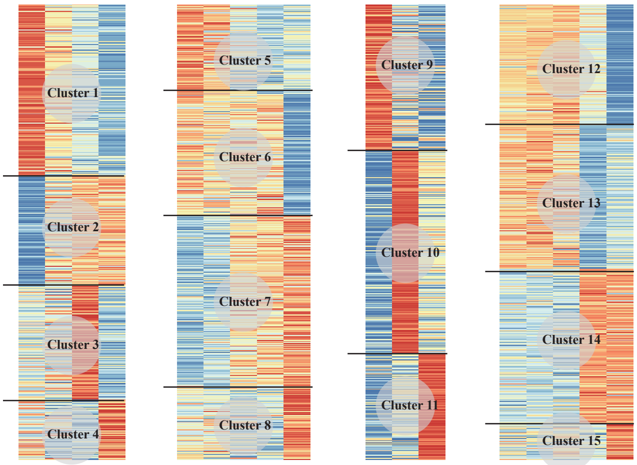

Cluster	Description	*p*	Gene
1	axonogenesis	2.86E-06	*CHL1/DSCAM/EPHB6/GLI2/ITGB1/L1CAM/LAMA1/ LAMB2/LMX1A/MARK2/MYPN/NFIB/NTNG1/PHOX2B/ PLXND1/PTPRZ1/SHANK3/SLITRK2/TNN/TUBB3/WNT5A*
1	synapse organization	7.80E-06	*DSCAM/GJA10/IGSF9B/L1CAM/LAMB2/LMX1A/LRRTM3/ MECP2/NLGN2/NTNG1/PCDHB13/PCDHB16/PLXND1/ SHANK3/SLC6A1/SLC8A3/SLITRK2/VCP/WNT5A*
4	membrane depolarization	3.84E-06	*ABL1/CACNA1A/CACNA1D/CACNA1E/PTPN3/SCN1A/SLC8A1*
4	cranial nerve development	3.03E-05	*ATP8B1/EPHB1/EPHB2/NAV2/PLXNA4*
5	synapse assembly	0.000770455	*ADNP/DSCAM/EIF4G1/EPHB2/GJA10/LRRTM3/MAP1B/ PCDHB16/SHANK2/SLITRK2/WNT5A*
8	regulation of sister chromatid cohesion	2.51E-05	*ATRX/CTNNB1/SMC5/TNKS*
10	axonogenesis	9.49E-07	*ABL1/ADNP/DSCAM/EPHA7/GLI2/L1CAM/LAMA1/ LAMA3/LMX1A/MACF1/NFIB/NTNG1/PHOX2B/PLXNA4/ PTEN/RELN/SHANK3/SLITRK2/SPAST/TBR1/WNT5A*
11	neuron projection organization	7.06E-07	*ATP1A3/DOCK10/EPHB1/EPHB2/GRIN2B/RELN/ SHANK2/SHANK3/ZMYND8*
12	synapse organization	3.93E-05	*ABL1/ADAM10/DISC1/GJA10/MUSK/MYH10/NLGN2/ NLGN3/PCDHB13/PLXND1/PPFIA1/SLC6A1/SYNGAP1/WNT5A*
13	axonogenesis	1.61E-05	*ACTB/ADNP/CHL1/COL25A1/EPHA7/ITGB1/L1CAM/LAMA1/ LAMA3/LMX1A/MACF1/MAP1B/MYPN/PHOX2B/PTEN/TBR1*

Fig. S4. Dynamic expression and pathways of *de novo* mutant genes among control group. (a) 92 siblings, (b) 1011 external unaffected individuals and (c) 1097 ASD females in female in female germ cell development.

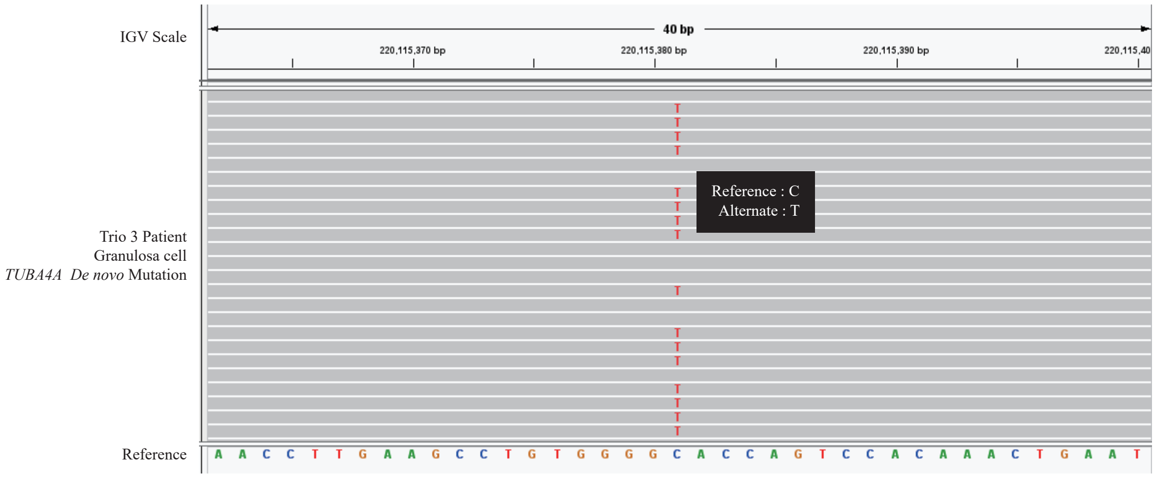

Fig. S5. Snapshot of IGV Viewer.
Granulosa cells of affected individuals in trio 3 were collected and processed with RNA-Seq pipeline. DNMs of *TUBA4A* were identified at RNA-level.

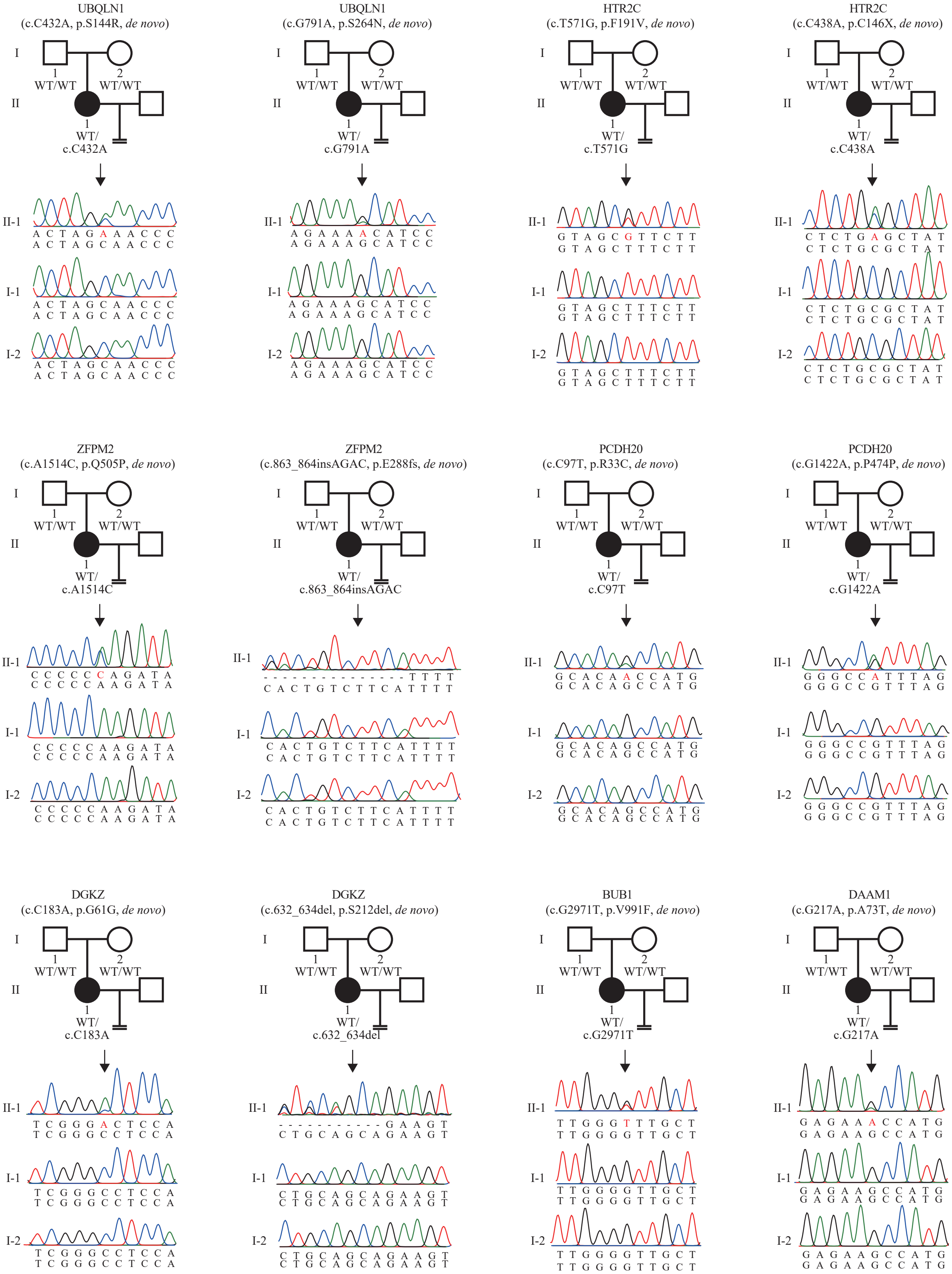
UBQLN1
(c.C432A, p.S144R, *de novo*)
I
1 WT/WT
2 WT/WT
II
1 WT/ c.C432A
II-1
A C T A G A A A C C C
A C T A G C A A C C C
I-1
A C T A G C A A C C C
A C T A G C A A C C C
I-2
A C T A G C A A C C C
A C T A G C A A C C C
UBQLN1
(c.G791A, p.S264N, *de novo*)
I
1 WT/WT
2 WT/WT
II
1 WT/ c.G791A
II-1
A G A A A A C A T C C
A G A A A G C A T C C
I-1
A G A A A G C A T C C
A G A A A G C A T C C
I-2
A G A A A G C A T C C
A G A A A G C A T C C
HTR2C
(c.T571G, p.F191V, *de novo*)
I
1 WT/WT
2 WT/WT
II
1 WT/ c.T571G
II-1
G T A G C G T T C T T
G T A G C T T T C T T
I-1
G T A G C T T T C T T
G T A G C T T T C T T
I-2
G T A G C T T T C T T
G T A G C T T T C T T
HTR2C
(c.C438A, p.C146X, *de novo*)
I
1 WT/WT
2 WT/WT
II
1 WT/ c.C438A
II-1
C T C T G A G C T A T
C T C T G C G C T A T
I-1
C T C T G C G C T A T
C T C T G C G C T A T
I-2
C T C T G C G C T A T
C T C T G C G C T A T
ZFPM2
(c.A1514C, p.Q505P, *de novo*)
I
1 WT/WT
2 WT/WT
II
1 WT/ c.A1514C
II-1
C C C C C C A G A T A
C C C C C A A G A T A
I-1
C C C C C A A G A T A
C C C C C A A G A T A
I-2
C C C C C A A G A T A
C C C C C A A G A T A
ZFPM2
(c.863_864insAGAC, p.E288fs, *de novo*)
I
1 WT/WT
2 WT/WT
II
1 WT/ c.863_864insAGAC
II-1
- - - - - - - - - - - T T T T
C A C T G T C T T C A T T T T
I-1
C A C T G T C T T C A T T T T
C A C T G T C T T C A T T T T
I-2
C A C T G T C T T C A T T T T
C A C T G T C T T C A T T T T
PCDH20
(c.C97T, p.R33C, *de novo*)
I
1 WT/WT
2 WT/WT
II
1 WT/ c.C97T
II-1
G C A C A A C C A T G
G C A C A G C C A T G
I-1
G C A C A G C C A T G
G C A C A G C C A T G
I-2
G C A C A G C C A T G
G C A C A G C C A T G
PCDH20
(c.G1422A, p.P474P, *de novo*)
I
1 WT/WT
2 WT/WT
II
1 WT/ c.G1422A
II-1
G G G C C A T T T A G
G G G C C G T T T A G
I-1
G G G C C G T T T A G
G G G C C G T T T A G
I-2
G G G C C G T T T A G
G G G C C G T T T A G
DGKZ
(c.C183A, p.G61G, *de novo*)
I
1 WT/WT
2 WT/WT
II
1 WT/ c.C183A
II-1
T C G G G A C T C C A
T C G G G C C T C C A
I-1
T C G G G C C T C C A
T C G G G C C T C C A
I-2
T C G G G C C T C C A
T C G G G C C T C C A
DGKZ
(c.632_634del, p.S212del, *de novo*)
I
1 WT/WT
2 WT/WT
II
1 WT/ c.632_634del
II-1
- - - - - - - - - G A A G T
C T G C A G C A G A A A G T
I-1
C T G C A G C A G A A A G T
C T G C A G C A G A A A G T
I-2
C T G C A G C A G A A A G T
C T G C A G C A G A A A G T
BUB1
(c.G2971T, p.V991F, *de novo*)
I
1 WT/WT
2 WT/WT
II
1 WT/ c.G2971T
II-1
T T G G G T T T G C T
T T G G G G T T G C T
I-1
T T G G G G T T G C T
T T G G G G T T G C T
I-2
T T G G G G T T G C T
T T G G G G T T G C T
DAAM1
(c.G217A, p.A73T, *de novo*)
I
1 WT/WT
2 WT/WT
II
1 WT/ c.G217A
II-1
G A G A A A C C A T G
G A G A A G C C A T G
I-1
G A G A A G C C A T G
G A G A A G C C A T G
I-2
G A G A A G C C A T G
G A G A A G C C A T G

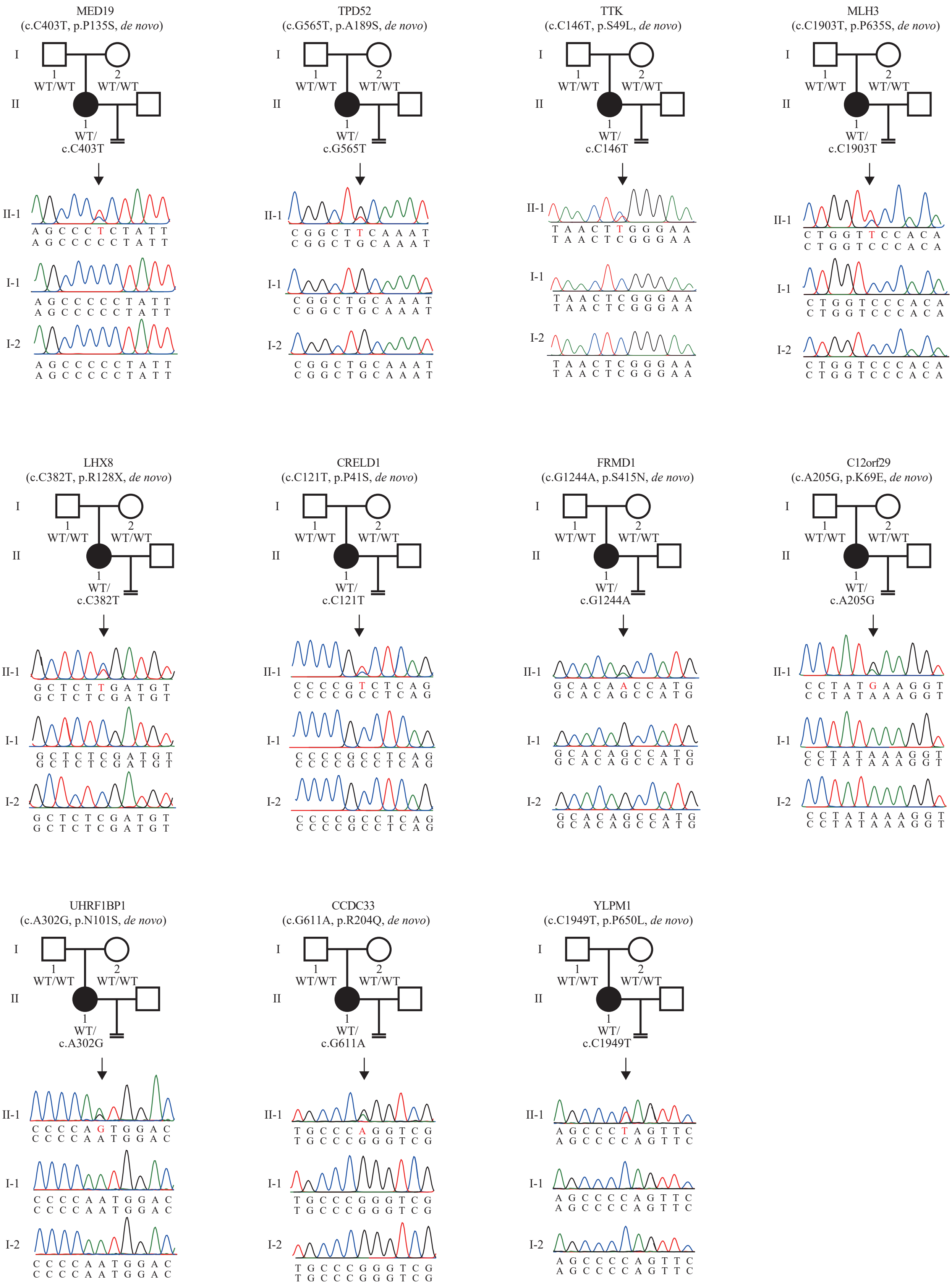

Fig. S6. Infertile parent-child trios with *de novo* mutations.
Squares denote male family members, circles denote female members, and solid symbols represent affected members. The '=' sign indicates infertility.
