# Supplementary material for: Large-scale analysis of de novo mutations identifies risk genes for female infertility characterized by oocyte and early embryo defects
Source: Genome Biol. 2023 Apr 6;24:68. doi: 10.1186/s13059-023-02894-0 (PMC10080761; doi:10.1186/s13059-023-02894-0)
Supplement: Supplementary file 2 — Additional file 2: Table S1. The clinical characteristics of individuals used in our cohort. Table S2. DNMs identified in this study. Table S3. GO terms of probands and unaffected individuals. Table S4. Selected genes and GO terms involved in female reproduction. Table S5. Genes associated with female reproduction defects. Table S6. Clinical IVF/ICSI information of patients with TUBA4A mutations. Table S7. TUBA4A pathogenic heterozygous mutations observed in the 12 families. Table S8. Primers used in this study. [file 13059_2023_2894_MOESM2_ESM.pdf]

Large-scale analysis of *de novo* mutations identifies risk genes for female infertility characterized by oocyte and early embryo defects

<u>This file includes:</u>

Additional file 2: Supplementary Tables

Table S1. The clinical characteristics of individuals used in our cohort.

Table S2. DNMs identified in this study.

Table S3. GO terms of probands and unaffected individuals.

Table S4. Selected genes and GO terms involved in female reproduction.

Table S5. Genes associated with female reproduction defects.

Table S6. Clinical IVF/ICSI information of patients with *TUBA4A* mutations.

Table S7. *TUBA4A* pathogenic heterozygous mutations observed in the 12 families.

Table S8. Primers used in this study.

Additional File Tables

Additional file 2 Table S1

Phenotypes	Type	Number of Samples
oocyte maturation arrest	trios	298
	sporadic	605
embryonic arrest	trios	175
	sporadic	550
control	unaffected siblings of cases with female infertility	92
	unaffected females without parent information	2813

Additional file 3 Table S2

PatientNumber	Chr	Start	End	Ref	Alt	Gene	Class	PP2 score	PP2 pred	pLI	Source
CPDID OMD0110	chr6	132873844	132873844	T	C	TAAR8	mis	0	B	NA	Proband
CPDID OMD0110	chr7	44805162	44805162	C	T	ZMIZ2	syn	NA	NA	NA	Proband
CPDID OMD0116	chr19	39597634	39597634	G	C	ACP7;PAPL	mis	1	D	NA	Proband
CPDID OMD0116	chr5	176915069	176915069	G	A	PDLIM7	syn	NA	NA	NA	Proband
CPDID OMD0124	chr17	40001903	40001903	G	A	KLHL10	mis	1	D	NA	Proband
CPDID OMD0141	chr11	2436476	2436476	G	A	TRPM5	mis		D	NA	Proband
CPDID OMD0158	chr1	89299095	89299095	G	A	PKN2	mis	0.013	B	NA	Proband
CPDID OMD0158	chrX	48891692	48891692	G	T	TFE3	mis	0.989	D	NA	Proband
CPDID OMD0214	chr17	7674673	7674673	G	A	DNAH2	mis	1	D	NA	Proband
CPDID OMD0214	chr8	135490794	135490794	G	A	ZFAT	syn	NA	NA	NA	Proband
CPDID OMD0278	chr19	56539273	56539273	G	A	NLRP5	syn	NA	NA	NA	Proband
CPDID OMD0278	chr3	126724992	126724992	C	T	PLXNA1	syn	NA	NA	NA	Proband
CPDID OMD0286	chr15	81201567	81201567	C	T	CEMIP;KIAA1199	mis	0.999	D	NA	Proband
CPDID OMD0286	chr6	6002702	6002702	C	A	NRN1	syn	NA	NA	NA	Proband
CPDID OMD0286	chr9	112219649	112219649	T	C	PTPN3	mis	0.12	B	NA	Proband
CPDID OMD0295	chr17	44067248	44067248	C	T	MAPT	mis	0.999	D	NA	Proband
CPDID OMD0295	chr1	46159082	46159082	C	T	TMEM69	syn	NA	NA	NA	Proband
CPDID OMD0301	chr3	49720087	49720087	C	T	APEH	mis	1	D	NA	Proband
CPDID OMD0308	chr2	30987139	30987139	G	A	CAPN13	syn	NA	NA	NA	Proband
CPDID OMD0328	chr4	103557137	103557137	T	A	MANBA	mis	0.999	D	NA	Proband
CPDID OMD0350	chr11	60640750	60640750	C	T	ZP1	mis	0.999	D	NA	Proband
CPDID OMD0353	chr10	125780762	125780762	-	GGGGC	CHST15	frameshift	NA	NA	0	Proband
CPDID OMD0353	chr6	42224469	42224469	C	T	TRERF1	syn	NA	NA	NA	Proband
CPDID OMD0353	chr8	106813824	106813824	A	C	ZFPM2	mis	0.996	D	NA	Proband
CPDID OMD0379	chr14	80327394	80327394	T	G	NRXN3	mis	0.61	P	NA	Proband
CPDID OMD0388	chr4	177084433	177084433	G	T	WDR17	mis	0.144	B	NA	Proband
CPDID OMD0395	chr16	71683515	71683515	T	C	PHLPP2	mis	0	B	NA	Proband
CPDID OMD0403	chr12	52282593	52282593	C	A	ANKRD33	mis	0.001	B	NA	Proband
CPDID OMD0411	chr16	4871569	4871569	C	T	GLYR1	syn	NA	NA	NA	Proband
CPDID OMD0417	chr19	41220453	41220453	G	T	COQ8B;ADCK4	mis	0.435	B	NA	Proband
CPDID OMD0423	chr1	75608825	75608825	C	T	LHX8	non	NA	NA	0.52	Proband
CPDID OMD0427	chr6	26225543	26225543	G	A	H3C6;HIST1H3E	mis	0	NA	NA	Proband
CPDID OMD0437	chr1	1225672	1225672	C	T	SCNN1D	non	NA	NA	0	Proband
CPDID OMD0439	chr15	43661195	43661196	CT	-	ZSCAN29	frameshift	NA	NA	0	Proband
CPDID OMD0440	chr12	27829371	27829371	C	T	PPFIBP1	mis	0.97	D	NA	Proband
CPDID OMD0444	chr3	16252711	16252711	C	T	GALNT15	mis	0.152	B	NA	Proband
CPDID OMD0444	chr12	48459377	48459378	AC	-	SENP1	splice	NA	NA	0.99	Proband
CPDID OMD0446	chr1	151144163	151144163	C	G	TMOD4	mis	0.879	P	NA	Proband
CPDID OMD0449	chr2	179721115	179721115	A	C	CCDC141	mis	1	D	NA	Proband
CPDID OMD0449	chrX	150912535	150912535	C	T	CNGA2	syn	NA	NA	NA	Proband
CPDID OMD0463	chr19	7987096	7987096	C	T	SNAPC2	syn	NA	NA	NA	Proband
CPDID OMD0464	chr10	50532672	50532672	C	A	C10orf71	mis	0	NA	NA	Proband
CPDID OMD0464	chr15	74564108	74564108	G	A	CCDC33	mis	1	D	NA	Proband
CPDID OMD0471	chr10	74100567	74100567	G	A	DNAJB12	mis	1	D	NA	Proband
CPDID OMD0471	chr17	41738591	41738591	C	T	MEOX1	syn	NA	NA	NA	Proband
CPDID OMD0473	chr6	26156915	26156915	C	T	H1-4;HIST1H1E	syn	NA	NA	NA	Proband
CPDID OMD0484	chr19	49148843	49148843	C	T	CA11	non	NA	NA	0	Proband
CPDID OMD0484	chr1	27226568	27226568	C	G	GPATCH3	syn	NA	NA	NA	Proband
CPDID OMD0484	chr2	33518253	33518253	G	A	LTBP1	mis	0.011	B	NA	Proband
CPDID OMD0484	chr6	131924291	131924294	GTGT	-	MED23	frameshift	NA	NA	0	Proband
CPDID OMD0504	chr9	86297882	86297882	G	T	UBQLN1	mis	0.756	P	NA	Proband
CPDID OMD0514	chr17	77709196	77709196	G	A	ENPP7	mis	0.991	D	NA	Proband
CPDID OMD0514	chr8	100732748	100732748	G	A	VPS13B	mis	0.999	D	NA	Proband
CPDID OMD0515	chr15	81633840	81633840	G	A	TMC3	mis	1	D	NA	Proband
CPDID OMD0518	chr14	92548749	92548749	C	T	ATXN3	mis	0.943	P	NA	Proband
CPDID OMD0522	chr5	127680140	127680140	C	T	FBN2	mis	0.99	D	NA	Proband
CPDID OMD0522	chr2	112779062	112779062	C	T	MERTK	syn	NA	NA	NA	Proband
CPDID OMD0526	chr17	64641554	64641554	G	T	PRKCA	non	NA	NA	0.37	Proband
CPDID OMD0527	chrX	114141267	114141267	T	G	HTR2C	mis	0	B	NA	Proband
CPDID OMD0527	chr15	63353945	63353945	T	G	TPM1	syn	NA	NA	NA	Proband
CPDID OMD0530	chr11	118769269	118769269	A	C	BCL9L	mis	0.036	B	NA	Proband
CPDID OMD0530	chr1	157740438	157740438	G	T	FCRL2	mis	0.999	D	NA	Proband
CPDID OMD0530	chr1	55158212	55158212	C	T	MROH7	mis	1	D	NA	Proband
CPDID OMD0531	chr18	77895999	77895999	G	T	ADNP2	mis	0.017	B	NA	Proband
CPDID OMD0536	chr5	40936470	40936470	A	G	C7	mis	1	D	NA	Proband
CPDID OMD0536	chr7	155255200	155255200	C	T	EN2	mis	1	D	NA	Proband
CPDID OMD0536	chr10	131934715	131934715	G	A	GLRX3	mis	0.005	B	NA	Proband
CPDID OMD0536	chr16	30965079	30965079	C	T	ORAI3	mis	1	D	NA	Proband
CPDID OMD0545	chr4	184426518	184426518	A	C	ING2	mis	0.971	D	NA	Proband
CPDID OMD0550	chr9	99145907	99145907	C	T	SLC35D2	mis		B	NA	Proband
CPDID OMD0550	chr11	63964825	63964825	A	G	STIP1	mis	0	B	NA	Proband
CPDID OMD0553	chr5	96508930	96508930	A	G	RIOK2	mis	0.851	P	NA	Proband
CPDID OMD0553	chr11	43427163	43427163	G	T	TTC17	non	NA	NA	1	Proband
CPDID OMD0555	chr11	102592135	102592135	C	T	MMP8	mis	0.001	B	NA	Proband
CPDID OMD0555	chr19	804613	804613	G	A	PTBP1	mis	0.026	B	NA	Proband
CPDID OMD0557	chr13	52673878	52673878	C	T	NEK5	splice	NA	NA	0	Proband
CPDID OMD0558	chr1	78958907	78958907	G	A	PTGFR	mis	0.919	P	NA	Proband
CPDID OMD0562	chrX	34148487	34148487	C	T	FAM47A	mis	0.001	B	NA	Proband
CPDID OMD0562	chr9	35811425	35811425	G	A	SPAG8	syn	NA	NA	NA	Proband
CPDID OMD0562	chr19	22256366	22256366	G	T	ZNF257	mis		B	NA	Proband
CPDID OMD0563	chr9	135917552	135917552	C	T	GTF3C5	mis	1	D	NA	Proband
CPDID OMD0563	chr12	21715852	21715852	C	A	GYS2	mis	0.955	P	NA	Proband
CPDID OMD0563	chr2	141130588	141130588	C	A	LRP1B	mis	0.055	B	NA	Proband
CPDID OMD0563	chr19	1775357	1775357	G	A	ONECUT3	syn	NA	NA	NA	Proband
CPDID OMD0563	chr8	144681288	144681288	C	G	TIGD5	syn	NA	NA	NA	Proband
CPDID OMD0564	chr4	5754579	5754579	C	T	EVC	mis	0.97	D	NA	Proband
CPDID OMD0564	chr7	295883	295883	C	G	FAM20C	mis	0.999	D	NA	Proband
CPDID OMD0565	chr2	31428131	31428131	C	T	CAPN14	syn	NA	NA	NA	Proband
CPDID OMD0565	chr3	9743482	9743482	C	T	MTMR14	mis	0.814	P	NA	Proband
CPDID OMD0565	chr16	21213597	21213597	C	G	ZP2	mis	1	D	NA	Proband
CPDID OMD0567	chr22	32439277	32439277	T	C	SLC5A1	syn	NA	NA	NA	Proband
CPDID OMD0567	chr2	230636286	230636286	T	C	TRIP12	syn	NA	NA	NA	Proband
CPDID OMD0569	chr10	126686593	126686593	G	A	CTBP2	mis		D	NA	Proband
CPDID OMD0569	chr8	642586	642586	G	A	ERICH1	non	NA	NA	0	Proband
CPDID OMD0569	chr22	42269805	42269805	C	T	SREBF2	syn	NA	NA	NA	Proband
CPDID OMD0569	chr2	120194539	120194539	G	A	TMEM37	syn	NA	NA	NA	Proband
CPDID OMD0574	chr15	90209022	90209022	C	T	PLIN1	mis	0.875	P	NA	Proband
CPDID OMD0577	chr2	152467320	152467320	G	C	NEB	mis	1	D	NA	Proband

CPDID	OMD0581	chr10	26856309	26856309	-	A	APBB1IP	frameshift	NA	NA	0.85	Proband
CPDID	OMD0581	chr11	27402478	27402478	T	A	LGR4	mis	0.959	D	NA	Proband
CPDID	OMD0581	chr8	106811075	106811075	-	AGAC	ZFPM2	frameshift	NA	NA	1	Proband
CPDID	OMD0589	chr1	11187750	11187750	C	T	MTOR	syn	NA	NA	NA	Proband
CPDID	OMD0589	chr3	18391077	18391077	G	T	SATB1	mis	0.801	P	NA	Proband
CPDID	OMD0590	chr19	18542663	18542663	C	T	SSBP4	non	NA	NA	0.37	Proband
CPDID	OMD0593	chr7	44152913	44152913	C	T	AEBP1	syn	NA	NA	NA	Proband
CPDID	OMD0594	chrX	114082654	114082654	C	A	HTR2C	non	NA	NA	0.04	Proband
CPDID	OMD0596	chr19	14153356	14153356	G	A	IL27RA	mis	0.052	B	NA	Proband
CPDID	OMD0599	chr4	7783327	7783327	A	-	AFAP1	frameshift	NA	NA	0	Proband
CPDID	OMD0599	chr5	147774458	147774458	A	G	FBXO38	mis	0.939	P	NA	Proband
CPDID	OMD0599	chr7	139529239	139529239	C	T	TBXAS1	mis	0.763	P	NA	Proband
CPDID	OMD0605	chr11	47074006	47074006	C	T	C11orf49	mis	1	D	NA	Proband
CPDID	OMD0611	chr12	6091119	6091119	A	T	VWF	mis		B	NA	Proband
CPDID	OMD0613	chr5	179143146	179143146	A	G	CANX	syn	NA	NA	NA	Proband
CPDID	OMD0613	chr20	55206315	55206315	G	A	TFAP2C	mis	0.186	B	NA	Proband
CPDID	OMD0614	chr2	164466298	164466298	C	T	FIGN	mis	0.952	P	NA	Proband
CPDID	OMD0616	chr1	11918380	11918380	C	A	NPPB	mis	0.008	B	NA	Proband
CPDID	OMD0622	chr1	1133085	1133085	C	A	TTLL10	mis	0.914	P	NA	Proband
CPDID	OMD0627	chr6	31525924	31525924	C	T	NFKBIL1	mis	0.013	B	NA	Proband
CPDID	OMD0633	chr2	171070957	171070957	A	G	MYO3B	syn	NA	NA	NA	Proband
CPDID	OMD0633	chr13	111995137	111995137	C	T	TEX29	mis	0.045	B	NA	Proband
CPDID	OMD0639	chr19	9225817	9225817	G	A	OR7G1	mis	0.004	B	NA	Proband
CPDID	OMD0639	chr11	14539252	14539252	G	A	PSMA1	mis	0.02	B	NA	Proband
CPDID	OMD0640	chr11	134128968	134128968	A	G	ACAD8	mis	0.963	D	NA	Proband
CPDID	OMD0640	chr22	39883516	39883516	C	T	MGAT3	mis	0.994	D	NA	Proband
CPDID	OMD0640	chr6	54208096	54208096	G	A	TINAG	mis		B	NA	Proband
CPDID	OMD0641	chr7	111368564	111368564	C	T	DOCK4	syn	NA	NA	NA	Proband
CPDID	OMD0644	chr4	10445789	10445789	C	T	ZNF518B	mis	0.011	B	NA	Proband
CPDID	OMD0646	chr1	231044520	231044520	A	C	TTC13	syn	NA	NA	NA	Proband
CPDID	OMD0651	chr11	65547217	65547217	C	T	AP5B1	syn	NA	NA	NA	Proband
CPDID	OMD0652	chr11	62985123	62985123	G	A	SLC22A25	syn	NA	NA	NA	Proband
CPDID	OMD0655	chr17	7129438	7129438	C	T	DVL2	mis	1	D	NA	Proband
CPDID	OMD0655	chr19	33698034	33698034	C	-	LRP3	frameshift	NA	NA	0	Proband
CPDID	OMD0660	chr9	128070136	128070136	A	C	GAPVD1	mis	0.493	P	NA	Proband
CPDID	OMD0660	chr17	71410790	71410790	C	G	SDK2	mis	0.998	D	NA	Proband
CPDID	OMD0661	chr2	241463327	241463327	G	A	ANKMY1	mis	1	D	NA	Proband
CPDID	OMD0661	chr11	6415540	6415540	G	A	SMPD1	syn	NA	NA	NA	Proband
CPDID	OMD0661	chr5	112770141	112770141	G	A	TSSK1B	syn	NA	NA	NA	Proband
CPDID	OMD0662	chr4	3205778	3205778	C	T	HTT	mis	0	B	NA	Proband
CPDID	OMD0662	chr1	40434150	40434150	G	A	MFSD2A	splice	NA	NA	0.01	Proband
CPDID	OMD0663	chr4	77250072	77250072	G	A	CCDC158	syn	NA	NA	NA	Proband
CPDID	OMD0665	chr3	126676263	126676263	C	T	CHCHD6	mis	1	D	NA	Proband
CPDID	OMD0674	chr1	112524592	112524592	G	A	KCND3	mis	0.997	D	NA	Proband
CPDID	OMD0676	chr19	36273314	36273314	C	T	ARHGAP33	syn	NA	NA	NA	Proband
CPDID	OMD0677	chr14	23828934	23828934	C	T	EFS	syn	NA	NA	NA	Proband
CPDID	OMD0678	chr3	56771307	56771307	C	T	ARHGEF3	mis	0.703	P	NA	Proband
CPDID	OMD0678	chr12	121465445	121465445	G	C	OASL	mis	1	D	NA	Proband
CPDID	OMD0680	chr9	21141067	21141067	C	T	IFNW1	mis	0.989	D	NA	Proband
CPDID	OMD0695	chr5	82837781	82837781	G	A	VCAN	mis	0.999	D	NA	Proband
CPDID	OMD0698	chr12	132633428	132633428	G	A	NOC4L	mis	0.854	P	NA	Proband
CPDID	OMD0710	chr16	66592157	66592157	T	C	CKLF;CKLF-CMTM1	mis	0.944	P	NA	Proband
CPDID	OMD0712	chr15	64275759	64275759	C	T	DAPK2	mis	0.972	D	NA	Proband
CPDID	OMD0712	chr16	863442	863442	C	T	PRR25	mis	0.989	D	NA	Proband
CPDID	OMD0718	chr7	31124415	31124415	C	T	ADCYAP1R1	mis	1	D	NA	Proband
CPDID	OMD0720	chr20	4705654	4705654	G	A	PRND	mis	0.288	B	NA	Proband
CPDID	OMD0720	chr10	75874123	75874123	G	A	VCL	mis	1	D	NA	Proband
CPDID	OMD0730	chr6	74325124	74325124	A	G	SLC17A5	mis	0.009	B	NA	Proband
CPDID	OMD0733	chr2	113343922	113343922	C	T	CHCHD5	mis	0.958	D	NA	Proband
CPDID	OMD0733	chr19	4513574	4513574	G	A	PLIN4	mis	0.723	P	NA	Proband
CPDID	OMD0745	chr12	7528094	7528094	G	A	CD163L1	syn	NA	NA	NA	Proband
CPDID	OMD0745	chr1	157490296	157490296	C	T	FCRL5	mis	0.002	B	NA	Proband
CPDID	OMD0745	chr1	153785753	153785753	T	A	GATAD2B	syn	NA	NA	NA	Proband
CPDID	OMD0747	chr1	225490906	225490906	C	A	DNAH14	mis		P	NA	Proband
CPDID	OMD0747	chr7	6078239	6078239	T	A	EIF2AK1	mis	0.955	P	NA	Proband
CPDID	OMD0747	chr1	156261283	156261283	T	C	TMEM79	mis	0.991	D	NA	Proband
CPDID	OMD0751	chr22	42099403	42099403	G	A	MEI1	syn	NA	NA	NA	Proband
CPDID	OMD0759	chr16	81253693	81253693	G	A	PKD1L2	mis	0.956	P	NA	Proband
CPDID	OMD0768	chr19	17439000	17439000	C	A	ANO8	non	NA	NA	0.96	Proband
CPDID	OMD0768	chr2	11725286	11725286	G	T	GREB1	non	NA	NA	0	Proband
CPDID	OMD0768	chr9	103204187	103204187	G	T	MSANTD3	non	NA	NA	0.02	Proband
CPDID	OMD0772	chr3	51459794	51459794	C	T	DCAF1;VPRBP	mis	0.927	P	NA	Proband
CPDID	OMD0774	chr4	76447047	76447047	A	G	THAP6	syn	NA	NA	NA	Proband
CPDID	OMD0774	chr11	117780571	117780571	G	A	TMPRSS13	syn	NA	NA	NA	Proband
CPDID	OMD0774	chr12	49360146	49360146	G	A	WNT10B	mis	0.022	B	NA	Proband
CPDID	OMD0775	chr3	132051086	132051086	A	-	ACP3;ACPP	frameshift	NA	NA	0	Proband
CPDID	OMD0775	chr3	189586471	189586471	G	A	TP63	syn	NA	NA	NA	Proband
CPDID	OMD0776	chr18	8813210	8813210	A	G	MTCL1;SOGA2	syn	NA	NA	NA	Proband
CPDID	OMD0779	chr2	219544737	219544737	T	C	STK36	mis	0.001	B	NA	Proband
CPDID	OMD078	chr4	148968124	148968124	C	T	ARHGAP10	mis	0.104	B	NA	Proband
CPDID	OMD078	chr10	50820284	50820284	C	G	SLC18A3	mis	0.382	B	NA	Proband
CPDID	OMD0780	chr11	71952270	71952270	C	T	PHOX2A	mis	1	D	NA	Proband
CPDID	OMD0781	chr15	79748810	79748810	G	A	MINAR1;KIAA1024	syn	NA	NA	NA	Proband
CPDID	OMD0784	chr3	128126977	128126977	C	T	EEFSEC	mis	0.947	P	NA	Proband
CPDID	OMD0786	chr2	206305274	206305274	C	G	PARD3B	syn	NA	NA	NA	Proband
CPDID	OMD0787	chr6	11193834	11193834	G	C	NEDD9	mis	0	B	NA	Proband
CPDID	OMD0787	chr20	34242963	34242963	G	A	RBM12	syn	NA	NA	NA	Proband
CPDID	OMD0793	chr1	21049277	21049277	C	T	SH2D5	mis	0.871	P	NA	Proband
CPDID	OMD0796	chr2	128707455	128707455	C	T	SAP130	mis	0.03	B	NA	Proband
CPDID	OMD0804	chrX	147744018	147744018	C	T	AFF2	mis	1	D	NA	Proband
CPDID	OMD0804	chr9	77613674	77613674	T	-	CARNMT1;C9orf41	frameshift	NA	NA	0	Proband
CPDID	OMD0806	chr1	43738870	43738870	G	A	TMEM125	syn	NA	NA	NA	Proband
CPDID	OMD0816	chr15	40268992	40268992	-	GACGAG(	EIF2AK4	nonframesh	NA	NA	NA	Proband
CPDID	OMD0816	chr13	61989195	61989195	G	A	PCDH20	mis	0.002	B	NA	Proband
CPDID	OMD0827	chr2	111397410	111397410	C	A	BUB1	mis	0.999	D	NA	Proband
CPDID	OMD0827	chr6	168457979	168457979	C	T	FRMD1	mis	0.965	D	NA	Proband
CPDID	OMD0829	chr17	79082296	79082296	C	T	BAIAP2	mis	1	D	NA	Proband
CPDID	OMD0839	chr8	37632955	37632955	A	G	PLPBP;PROSC	mis	0.425	B	NA	Proband
CPDID	OMD0840	chr3	123167042	123167042	C	T	ADCY5	syn	NA	NA	NA	Proband
CPDID	OMD0842	chr6	16327921	16327921	C	A	ATXN1	mis	1	D	NA	Proband
CPDID	OMD0842	chr10	116605175	116605175	C	T	FAM160B1	non	NA	NA	1	Proband

CPDID	OMD0842	chr3	196388366	196388366	A	G	NRROS	mis	0.001	B	NA	Proband
CPDID	OMD0842	chr8	30694612	30694612	C	T	TEX15	mis	0.994	D	NA	Proband
CPDID	OMD0854	chr19	59060520	59060520	C	T	TRIM28	syn	NA	NA	NA	Proband
CPDID	OMD0860	chrX	101970581	101970581	C	T	ARMCX5-GPRASP2;GPRASP2	mis	0.012	B	NA	Proband
CPDID	OMD0860	chr6	43194102	43194102	G	A	DNPH1	syn	NA	NA	NA	Proband
CPDID	OMD0868	chr22	38485540	38485540	A	G	BAIAP2L2	mis	0	B	NA	Proband
CPDID	OMD0868	chr17	80544065	80544065	G	A	FOXK2	mis		B	NA	Proband
CPDID	OMD0887	chr9	94538045	94538045	G	A	ROR2	syn	NA	NA	NA	Proband
CPDID	OMD0888	chr2	119915200	119915200	C	G	C1QL2	mis	1	D	NA	Proband
CPDID	OMD0891	chr3	19974914	19974914	G	A	EFHB	syn	NA	NA	NA	Proband
CPDID	OMD0899	chr3	38348794	38348794	T	C	SLC22A14	mis	0.271	B	NA	Proband
CPDID	OMD0927	chr3	9976243	9976243	C	T	CRELD1	mis	0.002	B	NA	Proband
CPDID	OMD0937	chr6	90581010	90581010	A	G	CASP8AP2	mis	0.972	D	NA	Proband
CPDID	OMD0938	chr12	98987790	98987790	A	C	SLC25A3	mis	0.994	D	NA	Proband
CPDID	OMD0938	chr5	1443028	1443028	G	A	SLC6A3	syn	NA	NA	NA	Proband
CPDID	OMD0939	chr9	140772427	140772427	C	T	CACNA1B	syn	NA	NA	NA	Proband
CPDID	OMD0945	chr12	88434039	88434039	A	G	C12orf29	mis	0.999	D	NA	Proband
CPDID	OMD0945	chr10	91177969	91177969	C	T	IFIT5	mis	0	B	NA	Proband
CPDID	OMD0945	chr22	50886729	50886729	G	A	SBF1	mis		NA	NA	Proband
CPDID	OMD0947	chr12	53685840	53685840	C	T	ESPL1	mis	0.998	D	NA	Proband
CPDID	OMD0947	chr13	61986810	61986810	C	T	PCDH20	syn	NA	NA	NA	Proband
CPDID	OMD0964	chr10	60573632	60573632	T	C	BICC1	syn	NA	NA	NA	Proband
CPDID	OMD0966	chr1	38082234	38082234	G	A	RSPO1	mis	1	D	NA	Proband
CPDID	OMD0968	chr8	87520786	87520786	G	A	RMDN1	mis	0.003	B	NA	Proband
CPDID	OMD0970	chr5	427780	427780	G	A	AHRR	mis	0.002	B	NA	Proband
CPDID	OMD0975	chr12	40646741	40646741	C	G	LRRK2	mis	0.972	D	NA	Proband
CPDID	OMD0987	chr14	100367268	100367268	A	G	EML1	syn	NA	NA	NA	Proband
CPDID	OMD0987	chr15	31212819	31212819	G	A	FAN1	mis	0.148	B	NA	Proband
CPDID	OMD0989	chr22	38823549	38823549	C	T	KCNJ4	mis	1	D	NA	Proband
CPDID	OMD0989	chr7	5334536	5334536	G	A	SLC29A4	mis	1	D	NA	Proband
CPDID	OMD0994	chr3	47046576	47046576	G	A	NBEAL2	mis	1	D	NA	Proband
CPDID	OMD0999	chr1	94463516	94463516	G	A	ABCA4	syn	NA	NA	NA	Proband
CPDID	OMD0999	chr3	185636155	185636155	G	A	TRA2B	mis	0.975	D	NA	Proband
CPDID	OMD1003	chr20	61943029	61943029	A	G	COL20A1	mis	0.478	P	NA	Proband
CPDID	OMD1003	chr3	124515297	124515297	T	C	ITGB5	mis	0.999	D	NA	Proband
CPDID	OMD1003	chr14	77718238	77718238	C	T	TMEM63C	syn	NA	NA	NA	Proband
CPDID	OMD1008	chrX	18195831	18195831	T	C	BEND2	syn	NA	NA	NA	Proband
CPDID	OMD1008	chr17	64873463	64873463	G	A	CACNG5	mis	0.832	P	NA	Proband
CPDID	OMD1008	chr19	44096550	44096550	C	T	IRGQ	syn	NA	NA	NA	Proband
CPDID	OMD1009	chr11	5537569	5537569	C	T	UBQLNL	mis	1	D	NA	Proband
CPDID	OMD1010	chr4	153574026	153574026	G	A	TMEM154	mis	0.002	B	NA	Proband
CPDID	OMD1015	chr6	30619052	30619052	A	G	C6orf136	syn	NA	NA	NA	Proband
CPDID	OMD1015	chr5	161116115	161116115	T	C	GABRA6	mis	0.999	D	NA	Proband
CPDID	OMD1016	chr9	117389188	117389188	G	A	TMEM268;C9orf91	mis	0.493	P	NA	Proband
CPDID	OMD1023	chr1	34068004	34068004	C	T	CSMD2	syn	NA	NA	NA	Proband
CPDID	OMD1023	chr3	170108134	170108134	T	C	SKIL	mis	0	B	NA	Proband
CPDID	OMD1027	chr14	75248695	75248695	C	T	YLPM1	mis	0.634	P	NA	Proband
CPDID	OMD1028	chrX	152801864	152801864	C	T	ATP2B3	syn	NA	NA	NA	Proband
CPDID	OMD1028	chr14	23868176	23868176	G	A	MYH6	mis	0.787	P	NA	Proband
CPDID	OMD1031	chr13	28674628	28674628	T	C	FLT3	mis	0	B	NA	Proband
CPDID	OMD1031	chr2	242437055	242437055	C	T	STK25	syn	NA	NA	NA	Proband
CPDID	OMD1039	chr2	50758479	50758479	G	A	NRXN1	mis	1	D	NA	Proband
CPDID	OMD1040	chr11	10064406	10064406	T	A	SBF2	syn	NA	NA	NA	Proband
CPDID	OMD1040	chr6	116600865	116600865	T	G	TSPYL1	syn	NA	NA	NA	Proband
CPDID	OMD1040	chr6	34791089	34791089	A	G	UHRF1BP1	mis	1	D	NA	Proband
CPDID	OMD1041	chr14	105836207	105836207	G	A	PACS2	syn	NA	NA	NA	Proband
CPDID	OMD1041	chr1	204379967	204379967	T	C	PPP1R15B	syn	NA	NA	NA	Proband
CPDID	OMD1043	chr19	17953157	17953157	C	T	JAK3	mis	0.052	B	NA	Proband
CPDID	OMD1043	chr11	61548705	61548705	C	T	MYRF	mis	0.001	B	NA	Proband
CPDID	OMD1043	chr9	139400190	139400190	C	T	NOTCH1	syn	NA	NA	NA	Proband
CPDID	OMD1048	chr3	49765584	49765584	G	A	IP6K1	syn	NA	NA	NA	Proband
CPDID	OMD1049	chr17	56565321	56565321	A	G	HSF5	syn	NA	NA	NA	Proband
CPDID	OMD1052	chr2	109237851	109237851	T	G	LIMS1	mis	0	B	NA	Proband
CPDID	OMD1053	chr5	55178946	55178946	A	G	IL31RA	mis	0.08	B	NA	Proband
CPDID	OMD1054	chr16	20981232	20981232	G	A	DNAH3	syn	NA	NA	NA	Proband
CPDID	OMD1054	chrX	86869031	86869031	C	T	KLHL4	mis	0.999	D	NA	Proband
CPDID	OMD1071	chr11	11941772	11941772	T	C	USP47	mis	1	D	NA	Proband
CPDID	OMD1071	chr16	85010701	85010701	C	G	ZDHHC7	syn	NA	NA	NA	Proband
CPDID	OMD1074	chr6	43144292	43144292	C	T	SRF	mis	0.816	P	NA	Proband
CPDID	OMD1080	chr14	105349121	105349121	G	A	CEP170B	mis	0.992	D	NA	Proband
CPDID	OMD1080	chr19	813127	813127	G	C	PLPPR3;LPPR3	mis	0.997	D	NA	Proband
CPDID	OMD1111	chr17	12897108	12897108	C	T	ELAC2	mis	0.999	D	NA	Proband
CPDID	OMD1113	chr3	138116308	138116308	C	T	MRAS	syn	NA	NA	NA	Proband
CPDID	OMD1117	chr16	3778413	3778413	T	C	CREBBP	mis	0.963	D	NA	Proband
CPDID	OMD1118	chr10	64943302	64943302	A	G	JMJD1C	mis	0.166	B	NA	Proband
CPDID	OMD1118	chrX	53457405	53457405	C	T	RIBC1	mis	0.147	B	NA	Proband
CPDID	OMD1118	chr9	136595251	136595251	C	T	SARDH	mis	0.913	P	NA	Proband
CPDID	OMD1128	chr14	96797746	96797746	A	T	ATG2B	mis	0.895	P	NA	Proband
CPDID	OMD1128	chr2	167163054	167163054	T	C	SCN9A	mis	0.006	B	NA	Proband
CPDID	OMD1128	chr12	978262	978262	C	T	WNK1	mis	0.002	B	NA	Proband
CPDID	OMD1134	chr2	165946959	165946959	C	T	SCN3A	mis	0.998	D	NA	Proband
CPDID	OMD1155	chr19	18499386	18499386	A	-	GDF15	frameshift	NA	NA	0	Proband
CPDID	OMD1188	chrX	153714195	153714195	G	A	UBL4A	mis	0.001	B	NA	Proband
CPDID	OMD1219	chr5	65083987	65083987	G	A	NLN	mis	0.998	D	NA	Proband
CPDID	OMD1235	chr2	114688945	114688945	A	G	ACTR3	mis	1	D	NA	Proband
CPDID	OMD1271	chr7	73455558	73455558	T	G	ELN	mis	0.999	D	NA	Proband
CPDID	OMD1271	chr15	34151838	34151838	A	C	RYR3	mis	0.999	D	NA	Proband
CPDID	OMD1271	chr7	47408379	47408379	G	C	TNS3	mis	0.068	B	NA	Proband
CPDID	OMD1274	chr2	3691072	3691072	G	A	COLEC11	mis	1	D	NA	Proband
CPDID	PT0011	chr20	3251172	3251172	G	A	C20orf194	mis	0.005	B	NA	Proband
CPDID	PT0011	chr7	139097326	139097326	G	T	FMC1-LUC7L2;LUC7L2	mis	0.972	D	NA	Proband
CPDID	PT0014	chr4	2648485	2648485	G	A	FAM193A	mis	0.995	D	NA	Proband
CPDID	PT0015	chr15	62276212	62276212	C	T	VPS13C	splice	NA	NA	0	Proband
CPDID	PT0019	chr20	10621830	10621830	G	A	JAG1	syn	NA	NA	NA	Proband
CPDID	PT0035	chr1	214178607	214178607	G	A	PROX1	mis	0.996	D	NA	Proband
CPDID	PT0035	chr1	209953898	209953898	C	T	TRAF3IP3	mis	0.01	B	NA	Proband
CPDID	PT0039	chr5	149428149	149428149	C	T	HMGXB3	syn	NA	NA	NA	Proband
CPDID	PT0046	chr22	38212683	38212683	G	A	GCAT	syn	NA	NA	NA	Proband
CPDID	PT0046	chr2	220115564	220115564	A	G	TUBA4A	mis	0.672	P	NA	Proband
CPDID	PT0048	chr4	145573870	145573870	G	A	HHIP	syn	NA	NA	NA	Proband
CPDID	PT0048	chr12	130963462	130963462	G	A	RIMBP2	non	NA	NA	0.68	Proband

CPDID	PT0098	chr17	10261117	10261117	C	T	MYH13	mis	0.056	B	NA	Proband
CPDID	PT0098	chr12	53452408	53452408	C	T	TNS2;TENC1	syn	NA	NA	NA	Proband
CPDID	PT0099	chr6	128411069	128411069	C	T	PTPRK	mis	1	D	NA	Proband
CPDID	PT0101	chr20	2776320	2776320	G	A	CPXM1	non	NA	NA	0	Proband
CPDID	PT0101	chr6	31938452	31938452	-	T	DXO	non	NA	NA	0	Proband
CPDID	PT0101	chr1	39853365	39853365	G	T	MACF1	non	NA	NA	1	Proband
CPDID	PT0101	chr14	20666175	20666175	-	A	OR11G2	frameshift	NA	NA	0	Proband
CPDID	PT0102	chr14	89181434	89181434	C	T	EML5	syn	NA	NA	NA	Proband
CPDID	PT0102	chr1	146680598	146680598	T	C	FMO5	mis	1	D	NA	Proband
CPDID	PT0106	chr5	141693815	141693815	T	C	SPRY4	mis	0	B	NA	Proband
CPDID	PT0106	chr8	80950410	80950410	C	A	TPD52	mis	1	D	NA	Proband
CPDID	PT0112	chr11	46388862	46388862	C	A	DGKZ	syn	NA	NA	NA	Proband
CPDID	PT0112	chr6	73108789	73108789	T	C	RIMS1	mis	1	D	NA	Proband
CPDID	PT0117	chr11	6649982	6649982	A	C	DCHS1	mis	0.907	P	NA	Proband
CPDID	PT0120	chr14	75514456	75514456	G	A	MLH3	mis	1	D	NA	Proband
CPDID	PT0120	chr3	126226599	126226599	G	A	UROC1	syn	NA	NA	NA	Proband
CPDID	PT0124	chr11	67811641	67811641	C	T	TCIRG1	mis	0.999	D	NA	Proband
CPDID	PT0129	chr16	22269956	22269956	G	A	EEF2K	mis	0.985	D	NA	Proband
CPDID	PT0129	chr20	62867991	62867991	C	T	MYT1	mis	0.997	D	NA	Proband
CPDID	PT0129	chr14	78198940	78198940	T	C	SNW1	mis	1	D	NA	Proband
CPDID	PT0134	chr6	161160127	161160127	C	T	PLG	syn	NA	NA	NA	Proband
CPDID	PT0134	chr3	111988783	111988783	A	C	SLC9C1	mis	0.998	D	NA	Proband
CPDID	PT0134	chr3	49847576	49847576	T	G	UBA7	syn	NA	NA	NA	Proband
CPDID	PT0135	chr2	193059173	193059173	G	A	TMEFF2	syn	NA	NA	NA	Proband
CPDID	PT0138	chr16	1604928	1604928	G	A	TMEM204	syn	NA	NA	NA	Proband
CPDID	PT0142	chr10	81070824	81070824	G	A	ZMIZ1	syn	NA	NA	NA	Proband
CPDID	PT0144	chr8	54147462	54147462	C	T	OPRK1	mis	1	D	NA	Proband
CPDID	PT0144	chr9	96422559	96422559	C	T	PHF2	mis	0.001	B	NA	Proband
CPDID	PT0144	chr2	86269096	86269096	A	G	POLR1A	mis	1	D	NA	Proband
CPDID	PT0144	chr14	57083998	57083998	C	T	TMEM260	mis	1	D	NA	Proband
CPDID	PT0144	chr12	133633977	133633977	C	T	ZNF84	non	NA	NA	NA	Proband
CPDID	PT0147	chr17	26961689	26961689	T	G	KIAA0100	mis	0.997	D	NA	Proband
CPDID	PT0149	chr9	140173656	140173656	T	C	TOR4A	mis	0.625	P	NA	Proband
CPDID	PT0150	chr1	159035963	159035963	T	C	AIM2	mis	0.224	B	NA	Proband
CPDID	PT0167	chr11	35457453	35457454	GG	-	PAMR1	non	NA	NA	0	Proband
CPDID	PT0171	chr15	91423194	91423194	C	T	FURIN	syn	NA	NA	NA	Proband
CPDID	PT0171	chr19	36223343	36223343	C	G	KMT2B	mis	0.015	B	NA	Proband
CPDID	PT0196	chr9	140008481	140008481	C	A	DPP7	splice	NA	NA	0	Proband
CPDID	PT0196	chr1	222801276	222801276	A	G	MIA3	syn	NA	NA	NA	Proband
CPDID	PT0196	chr6	158923489	158923489	A	G	TULP4	mis	0.998	D	NA	Proband
CPDID	PT0197	chr11	47198143	47198143	T	C	ARFGAP2	mis	0.581	P	NA	Proband
CPDID	PT0198	chrX	47835885	47835885	C	T	ZNF182	mis	0.991	D	NA	Proband
CPDID	PT0218	chr9	139351942	139351942	G	A	SEC16A	mis	0.435	B	NA	Proband
CPDID	PT0221	chr20	2413271	2413271	T	C	TGM6	syn	NA	NA	NA	Proband
CPDID	PT0222	chr15	75137539	75137539	C	T	SCAMP2	mis	0.803	P	NA	Proband
CPDID	PT0223	chr6	54735311	54735311	A	C	FAM83B	syn	NA	NA	NA	Proband
CPDID	PT0224	chr1	151824917	151824917	C	G	THEM5	mis	0.005	B	NA	Proband
CPDID	PT0226	chr16	10631869	10631869	C	T	EMP2	mis	0.969	D	NA	Proband
CPDID	PT0227	chr1	173743573	173743573	C	T	KLHL20	syn	NA	NA	NA	Proband
CPDID	PT0233	chr1	230979495	230979495	C	T	C1orf198	mis	0.876	P	NA	Proband
CPDID	PT0233	chr1	208270148	208270148	C	G	PLXNA2	syn	NA	NA	NA	Proband
CPDID	PT0237	chr4	62542634	62542634	G	A	ADGRL3;LPHN3	syn	NA	NA	NA	Proband
CPDID	PT0240	chr5	158630642	158630642	-	T	RNF145	frameshift	NA	NA	0.98	Proband
CPDID	PT0253	chr3	77657054	77657054	C	A	ROBO2	mis	0.996	D	NA	Proband
CPDID	PT0253	chr14	21827706	21827706	C	T	SUPT16H	syn	NA	NA	NA	Proband
CPDID	PT0266	chr3	56026205	56026205	G	A	ERC2	mis	0.001	B	NA	Proband
CPDID	PT0269	chr4	146025576	146025576	G	A	ABCE1	syn	NA	NA	NA	Proband
CPDID	PT0269	chr2	220115381	220115381	C	T	TUBA4A	mis	0.998	D	NA	Proband
CPDID	PT0273	chr3	141328816	141328816	A	G	RASA2	syn	NA	NA	NA	Proband
CPDID	PT0273	chr19	19625482	19625482	G	C	TSSK6	mis	0.983	D	NA	Proband
CPDID	PT0285	chrX	82763770	82763770	A	G	POU3F4	syn	NA	NA	NA	Proband
CPDID	PT0285	chr19	11034193	11034193	G	A	YIPF2	mis	0.049	B	NA	Proband
CPDID	PT0286	chr3	33617666	33617666	C	A	CLASP2	mis	0.999	D	NA	Proband
CPDID	PT0286	chr14	59757968	59757968	G	A	DAAM1	mis	0.997	D	NA	Proband
CPDID	PT0286	chr16	86612603	86612603	G	C	FOXL1	mis	1	D	NA	Proband
CPDID	PT0289	chr3	183041121	183041121	-	AA	MCF2L2	frameshift	NA	NA	0	Proband
CPDID	PT0289	chr11	57472516	57472516	G	A	MED19	mis	1	D	NA	Proband
CPDID	PT0289	chr11	117074936	117074936	C	T	TAGLN	mis	0.894	P	NA	Proband
CPDID	PT0293	chr6	55638846	55638846	C	T	BMP5	splice	NA	NA	0	Proband
CPDID	PT0293	chr1	228553810	228553810	C	G	OBSCN	mis	0.625	P	NA	Proband
CPDID	PT0293	chr9	140409897	140409897	G	C	PNPLA7	mis	0.012	B	NA	Proband
CPDID	PT0304	chr5	160039780	160039780	C	T	ATP10B	mis	1	D	NA	Proband
CPDID	PT0306	chr2	23916258	23916258	G	A	KLHL29	mis	0.371	B	NA	Proband
CPDID	PT0317	chr1	247978468	247978468	T	G	OR14A16	syn	NA	NA	NA	Proband
CPDID	PT0317	chr4	7435260	7435260	G	-	PSAPL1	frameshift	NA	NA	-1	Proband
CPDID	PT0319	chr17	40951213	40951213	C	T	CNTD1	mis		P	NA	Proband
CPDID	PT0319	chr14	39722386	39722386	C	G	MIA2	mis	0.004	B	NA	Proband
CPDID	PT0319	chr19	4525767	4525767	G	A	PLIN5	mis	1	D	NA	Proband
CPDID	PT0319	chr11	66103457	66103457	G	A	RIN1	mis		D	NA	Proband
CPDID	PT0329	chr2	158622640	158622640	C	T	ACVR1	mis	0.995	D	NA	Proband
CPDID	PT0330	chr11	624734	624734	T	C	CDHR5	splice	NA	NA	0	Proband
CPDID	PT0330	chr8	77912370	77912370	T	C	PEX2	splice	NA	NA	0	Proband
CPDID	PT0331	chr7	150883717	150883717	G	A	ASB10	syn	NA	NA	NA	Proband
CPDID	PT0332	chr14	24647343	24647343	G	A	REC8	mis	0.002	B	NA	Proband
CPDID	PT0332	chr3	44763646	44763646	A	G	ZNF502	mis	0.087	B	NA	Proband
CPDID	PT0335	chr10	97388190	97388190	C	T	ALDH18A1	mis	0.997	D	NA	Proband
CPDID	PT0335	chr2	231973376	231973376	C	T	HTR2B	mis	0.848	P	NA	Proband
CPDID	PT0335	chr22	50662653	50662653	A	G	TUBGCP6	syn	NA	NA	NA	Proband
CPDID	PT0339	chr6	30545854	30545854	-	A	ABCF1	frameshift	NA	NA	0	Proband
CPDID	PT0339	chr2	175677076	175677076	T	C	CHN1	mis	0.491	P	NA	Proband
CPDID	PT0339	chr5	140062728	140062728	C	T	HARS1;HARS	mis	1	D	NA	Proband
CPDID	PT0343	chr5	149786877	149786877	G	A	CD74	mis	1	D	NA	Proband
CPDID	PT0346	chr2	211540487	211540487	C	T	CPS1	syn	NA	NA	NA	Proband
CPDID	PT0346	chr1	18554503	18554503	G	A	IGSF21	mis	0.998	D	NA	Proband
CPDID	PT0346	chr3	39108268	39108268	A	G	WDR48	mis	0.947	P	NA	Proband
CPDID	PT0357	chr7	105254586	105254586	T	C	ATXN7L1	mis	0	B	NA	Proband
CPDID	PT0359	chr18	19371508	19371508	C	T	MIB1	mis	0.986	D	NA	Proband
CPDID	PT0360	chr12	56733256	56733256	A	G	IL23A	mis	0.001	B	NA	Proband
CPDID	PT0360	chr16	28332194	28332194	G	A	SBK1	syn	NA	NA	NA	Proband
CPDID	PT0365	chr14	45473494	45473494	G	A	TOGARAM1;FAM179B	mis	0.999	D	NA	Proband
CPDID	PT0366	chr15	42211571	42211571	C	T	EHD4	mis	1	D	NA	Proband

CPDID PT0367	chr12	124371710	124371710	C	T	DNAH10	non	NA	NA	0	Proband
CPDID PT0367	chr10	104486855	104486855	C	A	SFXN2	syn	NA	NA	NA	Proband
CPDID PT0372	chr19	57988644	57988644	C	G	ZNF772	splice	NA	NA	0	Proband
CPDID PT0380	chr19	45996429	45996429	A	G	RTN2	non	NA	NA	0.42	Proband
CPDID PT0387	chr7	5428768	5428768	G	A	TNRC18	syn	NA	NA	NA	Proband
CPDID PT0387	chr3	183518284	183518284	G	A	YEATS2	mis	0	B	NA	Proband
CPDID PT0391	chr5	178419001	178419001	G	A	GRM6	syn	NA	NA	NA	Proband
CPDID PT0401	chr5	138857097	138857097	C	T	STING1;TMEM173	mis	0.617	P	NA	Proband
CPDID PT0402	chr2	202900974	202900974	T	C	FZD7	mis	1	D	NA	Proband
CPDID PT0407	chrX	147011713	147011713	A	G	FMR1	mis	0.958	D	NA	Proband
CPDID PT0407	chr19	501701	501701	-	GAGCCTC	MADCAM1	frameshift	NA	NA	0.53	Proband
CPDID PT0408	chr6	26124108	26124108	G	A	H2BC4;HIST1H2BC	mis	0	B	NA	Proband
CPDID PT0408	chr3	150128771	150128771	C	T	TSC22D2	mis	0.923	P	NA	Proband
CPDID PT0410	chr11	46389548	46389550	GCA	-	DGKZ	splice	NA	NA	0.04	Proband
CPDID PT0410	chr10	101295278	101295278	G	-	NKX2-3	frameshift	NA	NA	0.95	Proband
CPDID PT0410	chr7	99631667	99631667	A	G	ZKSCAN1	syn	NA	NA	NA	Proband
CPDID PT0416	chr7	4841447	4841447	G	A	RADIL	syn	NA	NA	NA	Proband
CPDID PT0417	chr19	15636311	15636311	G	A	CYP4F22	mis		B	NA	Proband
CPDID PT0418	chr14	77493788	77493788	C	-	IRF2BPL	frameshift	NA	NA	0.84	Proband
CPDID PT0418	chr17	2186924	2186924	G	A	SMG6	mis	1	D	NA	Proband
CPDID PT0419	chr19	34838810	34838810	T	G	KIAA0355	non	NA	NA	0.97	Proband
CPDID PT0422	chr2	210574780	210574780	C	G	MAP2	syn	NA	NA	NA	Proband
CPDID PT0422	chr10	118220584	118220584	C	T	PNLIPRP3	syn	NA	NA	NA	Proband
CPDID PT0426	chr1	153651855	153651855	G	T	NPR1	mis	0	B	NA	Proband
CPDID PT0429	chr17	38975214	38975214	-	GCCGCCC	KRT10	nonframesh	NA	NA	NA	Proband
CPDID PT0429	chr5	1064359	1064359	G	A	SLC12A7	mis	0.904	P	NA	Proband
CPDID PT0433	chr9	86293435	86293435	C	T	UBQLN1	mis	1	D	NA	Proband
CPDID PT0435	chr6	139363979	139363979	C	T	ABRACL	mis	0.001	B	NA	Proband
CPDID PT0435	chr2	197208470	197208470	T	C	HECW2	mis	0.989	D	NA	Proband
CPDID PT0436	chr12	7045907	7045907	C	-	ATN1	frameshift	NA	NA	1	Proband
CPDID PT0436	chr6	126333988	126333988	T	C	TRMT11	mis	0.943	P	NA	Proband
CPDID PT0438	chr19	42354669	42354669	C	T	DMRTC2	mis	0.999	D	NA	Proband
CPDID PT0454	chr20	35434306	35434306	G	A	SOGA1	non	NA	NA	1	Proband
CPDID PT0472	chr2	237998540	237998540	A	G	COPS8	syn	NA	NA	NA	Proband
CPDID PT0473	chr10	93812043	93812043	C	T	CPEB3	mis	1	D	NA	Proband
CPDID PT0477	chr2	175346417	175346417	T	C	GPR155	mis	0.999	D	NA	Proband
CPDID PT0477	chr9	78547339	78547339	G	A	PCSK5	syn	NA	NA	NA	Proband
CPDID PT0477	chr19	6746076	6746076	G	A	TRIP10	mis		B	NA	Proband
CPDID PT0479	chr15	75105287	75105287	G	A	LMAN1L	mis	1	D	NA	Proband
CPDID PT0479	chr12	57499277	57499277	A	G	STAT6	syn	NA	NA	NA	Proband
CPDID PT0480	chr17	18874369	18874369	C	T	SLC5A10	syn	NA	NA	NA	Proband
CPDID PT0499	chr14	74539282	74539282	G	A	ALDH6A1	syn	NA	NA	NA	Proband
CPDID PT0500	chr8	1581178	1581178	C	T	DLGAP2	syn	NA	NA	NA	Proband
CPDID PT0509	chr3	46755964	46755964	C	A	PRSS50	syn	NA	NA	NA	Proband
CPDID PT0509	chr6	130476118	130476118	T	C	SAMD3	mis	0.483	P	NA	Proband
CPDID PT0597	chr13	26828861	26828861	-	C	CDK8	frameshift	NA	NA	0.41	Proband
CPDID PT0597	chr11	60274578	60274578	C	T	MS4A12	mis	0	B	NA	Proband
CPDID PT0696	chr10	74934547	74934547	A	G	FAM149B1	syn	NA	NA	NA	Proband
CPDID PT0696	chr10	91359119	91359119	-	T	PANK1	frameshift	NA	NA	0	Proband
CPDID PT0724	chr3	11060296	11060296	C	T	SLC6A1	mis	0.997	D	NA	Proband
CPDID PT0725	chr6	52657666	52657666	-	A	GSTA1	non	NA	NA	0	Proband
CPDID PT0727	chrX	65822603	65822603	G	A	EDA2R	mis	0.96	D	NA	Proband
CPDID PT0727	chr19	39055911	39055911	-	TGCGGCC	RYR1	nonframesh	NA	NA	NA	Proband
CPDID PT0731	chr11	66238785	66238785	C	T	PELI3	syn	NA	NA	NA	Proband
CPDID PT0745	chr16	1820675	1820675	C	G	NME3	mis	0	B	NA	Proband
CPDID PT0745	chr16	30744627	30744627	T	C	SRCAP	syn	NA	NA	NA	Proband
CPDID PT0745	chr4	147741285	147741285	T	A	TTC29	mis	1	D	NA	Proband
CPDID PT0798	chr11	1029108	1029108	C	T	MUC6	mis	1	D	NA	Proband
CPDID PT0848	chr6	50011452	50011452	G	A	DEFB112	non	NA	NA	0.02	Proband
CPDID PT0848	chr19	54844912	54844912	G	T	LILRA4	non	NA	NA	0	Proband
CPDID PT0848	chr8	2026918	2026918	C	T	MYOM2	non	NA	NA	0	Proband
CPDID PT0851	chr16	27481622	27481622	G	A	GTF3C1	mis	0.935	P	NA	Proband
CPDID PT0856	chr1	176145082	176145082	C	T	COP1;RFWD2	mis	0.95	P	NA	Proband
CPDID PT0856	chr5	118556201	118556201	A	C	DMXL1	mis	0.998	D	NA	Proband
CPDID PT0856	chr14	88454869	88454869	-	A	GALC	splice	NA	NA	0	Proband
CPDID PT0856	chr3	186507800	186507800	-	CTGAT	RFC4	frameshift	NA	NA	0	Proband
CPDID PT0889	chr6	80717532	80717532	C	T	TTK	mis	1	D	NA	Proband
CPDID PT0891	chr5	156378676	156378676	C	T	TIMD4	mis	0.707	P	NA	Proband
CPDID PT0893	chr2	133175276	133175276	C	T	GPR39	non	NA	NA	0	Proband
CPDID PT0895	chr17	2627802	2627802	G	A	CCDC92B	mis	0	NA	NA	Proband
CPDID PT0926	chr17	58980065	58980065	-	T	BCAS3	splice	NA	NA	0	Proband
CPDID PT0926	chr2	98306727	98306727	A	G	C2orf92	mis	0	NA	NA	Proband
CPDID PT0926	chr18	28612232	28612232	C	T	DSC3	mis	0.919	P	NA	Proband
CPDID PT0926	chr12	105509450	105509450	A	C	WASHC4;KIAA1033	mis	0.063	B	NA	Proband
CPDID PT0930	chr6	43020277	43020277	C	T	CUL7	mis	0.977	D	NA	Proband
CPDID PT0939	chr12	18837157	18837157	C	T	PLCZ1	mis	1	D	NA	Proband
CPDID PT0988	chr21	48068518	48068518	C	G	PRMT2	mis	0.999	D	NA	Proband
CPDID PT1042	chr11	2906217	2906217	G	A	CDKN1C	mis	0.002	B	NA	Proband
CPDID PT1309	chr1	82434923	82434923	C	T	ADGRL2;LPHN2	mis	1	D	NA	Proband
CPDID PT1309	chr2	220116433	220116433	C	T	TUBA4A	mis	0.005	B	NA	Proband
CSDID OMD0301	chr10	129535969	129535969	G	A	FOXI2	syn	NA	NA	NA	Sibling
CSDID OMD0463 6	chr6	31748894	31748894	G	A	VARS1	mis	1	D	NA	Sibling
CSDID_OMD0563	chr10	87628820	87628820	G	A	GRID1	mis	1	D	NA	Sibling
CSDID_OMD0563	chrX	153628171	153628171	A	G	RPL10	mis	0.908	P	NA	Sibling
CSDID OMD0572	chr11	34474699	34474699	G	A	CAT	mis	0.757	P	NA	Sibling
CSDID OMD0572	chr19	37646810	37646810	T	C	ZNF585A	syn	NA	NA	NA	Sibling
CSDID OMD0581 4	chr7	150327156	150327156	C	T	GIMAP6	syn	NA	NA	NA	Sibling
CSDID OMD0596	chr1	155932493	155932493	G	A	ARHGEF2	mis	0.999	D	NA	Sibling
CSDID OMD0596	chr2	37341874	37341874	C	G	EIF2AK2	mis	1	D	NA	Sibling
CSDID OMD0613 4	chr17	70645343	70645343	G	T	SLC39A11	syn	NA	NA	NA	Sibling
CSDID OMD0613 5	chr2	69771659	69771659	G	C	AAK1	mis	0.994	D	NA	Sibling
CSDID OMD0613 5	chr14	51111509	51111509	T	G	SAV1	syn	NA	NA	NA	Sibling
CSDID OMD0614 4	chr7	99802365	99802365	T	A	STAG3	mis	1	D	NA	Sibling
CSDID OMD0622	chr15	42168474	42168474	C	T	SPTBN5	syn	NA	NA	NA	Sibling
CSDID OMD0622	chr20	1286167	1286167	C	T	SNPH	syn	NA	NA	NA	Sibling
CSDID OMD0624	chr7	930664	930664	G	T	GET4	mis	0.412	B	NA	Sibling
CSDID OMD0624	chr12	100492264	100492264	T	C	UHRF1BP1L	mis	0.994	D	NA	Sibling
CSDID OMD0633	chr18	6896579	6896579	C	T	ARHGAP28	non	NA	NA	0	Sibling
CSDID OMD0633	chr20	47707520	47707520	C	G	CSE1L	mis	0.993	D	NA	Sibling

CSDID	OMD0640	chr22	20102537	20102537	C	G	TRMT2A	mis	1	D	NA	Sibling
CSDID	OMD0646	chr2	105885907	105885907	C	T	TGFBRAP1	mis	0.027	B	NA	Sibling
CSDID	OMD0654	chr16	28914377	28914377	G	A	ATP2A1	mis	0.243	B	NA	Sibling
CSDID	OMD0667	chr9	109686513	109686513	T	A	ZNF462	mis	0.99	D	NA	Sibling
CSDID	OMD0667	chr16	3026844	3026844	A	G	PKMYT1	mis	0.997	D	NA	Sibling
CSDID	OMD0676	chr13	42486240	42486240	C	T	VWA8	syn	NA	NA	NA	Sibling
CSDID	OMD0678	chr3	121980671	121980671	G	A	CASR	syn	NA	NA	NA	Sibling
CSDID	OMD0678	chr5	31317840	31317840	A	G	CDH6	mis	1	D	NA	Sibling
CSDID	OMD0678	chr14	51259531	51259531	C	T	NIN	mis	1	D	NA	Sibling
CSDID	OMD0705	chr18	756679	756679	G	C	YES1	mis	0	B	NA	Sibling
CSDID	OMD0710	chr9	140330261	140330261	G	A	ENTPD8	syn	NA	NA	NA	Sibling
CSDID	OMD0710	chr13	42875967	42875967	G	A	AKAP11	mis	0.01	B	NA	Sibling
CSDID	OMD0714	chr9	130551814	130551814	G	A	CDK9	mis	0.893	P	NA	Sibling
CSDID	OMD0730	chr2	103317688	103317688	A	C	SLC9A2	syn	NA	NA	NA	Sibling
CSDID	OMD0730	chr3	49168509	49168509	C	T	LAMB2	syn	NA	NA	NA	Sibling
CSDID	OMD0730	chr8	8185795	8185795	G	C	PRAG1;SGK223	mis	0.905	P	NA	Sibling
CSDID	OMD0766	chr19	1054045	1054045	C	T	ABCA7	syn	NA	NA	NA	Sibling
CSDID	OMD0768	chr12	133803604	133803604	C	T	ANHX	syn	NA	NA	NA	Sibling
CSDID	OMD0775	chr3	189586471	189586471	G	A	TP63	syn	NA	NA	NA	Sibling
CSDID	OMD0775	chr3	132051086	132051086	A	-	ACP3;ACPP	frameshift	NA	NA	0	Sibling
CSDID	OMD0804	chr19	10334871	10334871	G	C	S1PR2	mis	1	D	NA	Sibling
CSDID	OMD0826	chr1	228559536	228559537	AG	-	OBSCN	frameshift	NA	NA	0	Sibling
CSDID	OMD0829 5	chr6	56425125	56425125	C	T	DST	mis	0.999	D	NA	Sibling
CSDID	OMD0839 4	chr7	158540904	158540904	C	T	ESYT2	mis	0.999	D	NA	Sibling
CSDID	OMD0840	chr19	49341337	49341337	G	A	PLEKHA4	mis	0.994	D	NA	Sibling
CSDID	OMD0840	chr20	47265960	47265960	G	T	PREX1	mis	0.002	B	NA	Sibling
CSDID	OMD0850	chr12	3659224	3659224	C	T	PRMT8	syn	NA	NA	NA	Sibling
CSDID	OMD0970	chr20	18037381	18037381	T	G	OVOL2	mis	0	B	NA	Sibling
CSDID	OMD1031	chr19	6433507	6433507	C	T	SLC25A41	syn	NA	NA	NA	Sibling
CSDID	OMD1054 4	chr10	94669186	94669186	A	G	EXOC6	mis	1	D	NA	Sibling
CSDID	OMD1074	chr12	110340896	110340896	G	A	TCHP	mis	0.113	B	NA	Sibling
CSDID	PT0273	chr14	94849317	94849317	T	A	SERPINA1	syn	NA	NA	NA	Sibling
CSDID	PT0283 4	chr8	26722391	26722391	G	T	ADRA1A	syn	NA	NA	NA	Sibling
CSDID	PT0300	chr18	13826231	13826231	C	A	MC5R	mis	0.793	P	NA	Sibling
CSDID	PT0319	chr4	22389342	22389342	C	T	ADGRA3	mis	1	D	NA	Sibling
CSDID	PT0337	chr6	31721169	31721169	A	G	MSH5	mis	0.001	B	NA	Sibling
CSDID	PT0372	chr16	56899394	56899394	C	T	SLC12A3	mis	1	D	NA	Sibling
CSDID	PT0380	chr9	32987618	32987618	G	A	APTX	mis	0.001	B	NA	Sibling
CSDID	PT0407	chr3	43389308	43389308	A	G	SNRK	syn	NA	NA	NA	Sibling
CSDID	PT0407	chr9	102722296	102722296	G	A	STX17	syn	NA	NA	NA	Sibling
CSDID	PT0408	chr9	140008800	140008800	G	A	DPP7	syn	NA	NA	NA	Sibling
CSDID	PT0408	chrX	119668335	119668335	C	T	CUL4B	splice	NA	NA	1	Sibling
CSDID	PT0418	chr5	130815228	130815228	T	C	RAPGEF6	mis	0.938	P	NA	Sibling
CSDID	PT0418	chr6	35454438	35454438	A	G	TEAD3	non	0.611	P	0.99	Sibling
CSDID	PT0429 5	chr15	29997752	29997752	G	T	TJP1	mis	1	D	NA	Sibling
CSDID	PT0431 5	chr7	48684286	48684286	T	C	ABCA13	mis	0.604	P	NA	Sibling
CSDID	PT0435	chr20	40120435	40120435	G	A	CHD6	non	NA	NA	1	Sibling
CSDID	PT0472 4	chr2	237998540	237998540	A	G	COPS8	syn	NA	NA	NA	Sibling
CSDID	PT0472 4	chr19	10792668	10792670	GAG	-	ILF3	frameshift	NA	NA	1	Sibling
CSDID	PT0472 5	chr19	41774232	41774232	C	A	HNRNPUL1	mis	0.223	B	NA	Sibling

CPDID: Case Proband Denovo ID; CSDID: Case Sibling Denovo ID; NA: not available

OMD: Oocyte_maturation_arrest; PT: Embryo_arrest

syn: synonymous, mis: missense, non: nonsense(stop codon gains and losses), splice: canonical splice site , frameshift: frameshift

pLI: Probability of being loss-of-function intolerant

pLI were obtained from UCSC Table Browser. In general, pLI greater than 0.9 as an extremely intolerant set of transcripts.

Additional file 2 Table S3

Cluster	ID	Description	pvalue	geneID	Source
1	GO:0003272	endocardial cushion formation	7.02E-05	ACVR1/BMP5/DCHS1/ROBO2	ProbandFGC
1	GO:0030225	macrophage differentiation	7.86E-05	EIF2AK1/IL31RA/NKX2-3/NRROS/ROR2	ProbandFGC
1	GO:0019934	cGMP-mediated signaling	0.000173315	HTR2B/HTR2C/NPPB/NPR1	ProbandFGC
1	GO:0003007	heart morphogenesis	0.000175169	ACVR1/BMP5/DCHS1/DVL2/HTR2B/MYH6/ROBO2/SRF/TPM1/ZMIZ1	ProbandFGC
1	GO:0003203	endocardial cushion morphogenesis	0.000319186	ACVR1/BMP5/DCHS1/ROBO2	ProbandFGC
2	GO:0002286	T cell activation involved in immune response	4.03E-06	APBB1IP/CD74/EIF2AK4/IL23A/JAK3/MTOR	ProbandFGC
2	GO:0048872	homeostasis of number of cells	5.93E-05	CD74/FLT3/JAK3/KLHL10/SENP1/SKIL/WDR48	ProbandFGC
2	GO:0002285	lymphocyte activation involved in immune response	8.59E-05	APBB1IP/CD74/EIF2AK4/IL23A/JAK3/MTOR	ProbandFGC
2	GO:0001776	leukocyte homeostasis	0.000344718	CD74/FLT3/JAK3/SKIL	ProbandFGC
3	GO:0060998	regulation of dendritic spine development	3.18E-05	ARMCX5-GPRASP2/BAIAP2/CPEB3/FMR1/LRRK2	ProbandFGC
3	GO:0060999	positive regulation of dendritic spine development	5.20E-05	ARMCX5-GPRASP2/BAIAP2/CPEB3/FMR1	ProbandFGC
3	GO:0098815	modulation of excitatory postsynaptic potential	5.69E-05	BAIAP2/LRRK2/NRXN1/RIMS1	ProbandFGC
3	GO:0016570	histone modification	6.42E-05	CREBBP/DMRTC2/FMR1/JMJD1C/KMT2B/LRRK2/SATB1/SRCAP/TRERF1/TRIP12	ProbandFGC
3	GO:0016569	covalent chromatin modification	8.06E-05	CREBBP/DMRTC2/FMR1/JMJD1C/KMT2B/LRRK2/SATB1/SRCAP/TRERF1/TRIP12	ProbandFGC
3	GO:0060996	dendritic spine development	0.000114903	ARMCX5-GPRASP2/BAIAP2/CPEB3/FMR1/LRRK2	ProbandFGC
3	GO:0140013	meiotic nuclear division	0.000179647	ACTR3/CNTD1/DMRTC2/MEI1/TEX15/TTK	ProbandFGC
3	GO:0051321	meiotic cell cycle	0.000185333	ACTR3/CNTD1/DMRTC2/MEI1/TEX15/TTK/TUBGCP6	ProbandFGC
3	GO:1903046	meiotic cell cycle process	0.000286596	ACTR3/CNTD1/DMRTC2/MEI1/TEX15/TTK	ProbandFGC
3	GO:0048285	organelle fission	0.000375778	ACTR3/CLASP2/CNTD1/DMRTC2/LRRK2/MEI1/RMDN1/TEX15/TTK	ProbandFGC
3	GO:0010976	positive regulation of neuron projection development	0.000413881	ARMCX5-GPRASP2/BAIAP2/CPEB3/FBXO38/FMR1/RIMS1/STK25	ProbandFGC
3	GO:1900006	positive regulation of dendrite development	0.000417292	ARMCX5-GPRASP2/BAIAP2/CPEB3/FMR1	ProbandFGC
3	GO:0031346	positive regulation of cell projection organization	0.000505137	ACTR3/ARMCX5-GPRASP2/BAIAP2/CPEB3/FBXO38/FMR1/RIMS1/STK25	ProbandFGC
3	GO:0031644	regulation of nervous system process	0.000644751	BAIAP2/FMR1/LRRK2/NRXN1/RIMS1	ProbandFGC
3	GO:0031056	regulation of histone modification	0.000664715	DMRTC2/FMR1/KMT2B/LRRK2/TRIP12	ProbandFGC
3	GO:0050773	regulation of dendrite development	0.000794392	ARMCX5-GPRASP2/BAIAP2/CPEB3/FMR1/LRRK2	ProbandFGC
3	GO:0000280	nuclear division	0.00086669	ACTR3/CLASP2/CNTD1/DMRTC2/MEI1/RMDN1/TEX15/TTK	ProbandFGC
3	GO:0045132	meiotic chromosome segregation	0.000940572	ACTR3/MEI1/TEX15/TTK	ProbandFGC
3	GO:0000045	autophagosome assembly	0.001058862	ATG2B/LRRK2/PACS2/UBQLN1	ProbandFGC
3	GO:1905037	autophagosome organization	0.001187234	ATG2B/LRRK2/PACS2/UBQLN1	ProbandFGC
3	GO:0060079	excitatory postsynaptic potential	0.001374858	BAIAP2/LRRK2/NRXN1/RIMS1	ProbandFGC
3	GO:0099175	regulation of postsynapse organization	0.001582272	ARMCX5-GPRASP2/BAIAP2/LRRK2/NRXN1	ProbandFGC
3	GO:0007051	spindle organization	0.001662723	CLASP2/EML1/RMDN1/TTK/TUBGCP6	ProbandFGC
3	GO:0099565	chemical synaptic transmission, postsynaptic	0.001870986	BAIAP2/LRRK2/NRXN1/RIMS1	ProbandFGC
3	GO:0045666	positive regulation of neuron differentiation	0.001995292	ARMCX5-GPRASP2/BAIAP2/CPEB3/FBXO38/FMR1/RIMS1/STK25	ProbandFGC
3	GO:0098693	regulation of synaptic vesicle cycle	0.001996056	FMR1/LRRK2/NRXN1/RIMS1	ProbandFGC
3	GO:1902275	regulation of chromatin organization	0.002194414	DMRTC2/FMR1/KMT2B/LRRK2/TRIP12	ProbandFGC
3	GO:0007052	mitotic spindle organization	0.002263342	CLASP2/EML1/RMDN1/TTK	ProbandFGC
3	GO:0007127	meiosis I	0.002333831	CNTD1/DMRTC2/MEI1/TEX15	ProbandFGC
3	GO:0046660	female sex differentiation	0.002333831	KMT2B/LHX8/MERTK/ZFPM2	ProbandFGC
3	GO:0007179	transforming growth factor beta receptor signaling pathway	0.002556628	BCL9L/CDKN1C/CREBBP/ITGB5/LTBP1	ProbandFGC
3	GO:0061982	meiosis I cell cycle process	0.002709077	CNTD1/DMRTC2/MEI1/TEX15	ProbandFGC
3	GO:0017015	regulation of transforming growth factor beta receptor signaling pathway	0.00295304	BCL9L/CDKN1C/CREBBP/LTBP1	ProbandFGC
3	GO:1903844	regulation of cellular response to transforming growth factor beta stimulus	0.003123784	BCL9L/CDKN1C/CREBBP/LTBP1	ProbandFGC
3	GO:0051656	establishment of organelle localization	0.004060784	ACTR3/CLASP2/LRRK2/MEI1/PARD3B/RMDN1/STK25	ProbandFGC
3	GO:0016571	histone methylation	0.004511339	CREBBP/DMRTC2/KMT2B/SATB1	ProbandFGC
3	GO:0060078	regulation of postsynaptic membrane potential	0.004623967	BAIAP2/LRRK2/NRXN1/RIMS1	ProbandFGC
3	GO:1902850	microtubule cytoskeleton organization involved in mitosis	0.004623967	CLASP2/EML1/RMDN1/TTK	ProbandFGC
3	GO:0042542	response to hydrogen peroxide	0.004854878	LRRK2/PPP1R15B/STAT6/STK25	ProbandFGC
3	GO:0016358	dendrite development	0.005993591	ARMCX5-GPRASP2/BAIAP2/CPEB3/FMR1/LRRK2	ProbandFGC
3	GO:0051017	actin filament bundle assembly	0.006264346	BAIAP2/CLASP2/ITGB5/TMEFF2	ProbandFGC
3	GO:0033044	regulation of chromosome organization	0.00641178	DMRTC2/FMR1/KMT2B/LRRK2/TRIP12/TTK	ProbandFGC
3	GO:0071560	cellular response to transforming growth factor beta stimulus	0.006512111	BCL9L/CDKN1C/CREBBP/ITGB5/LTBP1	ProbandFGC
3	GO:0007178	transmembrane receptor protein serine/threonine kinase signaling pathway	0.006671327	BCL9L/CDKN1C/CREBBP/ITGB5/LTBP1/TTK	ProbandFGC
3	GO:0031589	cell-substrate adhesion	0.006671327	CLASP2/ITGB5/MERTK/RADIL/TMEFF2/VWF	ProbandFGC
3	GO:0090092	regulation of transmembrane receptor protein serine/ threonine kinase signaling pathway	0.006728085	BCL9L/CDKN1C/CREBBP/LTBP1/TTK	ProbandFGC
3	GO:0061572	actin filament bundle organization	0.006837709	BAIAP2/CLASP2/ITGB5/TMEFF2	ProbandFGC
3	GO:0071559	response to transforming growth factor beta	0.007175037	BCL9L/CDKN1C/CREBBP/ITGB5/LTBP1	ProbandFGC
3	GO:0007611	learning or memory	0.007406132	ARMCX5-GPRASP2/CPEB3/KMT2B/LHX8/NRXN1	ProbandFGC
3	GO:0050769	positive regulation of neurogenesis	0.007534994	ARMCX5-GPRASP2/BAIAP2/CPEB3/FBXO38/FMR1/RIMS1/STK25	ProbandFGC
3	GO:0050806	positive regulation of synaptic transmission	0.008591	BAIAP2/FMR1/NRXN1/RIMS1	ProbandFGC
3	GO:0099173	postsynapse organization	0.008763623	ARMCX5-GPRASP2/BAIAP2/LRRK2/NRXN1	ProbandFGC
3	GO:0098813	nuclear chromosome segregation	0.00890297	ACTR3/MEI1/RMDN1/TEX15/TTK	ProbandFGC
3	GO:0007033	vacuole organization	0.008938503	ATG2B/LRRK2/PACS2/UBQLN1	ProbandFGC
3	GO:0007269	neurotransmitter secretion	0.009115652	FMR1/LRRK2/NRXN1/RIMS1	ProbandFGC
3	GO:0099643	signal release from synapse	0.009115652	FMR1/LRRK2/NRXN1/RIMS1	ProbandFGC
3	GO:0031331	positive regulation of cellular catabolic process	0.009819071	CPEB3/FMR1/LRRK2/PLIN5/UBQLN1/VPS13C	ProbandFGC
4	GO:0010812	negative regulation of cell-substrate adhesion	9.97E-05	FZD7/JAG1/NOTCH1/PLG	ProbandFGC
4	GO:0050673	epithelial cell proliferation	0.000195903	FZD7/LGR4/NOTCH1/PEX2/PRKCA/PROX1/PTPRK/TP63	ProbandFGC
4	GO:0035051	cardiocyte differentiation	0.000245734	FZD7/JAG1/NEB/NOTCH1/PROX1	ProbandFGC
4	GO:0019827	stem cell population maintenance	0.000275873	FZD7/JAG1/NOTCH1/PROX1/TP63	ProbandFGC
4	GO:0055006	cardiac cell development	0.000282703	JAG1/NEB/NOTCH1/PROX1	ProbandFGC
4	GO:0098727	maintenance of cell number	0.000291956	FZD7/JAG1/NOTCH1/PROX1/TP63	ProbandFGC
4	GO:0048771	tissue remodeling	0.000425349	JAG1/LGR4/PLG/PRKCA/TCIRG1	ProbandFGC
4	GO:0050678	regulation of epithelial cell proliferation	0.000493612	FZD7/NOTCH1/PEX2/PRKCA/PROX1/PTPRK/TP63	ProbandFGC
4	GO:0008593	regulation of Notch signaling pathway	0.000589947	JAG1/NOTCH1/SREBF2/TP63	ProbandFGC
4	GO:0051146	striated muscle cell differentiation	0.00062292	FZD7/GDF15/NEB/NOTCH1/PROX1/RYR1	ProbandFGC
4	GO:0003279	cardiac septum development	0.000722963	JAG1/NOTCH1/PCSK5/PROX1	ProbandFGC
4	GO:0051153	regulation of striated muscle cell differentiation	0.000848988	FZD7/GDF15/NOTCH1/PROX1	ProbandFGC
4	GO:0002065	columnar/cuboidal epithelial cell differentiation	0.000903483	JAG1/NOTCH1/PROX1/TP63	ProbandFGC
4	GO:0050679	positive regulation of epithelial cell proliferation	0.00091814	FZD7/NOTCH1/PRKCA/PROX1/TP63	ProbandFGC
4	GO:0010810	regulation of cell-substrate adhesion	0.001040599	EMP2/FZD7/JAG1/NOTCH1/PLG	ProbandFGC
4	GO:0001655	urogenital system development	0.001114269	JAG1/LGR4/NOTCH1/PCSK5/PROX1/TP63	ProbandFGC
4	GO:0045667	regulation of osteoblast differentiation	0.001213448	JAG1/NOTCH1/TCIRG1/TP63	ProbandFGC
4	GO:0048565	digestive tract development	0.001356022	LGR4/NOTCH1/PCSK5/TP63	ProbandFGC
4	GO:0001649	osteoblast differentiation	0.001373221	JAG1/LGR4/NOTCH1/TCIRG1/TP63	ProbandFGC
4	GO:0031589	cell-substrate adhesion	0.001711369	EMP2/FZD7/JAG1/NOTCH1/PLG/PTPRK	ProbandFGC
4	GO:0055123	digestive system development	0.001853528	LGR4/NOTCH1/PCSK5/TP63	ProbandFGC
4	GO:2000027	regulation of animal organ morphogenesis	0.002081779	DAAM1/FZD7/JAG1/LGR4/NOTCH1	ProbandFGC
4	GO:0014706	striated muscle tissue development	0.002558457	FZD7/GTF3C5/NEB/NOTCH1/PROX1/RYR1	ProbandFGC
4	GO:0042692	muscle cell differentiation	0.002591285	FZD7/GDF15/NEB/NOTCH1/PROX1/RYR1	ProbandFGC
4	GO:0007517	muscle organ development	0.003199365	FZD7/GTF3C5/NEB/NOTCH1/PROX1/RYR1	ProbandFGC
4	GO:0060537	muscle tissue development	0.003277364	FZD7/GTF3C5/NEB/NOTCH1/PROX1/RYR1	ProbandFGC
4	GO:0001822	kidney development	0.003317386	JAG1/LGR4/NOTCH1/PCSK5/PROX1	ProbandFGC
4	GO:0001503	ossification	0.003397019	JAG1/LGR4/NOTCH1/RYR1/TCIRG1/TP63	ProbandFGC
4	GO:0003205	cardiac chamber development	0.00340964	JAG1/NOTCH1/PCSK5/PROX1	ProbandFGC
4	GO:0072001	renal system development	0.003790187	JAG1/LGR4/NOTCH1/PCSK5/PROX1	ProbandFGC
4	GO:0043588	skin development	0.003953629	JAG1/KRT10/LGR4/NOTCH1/RYR1/TP63	ProbandFGC
4	GO:0055001	muscle cell development	0.00408076	NEB/NOTCH1/PROX1/RYR1	ProbandFGC
4	GO:0051147	regulation of muscle cell differentiation	0.004322972	FZD7/GDF15/NOTCH1/PROX1	ProbandFGC
4	GO:0061138	morphogenesis of a branching epithelium	0.004405822	LGR4/NOTCH1/PROX1/TP63	ProbandFGC
4	GO:0007219	Notch signaling pathway	0.005107556	JAG1/NOTCH1/SREBF2/TP63	ProbandFGC
4	GO:0048568	embryonic organ development	0.005266855	EN2/MYO3B/NOTCH1/PCSK5/PLG/PROX1	ProbandFGC
4	GO:0048839	inner ear development	0.005294033	JAG1/MYO3B/NOTCH1/PROX1	ProbandFGC
4	GO:0019933	cAMP-mediated signaling	0.005485028	ADCY5/ADCYAP1R1/LGR4/PRKCA	ProbandFGC
4	GO:0001763	morphogenesis of a branching structure	0.005680587	LGR4/NOTCH1/PROX1/TP63	ProbandFGC
4	GO:0031099	regeneration	0.005680587	FZD7/NOTCH1/PLG/PTPN3	ProbandFGC
4	GO:0051056	regulation of small GTPase mediated signal transduction	0.005790862	ADCYAP1R1/ARHGAP10/ARHGEF3/CHN1/NOTCH1	ProbandFGC
4	GO:0030278	regulation of ossification	0.006839877	JAG1/NOTCH1/TCIRG1/TP63	ProbandFGC
4	GO:0008544	epidermis development	0.0068764	EDA2R/JAG1/KRT10/LGR4/NOTCH1/TP63	ProbandFGC
4	GO:0002064	epithelial cell development	0.007897553	JAG1/NOTCH1/PROX1/TP63	ProbandFGC
4	GO:0019935	cyclic-nucleotide-mediated signaling	0.00827271	ADCY5/ADCYAP1R1/LGR4/PRKCA	ProbandFGC
4	GO:0043583	ear development	0.00827271	JAG1/MYO3B/NOTCH1/PROX1	ProbandFGC
4	GO:0048738	cardiac muscle tissue development	0.009193192	FZD7/NEB/NOTCH1/PROX1	ProbandFGC
4	GO:0007188	adenylate cyclase-modulating G protein-coupled receptor signaling pathway	0.009467973	ADCY5/ADCYAP1R1/LGR4/PRKCA	ProbandFGC
5	GO:0043368	positive T cell selection	5.39E-05	CD74/MTOR/SRF/STAT6	ProbandFollicle
5	GO:0030217	T cell differentiation	0.000110815	CD74/JAK3/MTOR/NKX2-3/SRF/STAT6/TCIRG1/ZMIZ1	ProbandFollicle
5	GO:0045667	regulation of osteoblast differentiation	0.000112734	FAM20C/FBN2/LRP3/NOTCH1/PDLIM7/TCIRG1	ProbandFollicle
5	GO:0006383	transcription by RNA polymerase III	0.0001553	GTF3C1/GTF3C5/MTOR/SNAPC2	ProbandFollicle
5	GO:0045058	T cell selection	0.000182849	CD74/MTOR/SRF/STAT6	ProbandFollicle
5	GO:0035710	CD4-positive, alpha-beta T cell activation	0.000248029	JAK3/MTOR/NKX2-3/STAT6/TCIRG1	ProbandFollicle
5	GO:0045669	positive regulation of osteoblast differentiation	0.000576876	FAM20C/FBN2/LRP3/PDLIM7	ProbandFollicle
5	GO:0043367	CD4-positive, alpha-beta T cell differentiation	0.001082779	JAK3/MTOR/NKX2-3/STAT6	ProbandFollicle
5	GO:0030098	lymphocyte differentiation	0.001333292	CD74/JAK3/MTOR/NKX2-3/SRF/STAT6/TCIRG1/ZMIZ1	ProbandFollicle

5	GO:0030278	regulation of ossification	0.001453748	FAM20C/FBN2/LRP3/NOTCH1/PDLIM7/TCIRG1	ProbandFollicle
5	GO:0001776	leukocyte homeostasis	0.001556068	CD74/JAK3/NKX2-3/TCIRG1	ProbandFollicle
5	GO:0007193	adenylate cyclase-inhibiting G protein-coupled receptor signaling pathway	0.001556068	ADCY5/GRM6/OPRK1/PSAPL1	ProbandFollicle
5	GO:0046631	alpha-beta T cell activation	0.001588227	JAK3/MTOR/NKX2-3/STAT6/TCIRG1	ProbandFollicle
5	GO:0042475	odontogenesis of dentin-containing tooth	0.001915764	FAM20C/LHX8/NKX2-3/TCIRG1	ProbandFollicle
5	GO:0045778	positive regulation of ossification	0.002157189	FAM20C/FBN2/LRP3/PDLIM7	ProbandFollicle
5	GO:0051017	actin filament bundle assembly	0.002183161	BAIAP2L2/ITGB5/MTOR/NEDD9/SRF	ProbandFollicle
5	GO:0001649	osteoblast differentiation	0.00224146	FAM20C/FBN2/LRP3/NOTCH1/PDLIM7/TCIRG1	ProbandFollicle
5	GO:0061572	actin filament bundle organization	0.002435218	BAIAP2L2/ITGB5/MTOR/NEDD9/SRF	ProbandFollicle
5	GO:0006352	DNA-templated transcription, initiation	0.003250899	GTF3C1/GTF3C5/NOTCH1/POLR1A/SRF/TRIM28	ProbandFollicle
5	GO:0046632	alpha-beta T cell differentiation	0.003333444	JAK3/MTOR/NKX2-3/STAT6	ProbandFollicle
5	GO:0030099	myeloid cell differentiation	0.003544545	CD74/FAM20C/JAK3/MTOR/NBEAL2/NKX2-3/SRF/TCIRG1	ProbandFollicle
5	GO:0002286	T cell activation involved in immune response	0.003684209	CD74/JAK3/MTOR/STAT6	ProbandFollicle
5	GO:0098781	ncRNA transcription	0.003684209	GTF3C1/GTF3C5/MTOR/SNAPC2	ProbandFollicle
5	GO:0048872	homeostasis of number of cells	0.003723402	CD74/JAK3/NKX2-3/NOTCH1/SRF/TCIRG1	ProbandFollicle
6	GO:0010976	positive regulation of neuron projection development	4.38E-05	ARMCX5-GPRASP2/BMP5/CPEB3/DVL2/FMR1/MACF1/MAPT/PLXNA2/ROR2	ProbandFollicle
6	GO:0030177	positive regulation of Wnt signaling pathway	8.81E-05	LGR4/LRRK2/MACF1/ROR2/RSPO1/WNK1/WNT10B	ProbandFollicle
6	GO:0031346	positive regulation of cell projection organization	8.84E-05	ARMCX5-GPRASP2/BCAS3/BMP5/CPEB3/DVL2/FMR1/MACF1/MAPT/PLXNA2/ROR2	ProbandFollicle
6	GO:0048806	genitalia development	0.000149873	BMP5/LGR4/ROR2/TP63	ProbandFollicle
6	GO:0031589	cell-substrate adhesion	0.000221165	ATXN3/BCAS3/EMP2/FZD7/JAG1/LIMS1/MACF1/TMEFF2/VWF	ProbandFollicle
6	GO:0090263	positive regulation of canonical Wnt signaling pathway	0.000224525	LGR4/LRRK2/ROR2/RSPO1/WNK1/WNT10B	ProbandFollicle
6	GO:0010810	regulation of cell-substrate adhesion	0.000252309	ATXN3/BCAS3/EMP2/FZD7/JAG1/LIMS1/MACF1	ProbandFollicle
6	GO:0060828	regulation of canonical Wnt signaling pathway	0.000296027	DVL2/FZD7/LGR4/LRRK2/ROR2/RSPO1/WNK1/WNT10B	ProbandFollicle
6	GO:0030111	regulation of Wnt signaling pathway	0.000304946	DVL2/FZD7/LGR4/LRRK2/MACF1/ROR2/RSPO1/WNK1/WNT10B	ProbandFollicle
6	GO:0045666	positive regulation of neuron differentiation	0.000335968	ARMCX5-GPRASP2/BMP5/CPEB3/DVL2/FMR1/MACF1/MAPT/PLXNA2/ROR2	ProbandFollicle
6	GO:0002011	morphogenesis of an epithelial sheet	0.000339693	BMP5/DVL2/JAG1/TMEFF2	ProbandFollicle
6	GO:0043087	regulation of GTPase activity	0.000439779	BCAS3/CHN1/DOCK4/DVL2/LIMS1/LRRK2/PLXNA2/RASA2/SBF2/WNK1	ProbandFollicle
6	GO:0060070	canonical Wnt signaling pathway	0.00074147	DVL2/FZD7/LGR4/LRRK2/ROR2/RSPO1/WNK1/WNT10B	ProbandFollicle
6	GO:0001952	regulation of cell-matrix adhesion	0.000761992	BCAS3/EMP2/JAG1/LIMS1/MACF1	ProbandFollicle
6	GO:1903747	regulation of establishment of protein localization to mitochondrion	0.000816027	LRRK2/MAPT/SREBF2/TP63	ProbandFollicle
6	GO:0050805	negative regulation of synaptic transmission	0.000903318	FMR1/LRRK2/MAPT/SLC6A1	ProbandFollicle
6	GO:1900006	positive regulation of dendrite development	0.000903318	ARMCX5-GPRASP2/BMP5/CPEB3/FMR1	ProbandFollicle
6	GO:0060998	regulation of dendritic spine development	0.001046296	ARMCX5-GPRASP2/CPEB3/FMR1/LRRK2	ProbandFollicle
6	GO:0003151	outflow tract morphogenesis	0.001150004	DVL2/ELN/JAG1/RYR1	ProbandFollicle
6	GO:0046330	positive regulation of JNK cascade	0.001187686	DVL2/EDA2R/FZD7/MMP8/ROR2	ProbandFollicle
6	GO:0035637	multicellular organismal signaling	0.001197143	ATP2B3/FMR1/KCND3/NPR1/RYR1/SCN9A	ProbandFollicle
6	GO:0050769	positive regulation of neurogenesis	0.001878911	ARMCX5-GPRASP2/BMP5/CPEB3/DVL2/FMR1/MACF1/MAPT/PLXNA2/ROR2	ProbandFollicle
6	GO:0050773	regulation of dendrite development	0.001982759	ARMCX5-GPRASP2/BMP5/CPEB3/FMR1/LRRK2	ProbandFollicle
6	GO:0010769	regulation of cell morphogenesis involved in differentiation	0.002047495	ARMCX5-GPRASP2/CHN1/LIMS1/LRRK2/MACF1/MAPT/PLXNA2	ProbandFollicle
6	GO:0010770	positive regulation of cell morphogenesis involved in differentiation	0.00209686	ARMCX5-GPRASP2/LIMS1/MACF1/MAPT/PLXNA2	ProbandFollicle
6	GO:0035282	segmentation	0.002612911	DVL2/MEOX1/PLXNA2/ROR2	ProbandFollicle
6	GO:0060996	dendritic spine development	0.002807144	ARMCX5-GPRASP2/CPEB3/FMR1/LRRK2	ProbandFollicle
6	GO:0032874	positive regulation of stress-activated MAPK cascade	0.002958802	DVL2/EDA2R/FZD7/MMP8/ROR2	ProbandFollicle
6	GO:0070304	positive regulation of stress-activated protein kinase signaling cascade	0.003111132	DVL2/EDA2R/FZD7/MMP8/ROR2	ProbandFollicle
6	GO:0008593	regulation of Notch signaling pathway	0.00344879	CREBBP/JAG1/SREBF2/TP63	ProbandFollicle
6	GO:0007611	learning or memory	0.003837576	ARMCX5-GPRASP2/CPEB3/MAPT/NRXN1/NRXN3/SLC6A1	ProbandFollicle
6	GO:0046328	regulation of JNK cascade	0.004146428	DVL2/EDA2R/FZD7/MMP8/ROR2	ProbandFollicle
6	GO:0050804	modulation of chemical synaptic transmission	0.004574908	CPEB3/DLGAP2/FMR1/LRRK2/MAPT/NRXN1/ROR2/SLC6A1	ProbandFollicle
6	GO:0099177	regulation of trans-synaptic signaling	0.004635091	CPEB3/DLGAP2/FMR1/LRRK2/MAPT/NRXN1/ROR2/SLC6A1	ProbandFollicle
6	GO:0016570	histone modification	0.005474303	ATXN3/CREBBP/FMR1/LRRK2/PRKCA/PRMT2/SATB1/SRCAP	ProbandFollicle
6	GO:0001736	establishment of planar polarity	0.005631725	DVL2/FZD7/ROR2/TP63	ProbandFollicle
6	GO:0007164	establishment of tissue polarity	0.005631725	DVL2/FZD7/ROR2/TP63	ProbandFollicle
6	GO:1901888	regulation of cell junction assembly	0.005882324	ARMCX5-GPRASP2/BCAS3/LIMS1/MACF1/NRXN1	ProbandFollicle
6	GO:0021700	developmental maturation	0.006171247	IGSF21/LRRK2/NRXN1/REC8/RYR1/WNT10B	ProbandFollicle
6	GO:0016569	covalent chromatin modification	0.006425282	ATXN3/CREBBP/FMR1/LRRK2/PRKCA/PRMT2/SATB1/SRCAP	ProbandFollicle
7	GO:0007127	meiosis I	0.000190381	CNTD1/DMRTC2/ESPL1/ING2/MEI1	ProbandFollicle
7	GO:0061982	meiosis I cell cycle process	0.000230657	CNTD1/DMRTC2/ESPL1/ING2/MEI1	ProbandFollicle
7	GO:0022604	regulation of cell morphogenesis	0.00037377	ARHGAP33/BAIAP2/BCL9L/EEF2K/HECW2/MFSD2A/PLXNA1/ROBO2/TPM1	ProbandFollicle
7	GO:0031056	regulation of histone modification	0.000537202	DMRTC2/GLYR1/ING2/KMT2B/TRIP12	ProbandFollicle
7	GO:0008643	carbohydrate transport	0.000570789	MFSD2A/RTN2/SLC5A1/SLC5A10/ZDHHC7	ProbandFollicle
7	GO:0050773	regulation of dendrite development	0.000642669	ARHGAP33/BAIAP2/EEF2K/HECW2/MFSD2A	ProbandFollicle
7	GO:0048814	regulation of dendrite morphogenesis	0.000855612	ARHGAP33/BAIAP2/EEF2K/HECW2	ProbandFollicle
7	GO:0033044	regulation of chromosome organization	0.00104746	DMRTC2/ESPL1/GLYR1/HECW2/ING2/KMT2B/TRIP12	ProbandFollicle
7	GO:0140013	meiotic nuclear division	0.001165919	CNTD1/DMRTC2/ESPL1/ING2/MEI1	ProbandFollicle
7	GO:1904659	glucose transmembrane transport	0.001475254	RTN2/SLC5A1/SLC5A10/ZDHHC7	ProbandFollicle
7	GO:0008645	hexose transmembrane transport	0.00168239	RTN2/SLC5A1/SLC5A10/ZDHHC7	ProbandFollicle
7	GO:1903046	meiotic cell cycle process	0.00170858	CNTD1/DMRTC2/ESPL1/ING2/MEI1	ProbandFollicle
7	GO:1902275	regulation of chromatin organization	0.001787539	DMRTC2/GLYR1/ING2/KMT2B/TRIP12	ProbandFollicle
7	GO:0015749	monosaccharide transmembrane transport	0.001793179	RTN2/SLC5A1/SLC5A10/ZDHHC7	ProbandFollicle
7	GO:0034219	carbohydrate transmembrane transport	0.001908934	RTN2/SLC5A1/SLC5A10/ZDHHC7	ProbandFollicle
7	GO:0010769	regulation of cell morphogenesis involved in differentiation	0.002605808	ARHGAP33/BAIAP2/EEF2K/HECW2/PLXNA1/ROBO2	ProbandFollicle
7	GO:0003206	cardiac chamber morphogenesis	0.002790206	MYH6/PROX1/ROBO2/TPM1	ProbandFollicle
7	GO:0003231	cardiac ventricle development	0.002790206	MYH6/PROX1/ROBO2/TPM1	ProbandFollicle
7	GO:0000280	nuclear division	0.002974294	CNTD1/DMRTC2/ESPL1/HECW2/ING2/MEI1/RMDN1	ProbandFollicle
7	GO:0048813	dendrite morphogenesis	0.004113554	ARHGAP33/BAIAP2/EEF2K/HECW2	ProbandFollicle
7	GO:0046777	protein autophosphorylation	0.004139219	DAPK2/EEF2K/EIF2AK1/EIF2AK4/FLT3	ProbandFollicle
7	GO:0016358	dendrite development	0.004925661	ARHGAP33/BAIAP2/EEF2K/HECW2/MFSD2A	ProbandFollicle
7	GO:0048285	organelle fission	0.005306339	CNTD1/DMRTC2/ESPL1/HECW2/ING2/MEI1/RMDN1	ProbandFollicle
7	GO:0010770	positive regulation of cell morphogenesis involved in differentiation	0.005317344	BAIAP2/EEF2K/PLXNA1/ROBO2	ProbandFollicle
7	GO:0051321	meiotic cell cycle	0.005535823	CNTD1/DMRTC2/ESPL1/ING2/MEI1	ProbandFollicle
7	GO:0022412	cellular process involved in reproduction in multicellular organism	0.006557289	DMRTC2/ING2/KMT2B/MEI1/ROBO2/TSSK1B	ProbandFollicle
7	GO:0045666	positive regulation of neuron differentiation	0.006980305	BAIAP2/EEF2K/FBXO38/PLXNA1/PROX1/ROBO2	ProbandFollicle
7	GO:0003205	cardiac chamber development	0.007608237	MYH6/PROX1/ROBO2/TPM1	ProbandFollicle
7	GO:0035725	sodium ion transmembrane transport	0.007608237	HECW2/SCNN1D/SLC5A1/SLC5A10	ProbandFollicle
7	GO:0007281	germ cell development	0.00768535	DMRTC2/ING2/KMT2B/MEI1/TSSK1B	ProbandFollicle
7	GO:0007548	sex differentiation	0.008272236	ADCYAP1R1/DMRTC2/ING2/KMT2B/ROBO2	ProbandFollicle
7	GO:2001252	positive regulation of chromosome organization	0.008880872	DMRTC2/ESPL1/GLYR1/ING2	ProbandFollicle
7	GO:0010976	positive regulation of neuron projection development	0.009538228	BAIAP2/EEF2K/FBXO38/PLXNA1/ROBO2	ProbandFollicle
7	GO:0007517	muscle organ development	0.009614206	BCL9L/EVC/MRAS/MYH6/PROX1/TPM1	ProbandFollicle
9	GO:0030511	positive regulation of transforming growth factor beta receptor signaling pathway	0.000101694	CDKN1C/CREBBP/ING2/SNW1	ProbandOocyte
9	GO:1903846	positive regulation of cellular response to transforming growth factor beta stimulus	0.000101694	CDKN1C/CREBBP/ING2/SNW1	ProbandOocyte
9	GO:0030239	myofibril assembly	0.000224828	NEB/OBSCN/SRF/TMOD4/TPM1	ProbandOocyte
9	GO:0007179	transforming growth factor beta receptor signaling pathway	0.000269968	BCL9L/CDKN1C/CREBBP/FBN2/GDF15/ING2/PTPRK/SNW1	ProbandOocyte
9	GO:0090100	positive regulation of transmembrane receptor protein serine /threonine kinase signaling pathway	0.000295972	BMP5/CDKN1C/CREBBP/GDF15/ING2/SNW1	ProbandOocyte
9	GO:0003197	endocardial cushion development	0.00050246	BMP5/CRELD1/DCHS1/ROBO2	ProbandOocyte
9	GO:0006383	transcription by RNA polymerase III	0.0005939	GTF3C1/GTF3C5/MTOR/SNAPC2	ProbandOocyte
9	GO:0017015	regulation of transforming growth factor beta receptor signaling pathway	0.0006373	BCL9L/CDKN1C/CREBBP/FBN2/ING2/SNW1	ProbandOocyte
9	GO:1903844	regulation of cellular response to transforming growth factor beta stimulus	0.000691919	BCL9L/CDKN1C/CREBBP/FBN2/ING2/SNW1	ProbandOocyte
9	GO:0007178	transmembrane receptor protein serine/threonine kinase signaling pathway	0.000785785	BCL9L/BMP5/CDKN1C/CREBBP/FBN2/GDF15/ING2/PTPRK/ROR2/SNW1	ProbandOocyte
9	GO:0003179	heart valve morphogenesis	0.000873228	DCHS1/ELN/MTOR/ROBO2	ProbandOocyte
9	GO:0071560	cellular response to transforming growth factor beta stimulus	0.001155344	BCL9L/CDKN1C/CREBBP/FBN2/GDF15/ING2/PTPRK/SNW1	ProbandOocyte
9	GO:0090092	regulation of transmembrane receptor protein serine /threonine kinase signaling pathway	0.001215086	BCL9L/BMP5/CDKN1C/CREBBP/FBN2/GDF15/ING2/SNW1	ProbandOocyte
9	GO:0071559	response to transforming growth factor beta	0.001341961	BCL9L/CDKN1C/CREBBP/FBN2/GDF15/ING2/PTPRK/SNW1	ProbandOocyte
9	GO:0031032	actomyosin structure organization	0.001344512	ELN/MTOR/NEB/OBSCN/SRF/TMOD4/TPM1	ProbandOocyte
9	GO:0014706	striated muscle tissue development	0.001437327	BCL9L/BMP5/ELN/FZD7/GTF3C5/MTOR/MYOM2/NEB/SRF/TPM1	ProbandOocyte
10	GO:0050890	cognition	0.000172619	ARMCX5-GPRASP2/ATXN1/CPEB3/EIF2AK4/GPR155/ KMT2B/MAPT/NRXN1/NRXN3/SLC6A1	ProbandOocyte
10	GO:0010769	regulation of cell morphogenesis involved in differentiation	0.000213116	ARHGAP33/ARMCX5-GPRASP2/CUL7/HECW2/LRRK2/ MACF1/MAP2/MAPT/PLXNA1/SKIL	ProbandOocyte
10	GO:0003007	heart morphogenesis	0.000249506	ACVR1/DVL2/HTR2B/MIB1/MYH6/NOTCH1/RYR1/ZFPM2/ZMIZ1	ProbandOocyte
10	GO:0050773	regulation of dendrite development	0.000263793	ARHGAP33/ARMCX5-GPRASP2/CPEB3/CUL7/FMR1/HECW2/LRRK2	ProbandOocyte
10	GO:0007611	learning or memory	0.000264144	ARMCX5-GPRASP2/ATXN1/CPEB3/EIF2AK4/KMT2B/MAPT/NRXN1/NRXN3/SLC6A1	ProbandOocyte
10	GO:0001701	in utero embryonic development	0.000281416	ACVR1/DSC3/FURIN/MIB1/MYH6/NOTCH1/PLG/SKIL/TRIM28/ZFPM2/ZMIZ1	ProbandOocyte
10	GO:0031346	positive regulation of cell projection organization	0.000357824	ACTR3/ARMCX5-GPRASP2/BCAS3/CPEB3/CUL7/ DVL2/FMR1/MACF1/MAPT/PLXNA1/SKIL	ProbandOocyte
10	GO:0060998	regulation of dendritic spine development	0.000418048	ARHGAP33/ARMCX5-GPRASP2/CPEB3/FMR1/LRRK2	ProbandOocyte
10	GO:0007613	memory	0.000444699	ATXN1/CPEB3/EIF2AK4/KMT2B/MAPT/SLC6A1	ProbandOocyte
10	GO:0019079	viral genome replication	0.000574996	CTBP2/EIF2AK4/FMR1/NOTCH1/OASL/TRIM28	ProbandOocyte
10	GO:0010976	positive regulation of neuron projection development	0.000584246	ARMCX5-GPRASP2/CPEB3/CUL7/DVL2/FMR1/MACF1/MAPT/PLXNA1/SKIL	ProbandOocyte
10	GO:0016358	dendrite development	0.000895332	ARHGAP33/ARMCX5-GPRASP2/CPEB3/CUL7/FMR1/HECW2/LRRK2/MAP2	ProbandOocyte
10	GO:0070588	calcium ion transmembrane transport	0.000980672	ATP2B3/CACNA1B/CEMIP/FMR1/HTR2B/HTR2C/ORAI3/RYR1/TMEM37	ProbandOocyte
10	GO:0048814	regulation of dendrite morphogenesis	0.001028462	ARHGAP33/ARMCX5-GPRASP2/CUL7/HECW2/LRRK2	ProbandOocyte
10	GO:0048813	dendrite morphogenesis	0.001190284	ARHGAP33/ARMCX5-GPRASP2/CUL7/HECW2/LRRK2/MAP2	ProbandOocyte
10	GO:0002011	morphogenesis of an epithelial sheet	0.001225666	CLASP2/DVL2/NOTCH1/TMEFF2	ProbandOocyte

10	GO:0045069	regulation of viral genome replication	0.001294141	EIF2AK4/FMR1/NOTCH1/OASL/TRIM28	ProbandOocyte
10	GO:0060996	dendritic spine development	0.001413464	ARHGAP33/ARMCX5-GPRASP2/CPEB3/FMR1/LRRK2	ProbandOocyte
10	GO:0010770	positive regulation of cell morphogenesis involved in differentiation	0.001725174	ARMCX5-GPRASP2/CUL7/MACF1/MAPT/PLXNA1/SKIL	ProbandOocyte
10	GO:1903900	regulation of viral life cycle	0.001725174	CD74/EIF2AK4/FMR1/NOTCH1/OASL/TRIM28	ProbandOocyte
10	GO:0045669	positive regulation of osteoblast differentiation	0.001981116	ACVR1/FAM20C/TP63/WNT10B	ProbandOocyte
10	GO:0010812	negative regulation of cell-substrate adhesion	0.002210972	BCAS3/CLASP2/NOTCH1/PLG	ProbandOocyte
10	GO:0006816	calcium ion transport	0.002417343	ATP2B3/CACNA1B/CEMIP/FMR1/HTR2B/ HTR2C/ORAI3/PLCZ1/RYR1/TMEM37	ProbandOocyte
10	GO:0022604	regulation of cell morphogenesis	0.002417866	ARHGAP33/ARMCX5-GPRASP2/CUL7/DVL2/ HECW2/LRRK2/MACF1/MAP2/MAPT/PLXNA1/SKIL	ProbandOocyte
10	GO:0060485	mesenchyme development	0.002463984	ACVR1/CLASP2/CUL7/HTR2B/MEOX1/NOTCH1/TRIM28/ZFPM2	ProbandOocyte
10	GO:0001649	osteoblast differentiation	0.002718813	ACVR1/FAM20C/LGR4/NOTCH1/TP63/VCAN/WNT10B	ProbandOocyte
10	GO:1903747	regulation of establishment of protein localization to mitochondrion	0.002864778	LRRK2/MAPT/SREBF2/TP63	ProbandOocyte
10	GO:0060560	developmental growth involved in morphogenesis	0.003061514	EIF2AK4/MACF1/MAP2/MAPT/NOTCH1/PLXNA1/TRIM28	ProbandOocyte
10	GO:0050805	negative regulation of synaptic transmission	0.003159709	FMR1/LRRK2/MAPT/SLC6A1	ProbandOocyte
10	GO:1900006	positive regulation of dendrite development	0.003159709	ARMCX5-GPRASP2/CPEB3/CUL7/FMR1	ProbandOocyte
10	GO:0003281	ventricular septum development	0.00331466	ACVR1/LTBP1/NOTCH1/ZFPM2	ProbandOocyte
10	GO:0030177	positive regulation of Wnt signaling pathway	0.003701327	LGR4/LRRK2/MACF1/RSPO1/WNK1/WNT10B	ProbandOocyte
10	GO:0045666	positive regulation of neuron differentiation	0.003729509	ARMCX5-GPRASP2/CPEB3/CUL7/DVL2/FMR1/MACF1/MAPT/PLXNA1/SKIL	ProbandOocyte
10	GO:0006446	regulation of translational initiation	0.003810414	EIF2AK1/EIF2AK4/FMR1/PPP1R15B	ProbandOocyte
10	GO:0003151	outflow tract morphogenesis	0.003986228	DVL2/NOTCH1/RYR1/ZFPM2	ProbandOocyte
10	GO:0003231	cardiac ventricle development	0.004194652	ACVR1/LTBP1/MYH6/NOTCH1/ZFPM2	ProbandOocyte
10	GO:0045667	regulation of osteoblast differentiation	0.004194652	ACVR1/FAM20C/NOTCH1/TP63/WNT10B	ProbandOocyte
10	GO:0022617	extracellular matrix disassembly	0.004354164	CLASP2/FURIN/MMP8/PLG	ProbandOocyte
10	GO:0090101	negative regulation of transmembrane receptor protein serine/threonine kinase signaling pathway	0.004472631	ACVR1/LTBP1/NOTCH1/NRROS/SKIL	ProbandOocyte
10	GO:0001570	vasculogenesis	0.00454644	CUL7/NOTCH1/ZFPM2/ZMIZ1	ProbandOocyte
10	GO:0070838	divalent metal ion transport	0.004911507	ATP2B3/CACNA1B/CEMIP/FMR1/HTR2B/HTR2C/ORAI3/PLCZ1/RYR1/TMEM37	ProbandOocyte
10	GO:0050772	positive regulation of axonogenesis	0.005157393	MACF1/MAPT/PLXNA1/SKIL	ProbandOocyte
10	GO:0001942	hair follicle development	0.005372666	LGR4/NOTCH1/TP63/WNT10B	ProbandOocyte
10	GO:0072511	divalent inorganic cation transport	0.005436107	ATP2B3/CACNA1B/CEMIP/FMR1/HTR2B/HTR2C/ORAI3/PLCZ1/RYR1/TMEM37	ProbandOocyte
10	GO:0046889	positive regulation of lipid biosynthetic process	0.005593869	CD74/ENPP7/HTR2B/HTR2C	ProbandOocyte
10	GO:0043087	regulation of GTPase activity	0.005673964	ARHGAP33/BCAS3/DOCK4/DVL2/HTR2B/LRRK2/PLXNA1/RASA2/SBF2/WNK1	ProbandOocyte
10	GO:0022404	molting cycle process	0.005821075	LGR4/NOTCH1/TP63/WNT10B	ProbandOocyte
10	GO:0022405	hair cycle process	0.005821075	LGR4/NOTCH1/TP63/WNT10B	ProbandOocyte
10	GO:0001763	morphogenesis of a branching structure	0.005822777	ACVR1/LGR4/LRRK2/NOTCH1/PLXNA1/TP63	ProbandOocyte
10	GO:0007179	transforming growth factor beta receptor signaling pathway	0.005962093	ACVR1/FURIN/LTBP1/NRROS/SKIL/ZMIZ1	ProbandOocyte
10	GO:0050769	positive regulation of neurogenesis	0.006003707	ARMCX5-GPRASP2/CPEB3/CUL7/DVL2/FMR1/MACF1/MAPT/NOTCH1/PLXNA1/SKIL	ProbandOocyte
10	GO:0098773	skin epidermis development	0.006054353	LGR4/NOTCH1/TP63/WNT10B	ProbandOocyte
10	GO:0007612	learning	0.006422176	ATXN1/EIF2AK4/NRXN1/NRXN3/SLC6A1	ProbandOocyte
10	GO:0019058	viral life cycle	0.00649654	CD74/CTBP2/EIF2AK4/FMR1/FURIN/NOTCH1/OASL/TRIM28	ProbandOocyte
10	GO:0031644	regulation of nervous system process	0.006989983	FMR1/HTR2C/LRRK2/MYRF/NRXN1	ProbandOocyte
10	GO:0045778	positive regulation of ossification	0.007049565	ACVR1/FAM20C/TP63/WNT10B	ProbandOocyte
10	GO:0098742	cell-cell adhesion via plasma-membrane adhesion molecules	0.007260562	ACVR1/CDHR5/DSC3/IGSF21/NRXN1/PCDH20/WNK1	ProbandOocyte
10	GO:0070167	regulation of biomineral tissue development	0.007314229	ACVR1/FAM20C/NOTCH1/WNT10B	ProbandOocyte
10	GO:0110149	regulation of biomineralization	0.007314229	ACVR1/FAM20C/NOTCH1/WNT10B	ProbandOocyte
10	GO:0090263	positive regulation of canonical Wnt signaling pathway	0.007387477	LGR4/LRRK2/RSPO1/WNK1/WNT10B	ProbandOocyte
10	GO:0007030	Golgi organization	0.007592022	BCAS3/CLASP2/CUL7/LMAN1L/LRRK2	ProbandOocyte
10	GO:0001837	epithelial to mesenchymal transition	0.007800484	ACVR1/CLASP2/CUL7/NOTCH1/TRIM28	ProbandOocyte
10	GO:0033044	regulation of chromosome organization	0.008323583	ESPL1/FMR1/HECW2/KMT2B/LRRK2/MAPT/TRIM28/YLPM1	ProbandOocyte
10	GO:0050792	regulation of viral process	0.008355388	CD74/EIF2AK4/FMR1/NOTCH1/OASL/TRIM28	ProbandOocyte
10	GO:0050808	synapse organization	0.008552181	ARHGAP33/ARMCX5-GPRASP2/CTBP2/IGSF21/LRRK2/MAPT/NRXN1/PPFIBP1/SLC6A1	ProbandOocyte
10	GO:0035282	segmentation	0.008735948	DVL2/MEOX1/MIB1/NOTCH1	ProbandOocyte
10	GO:0042116	macrophage activation	0.009040406	CD74/LRRK2/MAPT/MMP8	ProbandOocyte
10	GO:0007519	skeletal muscle tissue development	0.009135525	ANKRD33/NLN/NOTCH1/RYR1/WNT10B	ProbandOocyte
10	GO:0032388	positive regulation of intracellular transport	0.009287889	BCAS3/CEMIP/MAP2/SREBF2/TP63/TRIM28	ProbandOocyte
10	GO:0048608	reproductive structure development	0.009839197	CUL7/KMT2B/LGR4/NOTCH1/PLG/PSAPL1/TP63/TRIM28/ZFPM2	ProbandOocyte
11	GO:0045132	meiotic chromosome segregation	0.000129629	BUB1/MEI1/MLH3/TEX15/TTK	ProbandOocyte
11	GO:0140013	meiotic nuclear division	0.000341231	BUB1/DMRTC2/MEI1/MLH3/TEX15/TTK	ProbandOocyte
11	GO:0070192	chromosome organization involved in meiotic cell cycle	0.000501649	BUB1/MEI1/MLH3/TEX15	ProbandOocyte
11	GO:1903046	meiotic cell cycle process	0.00054013	BUB1/DMRTC2/MEI1/MLH3/TEX15/TTK	ProbandOocyte
12	GO:0007188	adenylate cyclase-modulating G protein-coupled receptor signaling pathway	3.67E-05	ADRA1A/CASR/MC5R/S1PR2	ProbandEmbryo
12	GO:0007187	G protein-coupled receptor signaling pathway, coupled to cyclic nucleotide second messenger	5.89E-05	ADRA1A/CASR/MC5R/S1PR2	ProbandEmbryo
14	GO:0043254	regulation of protein-containing complex assembly	0.003599038	CUL4B/EIF2AK2/PREX1/SPTBN5	ProbandEmbryo
1	GO:0007188	adenylate cyclase-modulating G protein-coupled receptor signaling pathway	0.000198379	ADRA1A/CASR/MC5R/S1PR2	SiblingFGC
1	GO:0007187	G protein-coupled receptor signaling pathway, coupled to cyclic nucleotide second messenger	0.000314852	ADRA1A/CASR/MC5R/S1PR2	SiblingFGC
1	GO:0051056	regulation of small GTPase mediated signal transduction	0.000683581	ADRA1A/ARHGAP28/ARHGEF2/PREX1	SiblingFGC
5	GO:0051056	regulation of small GTPase mediated signal transduction	0.000577382	ADRA1A/ARHGAP28/ARHGEF2/OBSCN	SiblingFollicle
5	GO:0010639	negative regulation of organelle organization	0.001479711	ARHGAP28/ARHGEF2/SPTBN5/TCHP	SiblingFollicle
12	GO:0007188	adenylate cyclase-modulating G protein-coupled receptor signaling pathway	3.67E-05	ADRA1A/CASR/MC5R/S1PR2	SiblingEmbryo
12	GO:0007187	G protein-coupled receptor signaling pathway, coupled to cyclic nucleotide second messenger	5.89E-05	ADRA1A/CASR/MC5R/S1PR2	SiblingEmbryo
14	GO:0043254	regulation of protein-containing complex assembly	0.003599038	CUL4B/EIF2AK2/PREX1/SPTBN5	SiblingEmbryo
2	GO:0045665	negative regulation of neuron differentiation	8.06E-05	CERS2/EDNRB/EIF2AK4/EPHA7/NLGN3/PAQR3/PLXNA3/SYNGAP1/THOC2	publicCTFGC
2	GO:0060560	developmental growth involved in morphogenesis	0.000115882	DBNL/EIF2AK4/EPHA7/KDM5B/LRP6/MACF1/NLGN3/PLXNA1/PLXNA3	publicCTFGC
2	GO:0031345	negative regulation of cell projection organization	0.000132043	CERS2/EPHA7/MAP4/NLGN3/PAQR3/PLXNA3/SYNGAP1/THOC2	publicCTFGC
2	GO:0070536	protein K63-linked deubiquitination	0.000165929	CYLD/STAMBP/USP8/VCP	publicCTFGC
2	GO:0010977	negative regulation of neuron projection development	0.000233822	CERS2/EPHA7/NLGN3/PAQR3/PLXNA3/SYNGAP1/THOC2	publicCTFGC
2	GO:0051961	negative regulation of nervous system development	0.000240755	CERS2/EDNRB/EIF2AK4/EPHA7/NLGN3/PAQR3/PLXNA3/RFX4/SYNGAP1/THOC2	publicCTFGC
2	GO:0001654	eye development	0.000256553	ATF4/CEP290/FBN1/FREM2/KDM5B/LCTL/LRP6/MYH10/MYOM2/NPHP4/PTPRM	publicCTFGC
2	GO:0150063	visual system development	0.000280285	ATF4/CEP290/FBN1/FREM2/KDM5B/LCTL/LRP6/MYH10/MYOM2/NPHP4/PTPRM	publicCTFGC
2	GO:0048880	sensory system development	0.000319347	ATF4/CEP290/FBN1/FREM2/KDM5B/LCTL/LRP6/MYH10/MYOM2/NPHP4/PTPRM	publicCTFGC
2	GO:0010721	negative regulation of cell development	0.000427407	CERS2/EDNRB/EIF2AK4/EPHA7/FBN1/NLGN3/PAQR3/PLXNA3/SYNGAP1/THOC2	publicCTFGC
2	GO:0060443	mammary gland morphogenesis	0.000443949	CAPN1/KDM5B/LRP6/PTCH1	publicCTFGC
2	GO:0099173	postsynapse organization	0.00047307	CAMK2B/DBNL/DOCK10/EPHA7/MYH10/NLGN3/SYNGAP1	publicCTFGC
2	GO:0050768	negative regulation of neurogenesis	0.000600016	CERS2/EDNRB/EIF2AK4/EPHA7/NLGN3/PAQR3/PLXNA3/SYNGAP1/THOC2	publicCTFGC
2	GO:0007018	microtubule-based movement	0.000736754	DNAH2/HTT/KIF14/KIF21A/KIF21B/KIF6/KLC2/MAP4/NPHP4/RFX3	publicCTFGC
2	GO:0007632	visual behavior	0.000830623	NLGN3/NPHP4/RIC8A/SYNGAP1	publicCTFGC
2	GO:0043010	camera-type eye development	0.001376162	ATF4/FBN1/KDM5B/LCTL/LRP6/MYH10/MYOM2/NPHP4/PTPRM	publicCTFGC
2	GO:0060997	dendritic spine morphogenesis	0.001497347	CAMK2B/DBNL/DOCK10/NLGN3	publicCTFGC
2	GO:0048592	eye morphogenesis	0.001561344	ATF4/CEP290/FBN1/LCTL/LRP6/PTPRM	publicCTFGC
2	GO:0007409	axonogenesis	0.001662431	DBNL/EPHA7/MACF1/MYH10/NLGN3/PLXNA1/PLXNA3/PTCH1/PTPRM/SYNGAP1/UNC5B	publicCTFGC
4	GO:0051899	membrane depolarization	1.04E-05	ABL1/CACNA1E/P2RX7/PTPN3/SCN1A/SCN5A/SLC8A1	publicCTFGC
4	GO:0006509	membrane protein ectodomain proteolysis	2.41E-05	ADAM10/MYH9/P2RX7/PSEN2/PTPN3	publicCTFGC
4	GO:0010256	endomembrane system organization	0.000142033	ANO6/ATP8B1/CSNK1D/DYNC2H1/MYH9/P2RX7/ RAB3GAP2/REEP2/SYNE1/VPS11/VPS18/VPS36/VPS4B	publicCTFGC
4	GO:0033619	membrane protein proteolysis	0.000148484	ADAM10/MYH9/P2RX7/PSEN2/PTPN3	publicCTFGC
4	GO:0086010	membrane depolarization during action potential	0.000240202	PTPN3/SCN1A/SCN5A/SLC8A1	publicCTFGC
4	GO:0045332	phospholipid translocation	0.000472046	ANO6/ATP10B/ATP8B1/P2RX7	publicCTFGC
4	GO:0034204	lipid translocation	0.000608423	ANO6/ATP10B/ATP8B1/P2RX7	publicCTFGC
4	GO:0015914	phospholipid transport	0.000951648	ANO6/ATP10B/ATP8B1/P2RX7/PITPNB	publicCTFGC
4	GO:0097035	regulation of membrane lipid distribution	0.000961242	ANO6/ATP10B/ATP8B1/P2RX7	publicCTFGC
7	GO:1902287	semaphorin-plexin signaling pathway involved in axon guidance	2.91E-06	PLXNA1/PLXNA3/PLXNA4/PLXND1	publicCTFollicle
7	GO:1902285	semaphorin-plexin signaling pathway involved in neuron projection guidance	4.17E-06	PLXNA1/PLXNA3/PLXNA4/PLXND1	publicCTFollicle
7	GO:0051453	regulation of intracellular pH	0.000131081	ATP6V1D/CFTR/SLC4A3/SLC4A8/SLC9A3/SLC9A5	publicCTFollicle
7	GO:0030641	regulation of cellular pH	0.000200738	ATP6V1D/CFTR/SLC4A3/SLC4A8/SLC9A3/SLC9A5	publicCTFollicle
7	GO:0048588	developmental cell growth	0.000293114	ABL1/EIF2AK4/GDF9/KMT2D/NLGN3/PLXNA1/PLXNA3/PLXNA4/TNN	publicCTFollicle
7	GO:0006885	regulation of pH	0.000329943	ATP6V1D/CFTR/SLC4A3/SLC4A8/SLC9A3/SLC9A5	publicCTFollicle
7	GO:0071526	semaphorin-plexin signaling pathway	0.000441603	PLXNA1/PLXNA3/PLXNA4/PLXND1	publicCTFollicle
7	GO:0098656	anion transmembrane transport	0.000562972	CASR/CFTR/PCYOX1/SLC15A4/SLC43A2/SLC4A3/SLC4A8/SLC6A6/SLC9A3/SLC9A5	publicCTFollicle
7	GO:0030004	cellular monovalent inorganic cation homeostasis	0.000567657	ATP6V1D/CFTR/SLC4A3/SLC4A8/SLC9A3/SLC9A5	publicCTFollicle
7	GO:1990138	neuron projection extension	0.000926629	ABL1/EIF2AK4/NLGN3/PLXNA1/PLXNA3/PLXNA4/TNN	publicCTFollicle
7	GO:0060560	developmental growth involved in morphogenesis	0.001323509	ABL1/BIN3/EIF2AK4/NLGN3/PLXNA1/PLXNA3/PLXNA4/TNN	publicCTFollicle
11	GO:0060041	retina development in camera-type eye	1.30E-05	DCX/IGFN1/LRP5/LRP6/MYH10/PTPRM/TTLL5/TUB/VSX2/ZHX2	publicCTOocyte
11	GO:0007018	microtubule-based movement	0.000142522	DNAH10/DNAH5/HTT/IFT57/KIF14/KIF16B/ KIF6/KLC2/MAP1B/RFX3/STARD9/SYNE2/TRIP11/TUB	publicCTOocyte
11	GO:0016579	protein deubiquitination	0.000614724	ACTB/ADRM1/ASXL2/BECN1/BIRC2/EP300/MBD5/OGT/SHMT2/USP54/VCP	publicCTOocyte
11	GO:0043010	camera-type eye development	0.000668165	DCX/IGFN1/KDM5B/LRP5/LRP6/MYH10/PTPRM/TTLL5/TUB/VSX2/WDPCP/ZHX2	publicCTOocyte
11	GO:0050808	synapse organization	0.000731785	ACTB/ACTN1/CNTN5/CTNND2/IGFN1/LRRTM3/MAP1B/ MYH10/NLGN3/NLGN4X/PCDHB13/SYNGAP1/SYNPO/VCP	publicCTOocyte
11	GO:0060042	retina morphogenesis in camera-type eye	0.00073938	IGFN1/LRP5/LRP6/PTPRM/ZHX2	publicCTOocyte
11	GO:0001654	eye development	0.000740424	DCX/IGFN1/KDM5B/LRP5/LRP6/MYH10/PTPRM/TTLL5/TUB/VSX2/WDPCP/ZDHHC16/ZHX2	publicCTOocyte
11	GO:0043467	regulation of generation of precursor metabolites and energy	0.000740886	EP300/INPP5K/NUP93/OGT/PFKFB1/SHMT2/SLC4A4/VCP	publicCTOocyte
11	GO:1903578	regulation of ATP metabolic process	0.000758063	EP300/NUP93/OGT/PFKFB1/SHMT2/SLC4A4/VCP	publicCTOocyte
11	GO:0016266	O-glycan processing	0.000797676	B3GNT2/MUC16/MUC17/MUC20/MUC4	publicCTOocyte

11	GO:0150063	visual system development	0.000814398	DCX/IGFN1/KDM5B/LRP5/LRP6/MYH10/PTPRM/TTLL5/TUB/VSX2/WDPCP/ZDHHC16/ZHX2	publicCTOocyte
11	GO:0048880	sensory system development	0.000936958	DCX/IGFN1/KDM5B/LRP5/LRP6/MYH10/PTPRM/TTLL5/TUB/VSX2/WDPCP/ZDHHC16/ZHX2	publicCTOocyte
11	GO:0070646	protein modification by small protein removal	0.000990848	ACTB/ADRM1/ASXL2/BECN1/BIRC2/EP300/MBD5/OGT/SHMT2/USP54/VCP	publicCTOocyte
12	GO:0061640	cytoskeleton-dependent cytokinesis	0.000116868	BIN3/MYH10/PRC1/SPAST/STAMBP/USP8/ZFYVE26	publicCTEmbryo
12	GO:0000281	mitotic cytokinesis	0.000138804	MYH10/PRC1/SPAST/STAMBP/USP8/ZFYVE26	publicCTEmbryo
13	GO:0007018	microtubule-based movement	3.29E-05	COPG1/COPG2/DNAH10/DYNC2H1/HTT/IFT57/KIF24/KIF6/ KLC2/NPHP4/RFX3/STARD9/SYNE2/TTLL9/TUB/WASF1	publicCTEmbryo
13	GO:0070593	dendrite self-avoidance	4.76E-05	DSCAM/DSCAML1/ROBO3/ROBO4	publicCTEmbryo
13	GO:0006814	sodium ion transport	9.06E-05	ANO6/ATP1A3/CNTN1/P2RX7/PTPN3/SCN1A/ SCN5A/SIK1/SLC24A1/SLC6A17/SLC6A3/SLC6A6	publicCTEmbryo
13	GO:0007411	axon guidance	0.000108577	B3GNT2/DSCAM/DSCAML1/EPHB1/EVL/GPC1/LAMA5/ PLXNA3/PLXNA4/PTPRM/RANBP9/ROBO3/ROBO4	publicCTEmbryo
13	GO:0097485	neuron projection guidance	0.000112453	B3GNT2/DSCAM/DSCAML1/EPHB1/EVL/GPC1/LAMA5/ PLXNA3/PLXNA4/PTPRM/RANBP9/ROBO3/ROBO4	publicCTEmbryo
13	GO:0030705	cytoskeleton-dependent intracellular transport	0.000336647	COPG1/COPG2/DYNC2H1/HTT/IFT57/MAP6D1/MYO1H/SYNE2/TUB/WASF1	publicCTEmbryo
13	GO:0035725	sodium ion transmembrane transport	0.000519836	ANO6/ATP1A3/PTPN3/SCN1A/SCN5A/SLC24A1/SLC6A17/SLC6A3/SLC6A6	publicCTEmbryo
13	GO:0021602	cranial nerve morphogenesis	0.000542652	ADARB1/EPHB1/PLXNA3/PLXNA4	publicCTEmbryo
13	GO:0006869	lipid transport	0.000769485	ABCA4/ANO6/APOL6/ATP10B/CFTR/ESYT2/GDF9/GRAMD1C/ LBP/LRP6/P2RX7/PITPNB/SREBF2/TEX2	publicCTEmbryo
13	GO:0060041	retina development in camera-type eye	0.000865362	DCX/DSCAM/LRP5/LRP6/NPHP4/PTPRM/RPE65/TUB	publicCTEmbryo
13	GO:0099111	microtubule-based transport	0.000974378	COPG1/COPG2/DYNC2H1/HTT/IFT57/RFX3/SYNE2/TUB/WASF1	publicCTEmbryo
15	GO:0061912	selective autophagy	8.03E-05	ATG2A/BAG3/BECN1/RB1CC1/SPTLC2/TSC2	publicCTEmbryo
15	GO:0051383	kinetochore organization	0.00011557	CENPN/CENPW/SENP6/SMC4	publicCTEmbryo
1	GO:0007409	axonogenesis	2.86E-06	CHL1/DSCAM/EPHB6/GLI2/ITGB1/L1CAM/LAMA1/LAMB2/LMX1A/MARK2/ MYPN/NFIB/NTNG1/PHOX2B/PLXND1/PTPRZ1/SHANK3/SLITRK2/TNN/TUBB3/WNT5A	ASDFGC
1	GO:0098742	cell-cell adhesion via plasma-membrane adhesion molecules	6.18E-06	CDH23/CDHR5/DSCAM/IGSF9B/ITGAL/ITGB1/L1CAM/MYPN/ NTNG1/PCDH1/PCDHB13/PCDHB16/SELP/SLITRK2/TRO	ASDFGC
1	GO:0050808	synapse organization	7.80E-06	DSCAM/GJA10/IGSF9B/L1CAM/LAMB2/LMX1A/LRRTM3/MECP2/NLGN2/ NTNG1/PCDHB13/PCDHB16/PLXND1/SHANK3/SLC6A1/SLC8A3/SLITRK2/VCP/WNT5A	ASDFGC
1	GO:0007156	homophilic cell adhesion via plasma membrane adhesion molecules	1.92E-05	CDH23/CDHR5/DSCAM/IGSF9B/ITGB1/L1CAM/MYPN/PCDH1/PCDHB13/PCDHB16/TRO	ASDFGC
1	GO:0007411	axon guidance	3.59E-05	CHL1/DSCAM/EPHB6/GLI2/L1CAM/LAMA1/LAMB2/ LMX1A/MYPN/NFIB/PLXND1/SHANK3/TUBB3/WNT5A	ASDFGC
1	GO:0097485	neuron projection guidance	3.73E-05	CHL1/DSCAM/EPHB6/GLI2/L1CAM/LAMA1/LAMB2/ LMX1A/MYPN/NFIB/PLXND1/SHANK3/TUBB3/WNT5A	ASDFGC
1	GO:0007416	synapse assembly	4.44E-05	DSCAM/GJA10/LRRTM3/MECP2/NLGN2/ PCDHB13/PCDHB16/PLXND1/SHANK3/SLITRK2/WNT5A	ASDFGC
1	GO:0048880	sensory system development	0.000102423	DCX/DSCAM/LAMA1/LAMB2/LRP5/MFRP/OLFM3/PBX2/ PHOX2B/PROM1/SPRED2/TTLL5/UNC45B/VSX2/WNT5A/ZDHHC16	ASDFGC
1	GO:0060041	retina development in camera-type eye	0.000213329	DCX/DSCAM/LAMA1/LAMB2/LRP5/MFRP/PROM1/TTLL5/VSX2	ASDFGC
1	GO:0001654	eye development	0.000255791	DCX/DSCAM/LAMA1/LAMB2/LRP5/MFRP/OLFM3/PBX2/ PROM1/SPRED2/TTLL5/UNC45B/VSX2/WNT5A/ZDHHC16	ASDFGC
1	GO:0150063	visual system development	0.000285635	DCX/DSCAM/LAMA1/LAMB2/LRP5/MFRP/OLFM3/PBX2/ PROM1/SPRED2/TTLL5/UNC45B/VSX2/WNT5A/ZDHHC16	ASDFGC
1	GO:0030902	hindbrain development	0.000285772	CNTN1/GART/GLI2/ITGB1/LMX1A/MECP2/NFIB/PHOX2B/RERE	ASDFGC
1	GO:0022612	gland morphogenesis	0.00029268	AR/ESRP2/GLI2/LAMA1/LRP5/NFIB/PLXND1/WNT5A	ASDFGC
1	GO:0017144	drug metabolic process	0.00038503	CYP1A2/CYP4F12/NOS1/NR1I2/NT5C2	ASDFGC
1	GO:0021537	telencephalon development	0.000858663	ALK/GART/LMX1A/MCPH1/NFIB/PAX5/RTN4RL1/SHANK3/SLC8A3/WNT5A/ZDHHC16	ASDFGC
1	GO:0060603	mammary gland duct morphogenesis	0.000883162	AR/GLI2/LRP5/WNT5A	ASDFGC
1	GO:0098810	neurotransmitter reuptake	0.000883162	ITGB1/ITGB3/NOS1/SLC6A1	ASDFGC
1	GO:0042311	vasodilation	0.001115048	CASR/EDNRB/KCNMA1/NOS1	ASDFGC
1	GO:0042391	regulation of membrane potential	0.001117232	ATP1A3/GNA11/GRIA1/GRIK1/IGSF9B/KCND3/KCNMA1/ MECP2/NLGN2/PIEZO1/SCN4A/SHANK3/SLC4A4/SLC8A3/VCP	ASDFGC
1	GO:0007435	salivary gland morphogenesis	0.00124564	ESRP2/LAMA1/NFIB/PLXND1	ASDFGC
1	GO:0048754	branching morphogenesis of an epithelial tube	0.001277268	AR/CASR/ESRP2/GLI2/LAMA1/LRP5/PLXND1/WNT5A	ASDFGC
1	GO:0031589	cell-substrate adhesion	0.001306137	GCNT2/HAS2/ITGA2B/ITGAL/ITGB1/ITGB3/ L1CAM/LAMB2/MSLN/NTNG1/SNED1/TECTA/TNN	ASDFGC
1	GO:0021955	central nervous system neuron axonogenesis	0.001386564	EPHB6/GLI2/NFIB/PHOX2B	ASDFGC
1	GO:0046530	photoreceptor cell differentiation	0.001637877	DCX/DSCAM/MFRP/OLFM3/PROM1	ASDFGC
1	GO:0001763	morphogenesis of a branching structure	0.001745242	AR/CASR/ESRP2/GLI2/LAMA1/LRP5/PLXND1/RERE/WNT5A	ASDFGC
1	GO:0007431	salivary gland development	0.001875537	ESRP2/LAMA1/NFIB/PLXND1	ASDFGC
1	GO:0043010	camera-type eye development	0.002016292	DCX/DSCAM/LAMA1/LAMB2/LRP5/MFRP/PROM1/SPRED2/TTLL5/UNC45B/VSX2/WNT5A	ASDFGC
1	GO:0001504	neurotransmitter uptake	0.002472559	ITGB1/ITGB3/NOS1/SLC6A1	ASDFGC
4	GO:0051899	membrane depolarization	3.84E-06	ABL1/CACNA1A/CACNA1D/CACNA1E/PTPN3/SCN1A/SLC8A1	ASDFGC
4	GO:0021545	cranial nerve development	3.03E-05	ATP8B1/EPHB1/EPHB2/NAV2/PLXNA4	ASDFGC
4	GO:0048013	ephrin receptor signaling pathway	3.62E-05	ACTB/APH1A/ARPC1B/EPHB1/EPHB2/GRIN2B	ASDFGC
4	GO:0021602	cranial nerve morphogenesis	5.04E-05	ATP8B1/EPHB1/EPHB2/PLXNA4	ASDFGC
4	GO:0034329	cell junction assembly	6.33E-05	ABL1/ACTB/CD151/CNTN5/EIF4G1/EPHB1/EPHB2/ITGA2/MAP1B/MUSK/PRKCA/SLC9A1	ASDFGC
4	GO:0099111	microtubule-based transport	6.45E-05	COPG2/DNAH11/DNAH5/DTNBP1/DYNC2H1/MAP1B/SLC9A3R1/TMEM201	ASDFGC
4	GO:0030516	regulation of axon extension	6.68E-05	ABL1/DISC1/MAP1B/PLXNA4/SEMA5A/SIN3A	ASDFGC
4	GO:0086010	membrane depolarization during action potential	0.000133987	CACNA1D/PTPN3/SCN1A/SLC8A1	ASDFGC
4	GO:0061387	regulation of extent of cell growth	0.000141124	ABL1/DISC1/MAP1B/PLXNA4/SEMA5A/SIN3A	ASDFGC
4	GO:1990778	protein localization to cell periphery	0.000160174	ACTB/ADAM10/DPP6/EPHB2/PPP2R5A/RILPL2/SCRIB/SLC9A3R1/STX3/TTC7A	ASDFGC
4	GO:0072659	protein localization to plasma membrane	0.000182713	ACTB/DPP6/EPHB2/PPP2R5A/RILPL2/SCRIB/SLC9A3R1/STX3/TTC7A	ASDFGC
4	GO:0034765	regulation of ion transmembrane transport	0.000193759	ABL1/CACNA1A/CACNA1D/CACNA1E/DPP6/ EPHB2/GRIN2B/PTPN3/SCN1A/SLC8A1/SLC9A1/SLC9A3R1	ASDFGC
4	GO:0050807	regulation of synapse organization	0.000234112	ABL1/ADAM10/DISC1/EIF4G1/EPHB1/EPHB2/GRIN2B/MUSK	ASDFGC
4	GO:0050954	sensory perception of mechanical stimulus	0.000238563	ATP8B1/CACNA1D/CNTN5/ITGA2/NAV2/SCN1A/SLC9A3R1	ASDFGC
4	GO:0048675	axon extension	0.000247107	ABL1/DISC1/MAP1B/PLXNA4/SEMA5A/SIN3A	ASDFGC
4	GO:0021675	nerve development	0.000248095	ATP8B1/EPHB1/EPHB2/NAV2/PLXNA4	ASDFGC
4	GO:0050808	synapse organization	0.000268589	ABL1/ACTB/ADAM10/CNTN5/DISC1/EIF4G1/EPHB1/EPHB2/GRIN2B/MAP1B/MUSK	ASDFGC
4	GO:0008361	regulation of cell size	0.000314114	ABL1/DISC1/MAP1B/PLXNA4/SEMA5A/SIN3A/SLC9A3R1	ASDFGC
4	GO:0050803	regulation of synapse structure or activity	0.000321146	ABL1/ADAM10/DISC1/EIF4G1/EPHB1/EPHB2/GRIN2B/MUSK	ASDFGC
4	GO:0050770	regulation of axonogenesis	0.000370351	ABL1/DISC1/EPHB2/MAP1B/PLXNA4/SEMA5A/SIN3A	ASDFGC
4	GO:0050804	modulation of chemical synaptic transmission	0.000401152	ABL1/CACNA1A/CACNA1D/DISC1/DTNBP1/EPHB1/EPHB2/GRIN2B/MAP1B/STX3/USP46	ASDFGC
4	GO:0099177	regulation of trans-synaptic signaling	0.000408637	ABL1/CACNA1A/CACNA1D/DISC1/DTNBP1/EPHB1/EPHB2/GRIN2B/MAP1B/STX3/USP46	ASDFGC
4	GO:0032535	regulation of cellular component size	0.000413856	ABL1/ARPC1B/DISC1/IQGAP2/MAP1B/NEB/PLXNA4/SEMA5A/SIN3A/SLC9A3R1	ASDFGC
4	GO:0001558	regulation of cell growth	0.000843471	ABL1/ADAM10/DISC1/EIF4G1/MAP1B/PLXNA4/SEMA5A/SESN2/SIN3A/SLC9A1	ASDFGC
4	GO:1904062	regulation of cation transmembrane transport	0.000879962	ABL1/CACNA1D/DPP6/EPHB2/GRIN2B/PTPN3/SLC8A1/SLC9A1/SLC9A3R1	ASDFGC
4	GO:0071868	cellular response to monoamine stimulus	0.000957687	ABL1/DTNBP1/SIN3A/SLC9A1/SLC9A3R1	ASDFGC
4	GO:0071870	cellular response to catecholamine stimulus	0.000957687	ABL1/DTNBP1/SIN3A/SLC9A1/SLC9A3R1	ASDFGC
4	GO:0071867	response to monoamine	0.00113082	ABL1/DTNBP1/SIN3A/SLC9A1/SLC9A3R1	ASDFGC
4	GO:0071869	response to catecholamine	0.00113082	ABL1/DTNBP1/SIN3A/SLC9A1/SLC9A3R1	ASDFGC
4	GO:0042391	regulation of membrane potential	0.001260472	ABL1/CACNA1A/CACNA1D/CACNA1E/GRIN2B/PRDX3/PTPN3/SCN1A/SLC8A1/SLC9A1	ASDFGC
4	GO:0007018	microtubule-based movement	0.00127375	COPG2/DNAH10/DNAH11/DNAH5/DTNBP1/DYNC2H1/MAP1B/SLC9A3R1/TMEM201	ASDFGC
4	GO:0099072	regulation of postsynaptic membrane neurotransmitter receptor levels	0.001394161	ADAM10/SCRIB/STX3/USP46	ASDFGC
4	GO:0030307	positive regulation of cell growth	0.001401411	ADAM10/DISC1/EIF4G1/MAP1B/SEMA5A/SLC9A1	ASDFGC
4	GO:0050806	positive regulation of synaptic transmission	0.001443742	ABL1/CACNA1D/DTNBP1/EPHB2/GRIN2B/STX3	ASDFGC
4	GO:1990138	neuron projection extension	0.001443742	ABL1/DISC1/MAP1B/PLXNA4/SEMA5A/SIN3A	ASDFGC
4	GO:0099173	postsynapse organization	0.001487042	ACTB/ADAM10/EPHB1/EPHB2/GRIN2B/MUSK	ASDFGC
4	GO:0010769	regulation of cell morphogenesis involved in differentiation	0.001692012	ABL1/ADAM10/DISC1/EPHB2/MAP1B/PLXNA4/SEMA5A/SIN3A	ASDFGC
4	GO:1904375	regulation of protein localization to cell periphery	0.001855409	ACTB/ADAM10/EPHB2/PPP2R5A/STX3	ASDFGC
4	GO:0007416	synapse assembly	0.002031484	CNTN5/EIF4G1/EPHB1/EPHB2/MAP1B/MUSK	ASDFGC
4	GO:0007409	axonogenesis	0.002342808	ABL1/ACTB/DISC1/EPHB1/EPHB2/MAP1B/PLXNA4/PRKCA/SEMA5A/SIN3A	ASDFGC
4	GO:0042326	negative regulation of phosphorylation	0.002413913	ABL1/DTNBP1/EIF4G1/EPHB2/MAPK8IP1/PPP2R5A/PPP2R5D/PRDX3/SLC8A1/SLC9A3R1	ASDFGC
4	GO:0021953	central nervous system neuron differentiation	0.002513527	DISC1/DYNC2H1/EPHB1/EPHB2/PLXNA4/SIN3A	ASDFGC
4	GO:0016049	cell growth	0.002637711	ABL1/ADAM10/DISC1/EIF4G1/MAP1B/PLXNA4/SEMA5A/SESN2/SIN3A/SLC9A1	ASDFGC
4	GO:0021954	central nervous system neuron development	0.002774229	DISC1/EPHB1/EPHB2/PLXNA4	ASDFGC
4	GO:0022604	regulation of cell morphogenesis	0.003004367	ABL1/ADAM10/DISC1/EPHB2/MAP1B/MOV10/PLXNA4/SEMA5A/SIN3A/SLC9A3R1	ASDFGC
4	GO:0097061	dendritic spine organization	0.003027302	ADAM10/EPHB1/EPHB2/GRIN2B	ASDFGC
4	GO:0001508	action potential	0.003059304	CACNA1D/PTPN3/SCN1A/SLC8A1/SLC9A1	ASDFGC
4	GO:0033673	negative regulation of kinase activity	0.003066458	ABL1/DTNBP1/EPHB2/MAPK8IP1/PPP2R5A/PRDX3/SLC8A1	ASDFGC
4	GO:0030705	cytoskeleton-dependent intracellular transport	0.003076961	COPG2/DTNBP1/DYNC2H1/MAP1B/MYO1H/TMEM201	ASDFGC
4	GO:1901888	regulation of cell junction assembly	0.003076961	ABL1/EIF4G1/EPHB1/EPHB2/MUSK/SLC9A1	ASDFGC
4	GO:0070509	calcium ion import	0.003159562	CACNA1A/CACNA1D/CACNA1E/SLC8A1	ASDFGC
4	GO:0072384	organelle transport along microtubule	0.003159562	COPG2/DTNBP1/MAP1B/TMEM201	ASDFGC
4	GO:0050772	positive regulation of axonogenesis	0.003295713	DISC1/MAP1B/PLXNA4/SEMA5A	ASDFGC
4	GO:0060291	long-term synaptic potentiation	0.003435808	ABL1/EPHB2/GRIN2B/STX3	ASDFGC
4	GO:0002028	regulation of sodium ion transport	0.003579902	PTPN3/SLC8A1/SLC9A1/SLC9A3R1	ASDFGC
4	GO:0022898	regulation of transmembrane transporter activity	0.003604751	ACTB/CACNA1D/EPHB2/GRIN2B/PTPN3/SLC9A1/SLC9A3R1	ASDFGC
4	GO:0007015	actin filament organization	0.003861567	ABL1/ARPC1B/DIAPH3/IQGAP2/MICAL2/MYO1H/NEB/SEMA5A/SLC9A1	ASDFGC
4	GO:1903351	cellular response to dopamine	0.003880302	ABL1/DTNBP1/SIN3A/SLC9A3R1	ASDFGC
4	GO:1903350	response to dopamine	0.004036714	ABL1/DTNBP1/SIN3A/SLC9A3R1	ASDFGC
4	GO:0106027	neuron projection organization	0.004197339	ADAM10/EPHB1/EPHB2/GRIN2B	ASDFGC
4	GO:0007605	sensory perception of sound	0.004616434	ATP8B1/CACNA1D/CNTN5/NAV2/SLC9A3R1	ASDFGC
4	GO:0051668	localization within membrane	0.004705009	MUSK/REEP2/RILPL2/SCRIB	ASDFGC
4	GO:0030048	actin filament-based movement	0.004745736	CACNA1D/MYO1H/NEB/SCN1A/SLC9A1	ASDFGC
4	GO:0032409	regulation of transporter activity	0.004804899	ACTB/CACNA1D/EPHB2/GRIN2B/PTPN3/SLC9A1/SLC9A3R1	ASDFGC

4	GO:0007163	establishment or maintenance of cell polarity	0.004895281	ACTB/EPHB1/MAP1B/SCRIB/SLC9A1/SLC9A3R1	ASDFGC
4	GO:0099003	vesicle-mediated transport in synapse	0.004895281	ACTB/CACNA1D/DTNBP1/SCRIB/STX3/USP46	ASDFGC
4	GO:0051348	negative regulation of transferase activity	0.005265274	ABL1/DTNBP1/EPHB2/MAPK8IP1/PPP2R5A/PRDX3/SLC8A1	ASDFGC
4	GO:0010770	positive regulation of cell morphogenesis involved in differentiation	0.005288773	ABL1/DISC1/MAP1B/PLXNA4/SEMA5A	ASDFGC
4	GO:0043624	cellular protein complex disassembly	0.005337504	GSPT1/MAP1B/MICAL2/MRPL48/MRPL54/SEMA5A	ASDFGC
4	GO:0007044	cell-substrate junction assembly	0.005443994	ABL1/CD151/ITGA2/SLC9A1	ASDFGC
4	GO:0010970	transport along microtubule	0.005874426	COPG2/DTNBP1/DYNC2H1/MAP1B/TMEM201	ASDFGC
4	GO:0060996	dendritic spine development	0.006046542	ADAM10/DISC1/EPHB1/EPHB2	ASDFGC
4	GO:0150115	cell-substrate junction organization	0.006046542	ABL1/CD151/ITGA2/SLC9A1	ASDFGC
4	GO:1903076	regulation of protein localization to plasma membrane	0.006046542	ACTB/EPHB2/PPP2R5A/STX3	ASDFGC
4	GO:1902414	protein localization to cell junction	0.00669194	ACTB/ADAM10/SCRIB/STX3	ASDFGC
4	GO:0031667	response to nutrient levels	0.006700737	ADSL/CAD/EIF4G1/ITGA2/MAP1B/PPM1D/SESN2/SLC8A1/SREBF2	ASDFGC
4	GO:0060560	developmental growth involved in morphogenesis	0.006840152	ABL1/DISC1/MAP1B/PLXNA4/SEMA5A/SIN3A	ASDFGC
4	GO:0030900	forebrain development	0.006867424	DISC1/DNAH5/DYNC2H1/EPHB2/PLXNA4/SEMA5A/SIN3A/SLC8A1	ASDFGC
4	GO:0019233	sensory perception of pain	0.006916813	EPHB1/ITGA2/SCN1A/SLC9A1	ASDFGC
4	GO:0070588	calcium ion transmembrane transport	0.006963196	ABL1/CACNA1A/CACNA1D/CACNA1E/GRIN2B/SLC8A1/SLC9A1	ASDFGC
4	GO:0046777	protein autophosphorylation	0.00697789	ABL1/CAD/EPHB1/MUSK/PPP2R5A/PPP2R5D	ASDFGC
4	GO:0048588	developmental cell growth	0.00697789	ABL1/DISC1/MAP1B/PLXNA4/SEMA5A/SIN3A	ASDFGC
4	GO:0051963	regulation of synapse assembly	0.007146631	EIF4G1/EPHB1/EPHB2/MUSK	ASDFGC
4	GO:0007229	integrin-mediated signaling pathway	0.007381441	ABL1/ADAM10/ITGA11/ITGA2	ASDFGC
4	GO:0042493	response to drug	0.007497861	ABCC4/ADAM10/ATP8B1/CAD/HADHA/ITGA2/MAP1B/SLC8A1	ASDFGC
4	GO:0035725	sodium ion transmembrane transport	0.008092527	PTPN3/SCN1A/SLC8A1/SLC9A1/SLC9A3R1	ASDFGC
4	GO:0016358	dendrite development	0.008468026	ADAM10/DISC1/DTNBP1/EPHB1/EPHB2/MAP1B	ASDFGC
4	GO:0016311	dephosphorylation	0.008564695	CCDC8/INPP4B/ITGA2/NUDT16L1/PPM1D/PPP2R5A/PPP2R5D/PTPN3/PTPRU	ASDFGC
4	GO:0032984	protein-containing complex disassembly	0.009326605	GSPT1/MAP1B/MICAL2/MRPL48/MRPL54/SEMA5A/SMARCE1	ASDFGC
4	GO:0018107	peptidyl-threonine phosphorylation	0.009726425	CAD/EIF4G1/PPP2R5D/PRKCA	ASDFGC
5	GO:0050808	synapse organization	7.75E-08	ACTB/ADNP/DSCAM/EIF4G1/EPHB2/GJA10/LMX1A/LRRTM3/MAP1B/ MYH10/NFIA/PCDHB16/RELN/SHANK2/SLC6A1/SLC8A3/SLITRK2/WNT5A	ASDFollicle
5	GO:0034329	cell junction assembly	8.02E-08	ACTB/ADNP/DSCAM/EIF4G1/EPB41L3/EPHB2/GJA10/ITGA2/LRRTM3/ MACF1/MAP1B/PCDHB16/PKP4/PTPRJ/SHANK2/SLITRK2/TNS1/WNT5A	ASDFollicle
5	GO:0007416	synapse assembly	9.06E-07	ADNP/DSCAM/EIF4G1/EPHB2/GJA10/LRRTM3/ MAP1B/PCDHB16/SHANK2/SLITRK2/WNT5A	ASDFollicle
5	GO:0007409	axonogenesis	3.21E-05	ACTB/ADNP/ARX/DSCAM/EPHB2/GLI2/LMX1A/MACF1/ MAP1B/MYH10/PHOX2B/RELN/SLITRK2/TBR1/WNT5A	ASDFollicle
5	GO:0045666	positive regulation of neuron differentiation	4.21E-05	ADNP/ALK/CNTN1/DSCAM/EIF4G1/GLI2/MACF1/ MAP1B/PHOX2B/RELN/SHANK2/TCF4/WNT5A	ASDFollicle
5	GO:0021537	telencephalon development	0.000124009	ALK/ARX/EPHB2/LMX1A/MYH10/RELN/RTN4RL1/SLC8A3/TBR1/WNT5A	ASDFollicle
5	GO:0050769	positive regulation of neurogenesis	0.000131387	ADNP/ALK/CNTN1/DSCAM/EIF4G1/EPHB2/GLI2/ MACF1/MAP1B/PHOX2B/RELN/SHANK2/TCF4/WNT5A	ASDFollicle
5	GO:0001843	neural tube closure	0.000173482	GLMN/IFT57/MED12/SETD2/TSC2/WNT5A	ASDFollicle
5	GO:0060606	tube closure	0.000184216	GLMN/IFT57/MED12/SETD2/TSC2/WNT5A	ASDFollicle
5	GO:0030900	forebrain development	0.000229565	ALK/ARX/EPHB2/GLI2/LMX1A/MYH10/RELN/RTN4RL1/SETD2/SLC8A3/TBR1/WNT5A	ASDFollicle
5	GO:0007160	cell-matrix adhesion	0.000245442	BCAM/CASK/ITGA11/ITGA2/ITGA2B/MACF1/MSLN/MUC4/PTPRJ	ASDFollicle
5	GO:0014020	primary neural tube formation	0.000260126	GLMN/IFT57/MED12/SETD2/TSC2/WNT5A	ASDFollicle
5	GO:0001841	neural tube formation	0.000397356	GLMN/IFT57/MED12/SETD2/TSC2/WNT5A	ASDFollicle
5	GO:0051963	regulation of synapse assembly	0.000417862	ADNP/EIF4G1/EPHB2/LRRTM3/SLITRK2/WNT5A	ASDFollicle
5	GO:0031589	cell-substrate adhesion	0.000424624	BCAM/CASK/GCNT2/ITGA11/ITGA2/ITGA2B/KANK1/MACF1/MSLN/MUC4/PTPRJ	ASDFollicle
5	GO:0050890	cognition	0.000424663	ADNP/EPHB2/GRIA1/JAKMIP1/LMX1A/RELN/SHANK2/SLC6A1/SLC8A3/TBR1	ASDFollicle
5	GO:0016331	morphogenesis of embryonic epithelium	0.000443323	GLI2/GLMN/IFT57/MED12/SETD2/TSC2/WNT5A	ASDFollicle
5	GO:1901888	regulation of cell junction assembly	0.000469588	ADNP/EIF4G1/EPHB2/LRRTM3/MACF1/PTPRJ/SLITRK2/WNT5A	ASDFollicle
5	GO:0045773	positive regulation of axon extension	0.000597099	ADNP/DSCAM/MACF1/MAP1B	ASDFollicle
5	GO:0007611	learning or memory	0.000599426	ADNP/EPHB2/GRIA1/LMX1A/RELN/SHANK2/SLC6A1/SLC8A3/TBR1	ASDFollicle
5	GO:0021915	neural tube development	0.000699411	GLI2/GLMN/IFT57/MED12/SETD2/TSC2/WNT5A	ASDFollicle
5	GO:0007613	memory	0.000801393	ADNP/GRIA1/LMX1A/RELN/SLC6A1/SLC8A3	ASDFollicle
5	GO:0060562	epithelial tube morphogenesis	0.000861867	GLI2/GLMN/IFT57/KDM5B/MED12/MICAL2/SETD2/TBR1/TSC2/WNT5A	ASDFollicle
5	GO:0021954	central nervous system neuron development	0.000873309	ARX/EPHB2/GLI2/PHOX2B/WNT5A	ASDFollicle
5	GO:0001838	embryonic epithelial tube formation	0.000949651	GLMN/IFT57/MED12/SETD2/TSC2/WNT5A	ASDFollicle
5	GO:0099054	presynapse assembly	0.001061079	EIF4G1/LRRTM3/SLITRK2/WNT5A	ASDFollicle
5	GO:0007411	axon guidance	0.001118417	ARX/DSCAM/EPHB2/GLI2/LMX1A/MYH10/RELN/TBR1/WNT5A	ASDFollicle
5	GO:0097485	neuron projection guidance	0.001146127	ARX/DSCAM/EPHB2/GLI2/LMX1A/MYH10/RELN/TBR1/WNT5A	ASDFollicle
5	GO:0010976	positive regulation of neuron projection development	0.00129314	ADNP/ALK/CNTN1/DSCAM/MACF1/MAP1B/RELN/SHANK2/WNT5A	ASDFollicle
5	GO:0072175	epithelial tube formation	0.001308735	GLMN/IFT57/MED12/SETD2/TSC2/WNT5A	ASDFollicle
5	GO:0099172	presynapse organization	0.001321207	EIF4G1/LRRTM3/SLITRK2/WNT5A	ASDFollicle
5	GO:0050770	regulation of axonogenesis	0.001508094	ADNP/DSCAM/EPHB2/MACF1/MAP1B/TBR1/WNT5A	ASDFollicle
5	GO:0021953	central nervous system neuron differentiation	0.001807447	ARX/EPHB2/GLI2/LMX1A/PHOX2B/TBR1/WNT5A	ASDFollicle
5	GO:0016358	dendrite development	0.001830746	ALK/ARID1B/DSCAM/DTNBP1/EPHB2/MAP1B/RELN/SHANK2	ASDFollicle
5	GO:0030516	regulation of axon extension	0.001851363	ADNP/DSCAM/MACF1/MAP1B/WNT5A	ASDFollicle
5	GO:0007018	microtubule-based movement	0.001899522	AP3D1/BAG3/COPG2/DNAH2/DTNBP1/GAS2L2/IFT57/MAP1B/MAP4/STARD9	ASDFollicle
5	GO:0007612	learning	0.002031362	EPHB2/RELN/SHANK2/SLC6A1/SLC8A3/TBR1	ASDFollicle
5	GO:0010769	regulation of cell morphogenesis involved in differentiation	0.002040798	ADNP/DSCAM/EPHB2/KANK1/MACF1/MAP1B/RELN/TBR1/WNT5A	ASDFollicle
5	GO:0140115	export across plasma membrane	0.002364006	ATP1A3/KCND3/SLC4A4/SLC8A3	ASDFollicle
5	GO:0035148	tube formation	0.002408319	GLMN/IFT57/MED12/SETD2/TSC2/WNT5A	ASDFollicle
5	GO:1901890	positive regulation of cell junction assembly	0.002408634	ADNP/EPHB2/LRRTM3/PTPRJ/SLITRK2	ASDFollicle
5	GO:0001947	heart looping	0.002655368	IFT57/MICAL2/TBR1/WNT5A	ASDFollicle
5	GO:1900449	regulation of glutamate receptor signaling pathway	0.002655368	EPHB2/GRIA1/RELN/SHANK2	ASDFollicle
5	GO:0050804	modulation of chemical synaptic transmission	0.002794574	ADNP/CACNA1A/CASK/DTNBP1/EPHB2/GRIA1/MAP1B/RELN/SHANK2/SLC6A1/SLC8A3	ASDFollicle
5	GO:0099177	regulation of trans-synaptic signaling	0.002841905	ADNP/CACNA1A/CASK/DTNBP1/EPHB2/GRIA1/MAP1B/RELN/SHANK2/SLC6A1/SLC8A3	ASDFollicle
5	GO:0030902	hindbrain development	0.002927542	CNTN1/GLI2/LMX1A/MYH10/PHOX2B/TBR1	ASDFollicle
5	GO:0031346	positive regulation of cell projection organization	0.003107299	ADNP/ALK/CNTN1/DSCAM/ITGA2/MACF1/MAP1B/RELN/SHANK2/WNT5A	ASDFollicle
5	GO:0050918	positive chemotaxis	0.003137182	ITGA2/PTPRJ/TSC2/WNT5A	ASDFollicle
5	GO:0051965	positive regulation of synapse assembly	0.003137182	ADNP/EPHB2/LRRTM3/SLITRK2	ASDFollicle
5	GO:0061387	regulation of extent of cell growth	0.003329501	ADNP/DSCAM/MACF1/MAP1B/WNT5A	ASDFollicle
5	GO:0010970	transport along microtubule	0.003420715	AP3D1/BAG3/COPG2/DTNBP1/IFT57/MAP1B	ASDFollicle
5	GO:0061371	determination of heart left/right asymmetry	0.003489571	IFT57/MICAL2/TBR1/WNT5A	ASDFollicle
5	GO:0003143	embryonic heart tube morphogenesis	0.003675457	IFT57/MICAL2/TBR1/WNT5A	ASDFollicle
5	GO:0072659	protein localization to plasma membrane	0.004032595	ACTB/DPP6/EPB41L3/EPHB2/MACF1/PPP2R5A/RILPL2/TTC7A	ASDFollicle
5	GO:0050805	negative regulation of synaptic transmission	0.004706171	ADNP/GRIA1/SHANK2/SLC6A1	ASDFollicle
5	GO:0050807	regulation of synapse organization	0.00479745	ADNP/EIF4G1/EPHB2/LRRTM3/RELN/SLITRK2/WNT5A	ASDFollicle
5	GO:0099173	postsynapse organization	0.004854533	ACTB/EPHB2/MYH10/RELN/SHANK2/WNT5A	ASDFollicle
5	GO:0010469	regulation of signaling receptor activity	0.004991265	EPHB2/GRIA1/MUC4/RELN/SHANK2/WFIKKN2	ASDFollicle
5	GO:0048675	axon extension	0.005150689	ADNP/DSCAM/MACF1/MAP1B/WNT5A	ASDFollicle
5	GO:0060560	developmental growth involved in morphogenesis	0.005638684	ADNP/DSCAM/KDM5B/MACF1/MAP1B/MED12/WNT5A	ASDFollicle
5	GO:0099601	regulation of neurotransmitter receptor activity	0.005658646	EPHB2/GRIA1/RELN/SHANK2	ASDFollicle
5	GO:0022604	regulation of cell morphogenesis	0.005658922	ADNP/DSCAM/EPB41L3/EPHB2/KANK1/MACF1/MAP1B/MYH10/RELN/TBR1/WNT5A	ASDFollicle
5	GO:0035050	embryonic heart tube development	0.005915384	IFT57/MICAL2/TBR1/WNT5A	ASDFollicle
5	GO:0050803	regulation of synapse structure or activity	0.006166506	ADNP/EIF4G1/EPHB2/LRRTM3/RELN/SLITRK2/WNT5A	ASDFollicle
5	GO:0048562	embryonic organ morphogenesis	0.006173861	EPHB2/GLI2/IFT57/MED12/MICAL2/SETD2/TBR1/WNT5A	ASDFollicle
5	GO:0006814	sodium ion transport	0.006304041	ATP1A3/CNTN1/SIK1/SLC4A4/SLC6A1/SLC6A8/SLC8A3	ASDFollicle
5	GO:0070509	calcium ion import	0.007315673	CACNA1A/CACNA1E/CASK/SLC8A3	ASDFollicle
5	GO:0072384	organelle transport along microtubule	0.007315673	AP3D1/COPG2/DTNBP1/MAP1B	ASDFollicle
5	GO:0099111	microtubule-based transport	0.007583986	AP3D1/BAG3/COPG2/DTNBP1/IFT57/MAP1B	ASDFollicle
5	GO:0050772	positive regulation of axonogenesis	0.007619707	ADNP/DSCAM/MACF1/MAP1B	ASDFollicle
5	GO:2000106	regulation of leukocyte apoptotic process	0.007619707	GIMAP8/HCAR2/TSC22D3/WNT5A	ASDFollicle
5	GO:0048167	regulation of synaptic plasticity	0.007773047	EPHB2/GRIA1/MAP1B/RELN/SHANK2/SLC8A3	ASDFollicle
5	GO:0060291	long-term synaptic potentiation	0.007931946	EPHB2/RELN/SHANK2/SLC8A3	ASDFollicle
5	GO:0032465	regulation of cytokinesis	0.008918765	KLHL21/PKP4/SETD2/ZFYVE26	ASDFollicle
5	GO:0043044	ATP-dependent chromatin remodeling	0.008918765	ACTB/ARID1B/SMARCE1/SRCAP	ASDFollicle
5	GO:0051056	regulation of small GTPase mediated signal transduction	0.00908729	CGNL1/CYTH4/EPHB2/KANK1/RALGAPA1/RELN/STARD8/TSC2	ASDFollicle
8	GO:0007063	regulation of sister chromatid cohesion	2.51E-05	ATRX/CTNNB1/SMC5/TNKS	ASDFollicle
8	GO:0007064	mitotic sister chromatid cohesion	5.01E-05	ATRX/POGZ/SMC5/TNKS	ASDFollicle
8	GO:0007062	sister chromatid cohesion	0.000144111	ATRX/CTNNB1/POGZ/SMC5/TNKS	ASDFollicle
8	GO:0140014	mitotic nuclear division	0.000480288	ATRX/MKI67/NCAPH/POGZ/PRKCA/SMC5/SPAST/TNKS/TUBG1	ASDFollicle
10	GO:0007409	axonogenesis	9.49E-07	ABL1/ADNP/DSCAM/EPHA7/GLI2/L1CAM/LAMA1/LAMA3/LMX1A/MACF1/ NFIB/NTNG1/PHOX2B/PLXNA4/PTEN/RELN/SHANK3/SLITRK2/SPAST/TBR1/WNT5A	ASDOocyte
10	GO:0050808	synapse organization	2.87E-06	ABL1/ADAM10/ADNP/DOCK10/DSCAM/EIF4G1/EPHA7/IGSF9B/L1CAM/ LMX1A/NFIA/NTNG1/PTEN/RELN/SHANK3/SLC6A1/SLC8A3/SLITRK2/WNT5A	ASDOocyte
10	GO:0033555	multicellular organismal response to stress	5.30E-06	DPP4/EDNRB/EIF4G1/NOS1/PENK/PTEN/RELN/USP46	ASDOocyte
10	GO:0031646	positive regulation of nervous system process	1.79E-05	DICER1/IGSF9B/ITGA2/PTEN/RELN/SHANK3/TENM4	ASDOocyte
10	GO:0031644	regulation of nervous system process	1.95E-05	DICER1/EDNRB/IGSF9B/ITGA2/PTEN/RELN/SHANK3/SLC8A3/SLC9A1/TENM4	ASDOocyte
10	GO:0008361	regulation of cell size	2.03E-05	ABL1/ADNP/DSCAM/EPHA7/L1CAM/MACF1/PLXNA4/PTEN/RB1CC1/SHANK3/WNT5A	ASDOocyte
10	GO:0032535	regulation of cellular component size	3.24E-05	ABL1/ADNP/CDHR5/DSCAM/EPHA7/L1CAM/MACF1/MAGEL2/ NCKAP1/NCKAP1L/PLEKHG2/PLXNA4/PTEN/RB1CC1/SHANK3/WNT5A	ASDOocyte
10	GO:0010769	regulation of cell morphogenesis involved in differentiation	4.39E-05	ABL1/ADAM10/ADNP/DSCAM/EPHA7/HAS2/ L1CAM/MACF1/PLXNA4/PTEN/RELN/SHANK3/TBR1/WNT5A	ASDOocyte
10	GO:0050769	positive regulation of neurogenesis	4.94E-05	ADNP/ALK/DICER1/DSCAM/EIF4G1/GLI2/L1CAM/LRP2/MACF1/ PHOX2B/PLXNA4/PTEN/RELN/SHANK3/SPAG9/TCF4/TENM4/WNT5A	ASDOocyte

10	GO:0007411	axon guidance	7.18E-05	DSCAM/EPHA7/GLI2/L1CAM/LAMA1/LAMA3/LMX1A/NFIB/PLXNA4/RELN/SHANK3/TBR1/WNT5A	ASDOocyte
10	GO:0097485	neuron projection guidance	7.44E-05	DSCAM/EPHA7/GLI2/L1CAM/LAMA1/LAMA3/LMX1A/NFIB/PLXNA4/RELN/SHANK3/TBR1/WNT5A	ASDOocyte
10	GO:0061387	regulation of extent of cell growth	8.49E-05	ABL1/ADNP/DSCAM/EPHA7/L1CAM/MACF1/PLXNA4/WNT5A	ASDOocyte
10	GO:0021955	central nervous system neuron axonogenesis	8.56E-05	GLI2/NFIB/PHOX2B/PLXNA4/PTEN	ASDOocyte
10	GO:0045666	positive regulation of neuron differentiation	0.000107704	ADNP/ALK/DSCAM/EIF4G1/GLI2/L1CAM/MACF1/PHOX2B/PLXNA4/PTEN/RELN/SHANK3/SPAG9/TCF4/WNT5A	ASDOocyte
10	GO:0060291	long-term synaptic potentiation	0.000118488	ABL1/PTEN/RELN/SHANK3/SLC24A1/SLC8A3/STX3	ASDOocyte
10	GO:0021537	telencephalon development	0.000121442	ALK/LMX1A/NFIB/PAX5/PLXNA4/PTEN/RELN/RTN4RL1/SHANK3/SLC8A3/TBR1/WNT5A	ASDOocyte
10	GO:0050770	regulation of axonogenesis	0.000134682	ABL1/ADNP/DSCAM/EPHA7/L1CAM/MACF1/PLXNA4/PTEN/TBR1/WNT5A	ASDOocyte
10	GO:0030900	forebrain development	0.000147312	ALK/ATRX/GLI2/LMX1A/LRP2/NFIB/PAX5/PLXNA4/PTEN/RELN/RTN4RL1/SHANK3/SLC8A3/TBR1/WNT5A	ASDOocyte
10	GO:0007613	memory	0.000155186	ADNP/GRIA1/LMX1A/PTEN/RELN/SHANK3/SLC6A1/SLC8A3	ASDOocyte
10	GO:0030516	regulation of axon extension	0.000234074	ABL1/ADNP/DSCAM/L1CAM/MACF1/PLXNA4/WNT5A	ASDOocyte
10	GO:1901888	regulation of cell junction assembly	0.000242853	ABL1/ADNP/EIF4G1/EPHA7/MACF1/PTEN/PTPRJ/SLC9A1/SLITRK2/WNT5A	ASDOocyte
10	GO:0007044	cell-substrate junction assembly	0.000265526	ABL1/ITGA2/LAMA3/MACF1/PTEN/PTPRJ/SLC9A1	ASDOocyte
10	GO:0001754	eye photoreceptor cell differentiation	0.000280813	CEP290/DSCAM/MFRP/OLFM3/PROM1	ASDOocyte
10	GO:0140058	neuron projection arborization	0.000301559	LRP2/MACF1/NTNG1/WNT5A	ASDOocyte
10	GO:0050919	negative chemotaxis	0.00031103	DPP4/EPHA7/ITGB3/PLXNA4/WNT5A	ASDOocyte
10	GO:0150115	cell-substrate junction organization	0.000318994	ABL1/ITGA2/LAMA3/MACF1/PTEN/PTPRJ/SLC9A1	ASDOocyte
10	GO:0050805	negative regulation of synaptic transmission	0.000376872	ADNP/GRIA1/PTEN/SHANK3/SLC24A1/SLC6A1	ASDOocyte
10	GO:0034329	cell junction assembly	0.000448083	ABL1/ADNP/DSCAM/EIF4G1/EPHA7/ITGA2/LAMA3/MACF1/PKP4/PTEN/PTPRJ/SHANK3/SLC9A1/SLITRK2/WNT5A	ASDOocyte
10	GO:0007229	integrin-mediated signaling pathway	0.000451781	ABL1/ADAM10/BCAR1/ITGA2/ITGA2B/ITGB3/LAMA3	ASDOocyte
10	GO:0042063	gliogenesis	0.000572081	ABL1/CERS2/DICER1/LRP2/NFIB/PENK/PHOX2B/PTEN/RELN/SCRIB/SLC8A3/TENM4	ASDOocyte
10	GO:0048638	regulation of developmental growth	0.000596008	ABL1/ADNP/AR/DSCAM/EPHA7/IL7/L1CAM/MACF1/NRK/PLXNA4/PTEN/RAI1/WNT5A	ASDOocyte
10	GO:0060292	long-term synaptic depression	0.000603963	GRIA1/PTEN/SHANK3/SLC24A1	ASDOocyte
10	GO:0021954	central nervous system neuron development	0.000608952	GLI2/NFIB/PHOX2B/PLXNA4/PTEN/WNT5A	ASDOocyte
10	GO:0048041	focal adhesion assembly	0.000649555	ABL1/ITGA2/MACF1/PTEN/PTPRJ/SLC9A1	ASDOocyte
10	GO:0022604	regulation of cell morphogenesis	0.000656989	ABL1/ADAM10/ADNP/DSCAM/EPHA7/HAS2/L1CAM/MACF1/NTNG1/PLXNA4/PTEN/RELN/RHOBTB2/SHANK3/TBR1/WNT5A	ASDOocyte
10	GO:0035148	tube formation	0.000663005	ABL1/CEP290/DICER1/LRP2/NFIB/SCRIB/TSC2/WNT5A	ASDOocyte
10	GO:0060603	mammary gland duct morphogenesis	0.000683436	AR/GLI2/SCRIB/WNT5A	ASDOocyte
10	GO:0050807	regulation of synapse organization	0.000704949	ABL1/ADAM10/ADNP/EIF4G1/EPHA7/PTEN/RELN/SHANK3/SLITRK2/WNT5A	ASDOocyte
10	GO:0007160	cell-matrix adhesion	0.000728972	ABL1/ITGA2/ITGA2B/ITGB3/L1CAM/MACF1/MSLN/PTEN/PTPRJ/SLC9A1	ASDOocyte
10	GO:0098657	import into cell	0.000728972	ITGB3/LRP2/NOS1/SCRIB/SLC24A1/SLC6A1/SLC7A2/SLC8A3/SLC9A1/USP46	ASDOocyte
10	GO:0021953	central nervous system neuron differentiation	0.000785901	GLI2/LMX1A/NFIB/PHOX2B/PLXNA4/PTEN/SHANK3/TBR1/WNT5A	ASDOocyte
10	GO:0030902	hindbrain development	0.000856924	ABL1/CEP290/GLI2/LMX1A/NFIB/PHOX2B/SCRIB/TBR1	ASDOocyte
10	GO:0010770	positive regulation of cell morphogenesis involved in differentiation	0.000893265	ABL1/ADNP/DSCAM/HAS2/L1CAM/MACF1/PLXNA4/RELN	ASDOocyte
10	GO:0001558	regulation of cell growth	0.000980261	ABL1/ADAM10/ADNP/BCAR1/DSCAM/EIF4G1/EPHA7/L1CAM/LMX1A/MACF1/PLXNA4/PTPRJ/SLC9A1/WNT5A	ASDOocyte
10	GO:0048675	axon extension	0.000980893	ABL1/ADNP/DSCAM/L1CAM/MACF1/PLXNA4/WNT5A	ASDOocyte
10	GO:0050803	regulation of synapse structure or activity	0.001008525	ABL1/ADAM10/ADNP/EIF4G1/EPHA7/PTEN/RELN/SHANK3/SLITRK2/WNT5A	ASDOocyte
10	GO:0010092	specification of animal organ identity	0.001076043	AR/LRP2/TBR1/WNT5A	ASDOocyte
10	GO:0060997	dendritic spine morphogenesis	0.001125034	ADAM10/DOCK10/PTEN/RELN/SHANK3	ASDOocyte
10	GO:0046530	photoreceptor cell differentiation	0.001209582	CEP290/DSCAM/MFRP/OLFM3/PROM1	ASDOocyte
10	GO:0051893	regulation of focal adhesion assembly	0.001209582	ABL1/MACF1/PTEN/PTPRJ/SLC9A1	ASDOocyte
10	GO:0090109	regulation of cell-substrate junction assembly	0.001209582	ABL1/MACF1/PTEN/PTPRJ/SLC9A1	ASDOocyte
10	GO:0048645	animal organ formation	0.001392656	AR/GLI2/LRP2/TBR1/WNT5A	ASDOocyte
10	GO:0001662	behavioral fear response	0.001458603	DPP4/EIF4G1/PENK/USP46	ASDOocyte
10	GO:0150116	regulation of cell-substrate junction organization	0.001491485	ABL1/MACF1/PTEN/PTPRJ/SLC9A1	ASDOocyte
10	GO:0016049	cell growth	0.001524901	ABL1/ADAM10/ADNP/BCAR1/DSCAM/EIF4G1/EPHA7/KMT2D/L1CAM/LMX1A/MACF1/PLXNA4/PTPRJ/SLC9A1/WNT5A	ASDOocyte
10	GO:0050918	positive chemotaxis	0.001595382	ITGA2/PTPRJ/SCRIB/TSC2/WNT5A	ASDOocyte
10	GO:0050806	positive regulation of synaptic transmission	0.00160298	ABL1/IGSF9B/PTEN/RELN/SHANK3/SLC24A1/SLC8A3/STX3	ASDOocyte
10	GO:0002209	behavioral defense response	0.001604738	DPP4/EIF4G1/PENK/USP46	ASDOocyte
10	GO:0045995	regulation of embryonic development	0.001612715	AR/LAMA1/LAMA3/NRK/POGLUT1/TENM4/WNT5A	ASDOocyte
10	GO:0099072	regulation of postsynaptic membrane neurotransmitter receptor levels	0.0017045	ADAM10/ITGB3/SCRIB/STX3/USP46	ASDOocyte
10	GO:0042596	fear response	0.001760751	DPP4/EIF4G1/PENK/USP46	ASDOocyte
10	GO:0060996	dendritic spine development	0.00189954	ADAM10/ARID1B/DOCK10/PTEN/RELN/SHANK3	ASDOocyte
10	GO:0046330	positive regulation of JNK cascade	0.001908014	MMP8/NRK/RB1CC1/SASH1/SH3RF3/SPAG9/WNT5A	ASDOocyte
10	GO:0045773	positive regulation of axon extension	0.002103758	ADNP/DSCAM/L1CAM/MACF1	ASDOocyte
10	GO:0031589	cell-substrate adhesion	0.002175422	ABL1/HAS2/ITGA2/ITGA2B/ITGB3/L1CAM/MACF1/MSLN/NTNG1/PTEN/PTPRJ/SLC9A1	ASDOocyte
10	GO:1902414	protein localization to cell junction	0.002200281	ADAM10/MAPK9/RELN/SCRIB/STX3/WNT5A	ASDOocyte
10	GO:1902667	regulation of axon guidance	0.002291423	DSCAM/PLXNA4/TBR1/WNT5A	ASDOocyte
10	GO:0099175	regulation of postsynapse organization	0.002419842	ADAM10/EPHA7/PTEN/RELN/SHANK3/WNT5A	ASDOocyte
10	GO:0007416	synapse assembly	0.002443758	ADNP/DSCAM/EIF4G1/EPHA7/PTEN/SHANK3/SLITRK2/WNT5A	ASDOocyte
10	GO:0060443	mammary gland morphogenesis	0.002490306	AR/GLI2/SCRIB/WNT5A	ASDOocyte
10	GO:0098815	modulation of excitatory postsynaptic potential	0.002490306	PTEN/RELN/SHANK3/SLC8A3	ASDOocyte
10	GO:0050920	regulation of chemotaxis	0.002651526	ADAM10/DPP4/DSCAM/ITGA2/NCKAP1L/PLXNA4/STX3/TBR1/WNT5A	ASDOocyte
10	GO:0017144	drug metabolic process	0.002700734	CYP1A2/NOS1/NR1I2/NT5C2	ASDOocyte
10	GO:0007369	gastrulation	0.002882256	COL12A1/ITGA2/ITGB3/LAMA3/MMP8/POGLUT1/TENM4/WNT5A	ASDOocyte
10	GO:0098742	cell-cell adhesion via plasma-membrane adhesion molecules	0.00289858	CDH23/CDHR5/DSCAM/IGSF9B/L1CAM/NTNG1/SELP/SLITRK2/TENM4/TRO	ASDOocyte
10	GO:0048167	regulation of synaptic plasticity	0.003073918	ABL1/GRIA1/PTEN/RELN/SHANK3/SLC24A1/SLC8A3/STX3	ASDOocyte
10	GO:0061001	regulation of dendritic spine morphogenesis	0.003157521	ADAM10/PTEN/RELN/SHANK3	ASDOocyte
10	GO:0099637	neurotransmitter receptor transport	0.003157521	ITGB3/SCRIB/STX3/USP46	ASDOocyte
10	GO:0016331	morphogenesis of embryonic epithelium	0.00316459	ABL1/AR/GLI2/LRP2/SCRIB/TSC2/WNT5A	ASDOocyte
10	GO:0048588	developmental cell growth	0.003330513	ABL1/ADNP/DSCAM/EPHA7/KMT2D/L1CAM/MACF1/PLXNA4/WNT5A	ASDOocyte
10	GO:0030850	prostate gland development	0.00340452	AR/GLI2/PTEN/WNT5A	ASDOocyte
10	GO:0060562	epithelial tube morphogenesis	0.003436099	ABL1/AR/CEP290/EPHA7/GLI2/LAMA1/LRP2/SCRIB/TBR1/TSC2/WNT5A	ASDOocyte
10	GO:0099054	presynapse assembly	0.003664341	EIF4G1/PTEN/SLITRK2/WNT5A	ASDOocyte
10	GO:0007626	locomotory behavior	0.003823962	ALK/CDH23/DPP4/DSCAM/LMX1A/PENK/PTEN/RELN	ASDOocyte
10	GO:0097061	dendritic spine organization	0.004288751	ADAM10/DOCK10/PTEN/RELN/SHANK3	ASDOocyte
10	GO:0035418	protein localization to synapse	0.004510734	ADAM10/RELN/SCRIB/STX3/WNT5A	ASDOocyte
10	GO:0099172	presynapse organization	0.004523814	EIF4G1/PTEN/SLITRK2/WNT5A	ASDOocyte
10	GO:0030198	extracellular matrix organization	0.004688046	ABL1/ADAM10/COL12A1/DPP4/HAS2/ITGA2/ITGA2B/ITGB3/LAMA1/LAMA3/MMP8/NTNG1	ASDOocyte
10	GO:0050772	positive regulation of axonogenesis	0.004740776	ADNP/DSCAM/L1CAM/MACF1/PLXNA4	ASDOocyte
10	GO:0043062	extracellular structure organization	0.004781724	ABL1/ADAM10/COL12A1/DPP4/HAS2/ITGA2/ITGA2B/ITGB3/LAMA1/LAMA3/MMP8/NTNG1	ASDOocyte
10	GO:0022612	gland morphogenesis	0.005009661	AR/GLI2/LAMA1/NFIB/SCRIB/WNT5A	ASDOocyte
10	GO:0050890	cognition	0.005312974	ADNP/GRIA1/JAKMIP1/LMX1A/PTEN/RELN/SHANK3/SLC6A1/SLC8A3/TBR1	ASDOocyte
10	GO:0050804	modulation of chemical synaptic transmission	0.005402148	ABL1/ADNP/GRIA1/IGSF9B/NTNG1/PTEN/RELN/SHANK3/SLC24A1/SLC6A1/SLC8A3/STX3/USP46	ASDOocyte
10	GO:0030100	regulation of endocytosis	0.005430487	ABL1/ITGA2/ITGB3/NCKAP1L/SCRIB/TSC2/USP46/WNT5A	ASDOocyte
10	GO:0099177	regulation of trans-synaptic signaling	0.005500061	ABL1/ADNP/GRIA1/IGSF9B/NTNG1/PTEN/RELN/SHANK3/SLC24A1/SLC6A1/SLC8A3/STX3/USP46	ASDOocyte
10	GO:0001704	formation of primary germ layer	0.005621527	COL12A1/ITGA2/ITGB3/LAMA3/MMP8/WNT5A	ASDOocyte
10	GO:0002685	regulation of leukocyte migration	0.00574192	ADAM10/CD200R1/DPP4/ITGA2/ITGA2B/NCKAP1L/SELP/WNT5A	ASDOocyte
10	GO:0097120	receptor localization to synapse	0.005867552	ADAM10/RELN/SCRIB/STX3	ASDOocyte
10	GO:0007254	JNK cascade	0.005902651	MAPK9/MMP8/NRK/RB1CC1/SASH1/SH3RF3/SPAG9/WNT5A	ASDOocyte
10	GO:0001843	neural tube closure	0.006017304	ABL1/LRP2/SCRIB/TSC2/WNT5A	ASDOocyte
10	GO:0032874	positive regulation of stress-activated MAPK cascade	0.006031225	MMP8/NRK/RB1CC1/SASH1/SH3RF3/SPAG9/WNT5A	ASDOocyte
10	GO:0007611	learning or memory	0.006063632	ADNP/GRIA1/LMX1A/PTEN/RELN/SHANK3/SLC6A1/SLC8A3/TBR1	ASDOocyte
10	GO:0030307	positive regulation of cell growth	0.006222713	ADAM10/ADNP/DSCAM/EIF4G1/L1CAM/MACF1/SLC9A1	ASDOocyte
10	GO:0060606	tube closure	0.006298946	ABL1/LRP2/SCRIB/TSC2/WNT5A	ASDOocyte
10	GO:0106027	neuron projection organization	0.006298946	ADAM10/DOCK10/PTEN/RELN/SHANK3	ASDOocyte
10	GO:0070304	positive regulation of stress-activated protein kinase signaling cascade	0.006418702	MMP8/NRK/RB1CC1/SASH1/SH3RF3/SPAG9/WNT5A	ASDOocyte
10	GO:1990138	neuron projection extension	0.006418702	ABL1/ADNP/DSCAM/L1CAM/MACF1/PLXNA4/WNT5A	ASDOocyte
10	GO:0099173	postsynapse organization	0.006619254	ADAM10/DOCK10/EPHA7/PTEN/RELN/SHANK3/WNT5A	ASDOocyte
10	GO:0048008	platelet-derived growth factor receptor signaling pathway	0.006629302	ABL1/BCAR1/PTEN/PTPRJ	ASDOocyte
10	GO:0051099	positive regulation of binding	0.006824434	ABL1/EBF2/EIF4G1/ITGA2/MMP8/TCF7L2/WNT5A	ASDOocyte
10	GO:0072175	epithelial tube formation	0.007006551	ABL1/CEP290/LRP2/SCRIB/TSC2/WNT5A	ASDOocyte
10	GO:0010631	epithelial cell migration	0.007057415	ABL1/BCAR1/DPP4/HAS2/ITGA2/ITGB3/MACF1/PRSS3/PTEN/SASH1/WNT5A	ASDOocyte
10	GO:0051668	localization within membrane	0.007199259	RELN/RILPL2/SCRIB/SEC24A/SHANK3	ASDOocyte
10	GO:0010634	positive regulation of epithelial cell migration	0.007248926	ABL1/BCAR1/HAS2/ITGA2/ITGB3/SASH1/WNT5A	ASDOocyte
10	GO:0090132	epithelium migration	0.007483053	ABL1/BCAR1/DPP4/HAS2/ITGA2/ITGB3/MACF1/PRSS3/PTEN/SASH1/WNT5A	ASDOocyte
10	GO:0140115	export across plasma membrane	0.007890325	ABCC4/KCNT2/SLC8A3/SLC9A1	ASDOocyte
10	GO:0045665	negative regulation of neuron differentiation	0.008109938	CERS2/EDNRB/EPHA7/LMX1A/PHOX2B/PTEN/RTN4RL1/WNT5A	ASDOocyte
10	GO:0014020	primary neural tube formation	0.00818575	ABL1/LRP2/SCRIB/TSC2/WNT5A	ASDOocyte
10	GO:0090130	tissue migration	0.008394292	ABL1/BCAR1/DPP4/HAS2/ITGA2/ITGB3/MACF1/PRSS3/PTEN/SASH1/WNT5A	ASDOocyte
10	GO:0001667	ameboidal-type cell migration	0.008586829	ABL1/BCAR1/DPP4/EDNRB/HAS2/ITGA2/ITGB3/MACF1/PHOX2B/PRSS3/PTEN/SASH1/WNT5A	ASDOocyte
10	GO:0046328	regulation of JNK cascade	0.009144619	MMP8/NRK/RB1CC1/SASH1/SH3RF3/SPAG9/WNT5A	ASDOocyte
10	GO:0010001	glial cell differentiation	0.009193076	ABL1/DICER1/NFIB/PHOX2B/PTEN/RELN/SLC8A3/TENM4	ASDOocyte
10	GO:0045165	cell fate commitment	0.009209625	AR/EBF2/GLI2/IL7/SLAMF6/TBR1/TCF7L2/TENM4/WNT5A	ASDOocyte
10	GO:0001708	cell fate specification	0.009262309	AR/GLI2/TBR1/TENM4/WNT5A	ASDOocyte
10	GO:0002687	positive regulation of leukocyte migration	0.009524969	ADAM10/ITGA2/ITGA2B/NCKAP1L/SELP/WNT5A	ASDOocyte

10	GO:0030879	mammary gland development	0.009524969	AR/GLI2/ITGA2/SCRIB/SERPINC1/WNT5A	ASDOocyte
11	GO:0042391	regulation of membrane potential	3.97E-07	ATP1A3/CACNA1D/CACNA1E/CACNA1H/CFTR/CTNNA3/GRIA1/GRIN2B/ IGSF9B/KCNMA1/MECP2/P2RX7/PIEZO1/PTPN3/RELN/SCN1A/SHANK2/SHANK3/ZMYND8	ASDEmbryo
11	GO:0106027	neuron projection organization	7.06E-07	ATP1A3/DOCK10/EPHB1/EPHB2/GRIN2B/RELN/SHANK2/SHANK3/ZMYND8	ASDEmbryo
11	GO:0097061	dendritic spine organization	3.64E-06	DOCK10/EPHB1/EPHB2/GRIN2B/RELN/SHANK2/SHANK3/ZMYND8	ASDEmbryo
11	GO:0031646	positive regulation of nervous system process	6.59E-06	DICER1/IGSF9B/ITGA2/RELN/SHANK2/SHANK3/TENM4	ASDEmbryo
11	GO:0021952	central nervous system projection neuron axonogenesis	7.88E-06	ADARB1/EPHB1/EPHB2/EPHB6/PLXNA4	ASDEmbryo
11	GO:0021545	cranial nerve development	1.83E-05	ADARB1/CTNNB1/EPHB1/EPHB2/NAV2/PLXNA4	ASDEmbryo
11	GO:0050808	synapse organization	2.18E-05	CNTN5/CTNNB1/DOCK10/DSCAM/EPHB1/EPHB2/GRIN2B/IGSF9B/ LAMB2/LRRTM3/MECP2/NFIA/RELN/SHANK2/SHANK3/ZMYND8	ASDEmbryo
11	GO:0050769	positive regulation of neurogenesis	2.36E-05	ALK/CDON/CNTN1/CTNNB1/DICER1/DSCAM/ECT2/EPHB2/PLXNA4/ PTPRZ1/RELN/SEMA5A/SHANK2/SHANK3/SIN3A/TENM4/ZMYND8	ASDEmbryo
11	GO:0021675	nerve development	2.41E-05	ADARB1/CTNNB1/DICER1/EPHB1/EPHB2/NAV2/PLXNA4	ASDEmbryo
11	GO:0060078	regulation of postsynaptic membrane potential	2.84E-05	GRIA1/GRIN2B/IGSF9B/MECP2/P2RX7/RELN/SHANK2/SHANK3/ZMYND8	ASDEmbryo
11	GO:0099565	chemical synaptic transmission, postsynaptic	3.07E-05	GRIN2B/IGSF9B/MECP2/P2RX7/RELN/SHANK2/SHANK3/ZMYND8	ASDEmbryo
11	GO:0021955	central nervous system neuron axonogenesis	4.14E-05	ADARB1/EPHB1/EPHB2/EPHB6/PLXNA4	ASDEmbryo
11	GO:0060291	long-term synaptic potentiation	4.52E-05	EPHB2/GRIN2B/MECP2/RELN/SHANK2/SHANK3/SLC24A1	ASDEmbryo
11	GO:0021953	central nervous system neuron differentiation	4.91E-05	ADARB1/ARX/CTNNB1/DYNC2H1/EPHB1/EPHB2/EPHB6/PLXNA4/SHANK3/SIN3A	ASDEmbryo
11	GO:0060997	dendritic spine morphogenesis	5.67E-05	DOCK10/EPHB1/EPHB2/RELN/SHANK2/SHANK3	ASDEmbryo
11	GO:0007411	axon guidance	6.76E-05	ARX/DSCAM/EPHB1/EPHB2/EPHB6/LAMB2/PLXNA4/RELN/ROBO3/SEMA5A/SHANK3/TRIO	ASDEmbryo
11	GO:1900449	regulation of glutamate receptor signaling pathway	6.79E-05	EPHB2/GRIA1/GRIN2B/RELN/SHANK2/SHANK3	ASDEmbryo
11	GO:0097485	neuron projection guidance	6.99E-05	ARX/DSCAM/EPHB1/EPHB2/EPHB6/LAMB2/PLXNA4/RELN/ROBO3/SEMA5A/SHANK3/TRIO	ASDEmbryo
11	GO:0050806	positive regulation of synaptic transmission	0.000113089	CACNA1D/EPHB2/GRIN2B/IGSF9B/MECP2/RELN/SHANK2/SHANK3/SLC24A1	ASDEmbryo
11	GO:0060996	dendritic spine development	0.000124627	DOCK10/EPHB1/EPHB2/RELN/SHANK2/SHANK3/ZMYND8	ASDEmbryo
11	GO:0060079	excitatory postsynaptic potential	0.0001325	GRIN2B/MECP2/P2RX7/RELN/SHANK2/SHANK3/ZMYND8	ASDEmbryo
11	GO:0007611	learning or memory	0.000134447	DNAH11/EPHB2/GRIA1/GRIN2B/MECP2/PIAS1/PRKAR1B/PTPRZ1/RELN/SHANK2/SHANK3	ASDEmbryo
11	GO:0048483	autonomic nervous system development	0.000152718	ADARB1/ARX/CTNNB1/NAV2/PLXNA4	ASDEmbryo
11	GO:0031644	regulation of nervous system process	0.000217814	DICER1/IGSF9B/ITGA2/RELN/SHANK2/SHANK3/TENM4/ZMYND8	ASDEmbryo
11	GO:0099601	regulation of neurotransmitter receptor activity	0.000219921	EPHB2/GRIA1/GRIN2B/RELN/SHANK2/SHANK3	ASDEmbryo
11	GO:0021602	cranial nerve morphogenesis	0.000259857	ADARB1/EPHB1/EPHB2/PLXNA4	ASDEmbryo
11	GO:0007409	axonogenesis	0.000264096	ADARB1/ARX/DSCAM/EPHB1/EPHB2/EPHB6/LAMB2/PLXNA4/ PTPRZ1/RELN/ROBO3/SEMA5A/SHANK3/SIN3A/TRIO	ASDEmbryo
11	GO:0021954	central nervous system neuron development	0.000269682	ADARB1/ARX/EPHB1/EPHB2/EPHB6/PLXNA4	ASDEmbryo
11	GO:0034765	regulation of ion transmembrane transport	0.000307764	CACNA1D/CACNA1E/CACNA1H/CFTR/DPP6/EPHB2/GRIA1/GRIN2B/ KCNMA1/P2RX7/PTPN3/RELN/SCN1A/SHANK2/SHANK3	ASDEmbryo
11	GO:0060292	long-term synaptic depression	0.000338458	GRIA1/SHANK2/SHANK3/SLC24A1	ASDEmbryo
11	GO:0097106	postsynaptic density organization	0.00038354	RELN/SHANK2/SHANK3/ZMYND8	ASDEmbryo
11	GO:0016358	dendrite development	0.000384128	ALK/DOCK10/DSCAM/EPHB1/EPHB2/MECP2/RELN/SHANK2/SHANK3/ZMYND8	ASDEmbryo
11	GO:0051899	membrane depolarization	0.000473551	CACNA1D/CACNA1E/CACNA1H/P2RX7/PTPN3/SCN1A	ASDEmbryo
11	GO:0050890	cognition	0.000484968	DNAH11/EPHB2/GRIA1/GRIN2B/MECP2/PIAS1/PRKAR1B/PTPRZ1/RELN/SHANK2/SHANK3	ASDEmbryo
11	GO:0099084	postsynaptic specialization organization	0.000486314	RELN/SHANK2/SHANK3/ZMYND8	ASDEmbryo
11	GO:2000310	regulation of NMDA receptor activity	0.000607314	EPHB2/GRIA1/GRIN2B/RELN	ASDEmbryo
11	GO:0099173	postsynapse organization	0.000620387	DOCK10/EPHB1/EPHB2/GRIN2B/RELN/SHANK2/SHANK3/ZMYND8	ASDEmbryo
11	GO:0009855	determination of bilateral symmetry	0.000661793	CTNNB1/DISP1/DNAH11/DYNC2H1/GATA4/IFT57/MICAL2	ASDEmbryo
11	GO:0086010	membrane depolarization during action potential	0.000675184	CACNA1D/CACNA1H/PTPN3/SCN1A	ASDEmbryo
11	GO:0009799	specification of symmetry	0.000691669	CTNNB1/DISP1/DNAH11/DYNC2H1/GATA4/IFT57/MICAL2	ASDEmbryo
11	GO:0007215	glutamate receptor signaling pathway	0.00082325	EPHB2/GRIA1/GRIN2B/RELN/SHANK2/SHANK3	ASDEmbryo
11	GO:0034205	amyloid-beta formation	0.000826739	APH1A/DYRK1A/GSAP/LRRTM3	ASDEmbryo
11	GO:0007416	synapse assembly	0.000927396	CNTN5/DSCAM/EPHB1/EPHB2/LRRTM3/MECP2/SHANK2/SHANK3	ASDEmbryo
11	GO:0045666	positive regulation of neuron differentiation	0.000956403	ALK/CDON/CNTN1/DSCAM/ECT2/PLXNA4/ RELN/SEMA5A/SHANK2/SHANK3/SIN3A/ZMYND8	ASDEmbryo
11	GO:0001941	postsynaptic membrane organization	0.001000825	EPHB2/RELN/SHANK3/ZMYND8	ASDEmbryo
11	GO:0048813	dendrite morphogenesis	0.00109622	DOCK10/DSCAM/EPHB1/EPHB2/RELN/SHANK2/SHANK3	ASDEmbryo
11	GO:0048588	developmental cell growth	0.00117514	CTNNB1/DSCAM/GATA4/GDF9/KMT2D/LAMB2/PLXNA4/SEMA5A/SIN3A	ASDEmbryo
11	GO:0048167	regulation of synaptic plasticity	0.001179162	EPHB2/GRIA1/GRIN2B/MECP2/RELN/SHANK2/SHANK3/SLC24A1	ASDEmbryo
11	GO:2001251	negative regulation of chromosome organization	0.001186447	ATRX/DNMT1/PHF8/SIN3A/SPDL1/TNKS/TRIP12	ASDEmbryo
11	GO:0030900	forebrain development	0.001220684	ALK/ARX/ATRX/CDON/CTNNB1/DYNC2H1/ EPHB2/PLXNA4/RELN/SEMA5A/SHANK3/SIN3A	ASDEmbryo
11	GO:0060999	positive regulation of dendritic spine development	0.001307992	RELN/SHANK2/SHANK3/ZMYND8	ASDEmbryo
11	GO:1900006	positive regulation of dendrite development	0.001344378	ALK/RELN/SHANK2/SHANK3/ZMYND8	ASDEmbryo
11	GO:0098815	modulation of excitatory postsynaptic potential	0.001423524	RELN/SHANK2/SHANK3/ZMYND8	ASDEmbryo
11	GO:0030048	actin filament-based movement	0.001437597	CACNA1D/CTNNA3/GATA4/MYO1H/MYO7B/NEB/SCN1A	ASDEmbryo
11	GO:0050804	modulation of chemical synaptic transmission	0.001441092	CACNA1D/EPHB1/EPHB2/GRIA1/GRIN2B/IGSF9B/ MECP2/PRKAR1B/RELN/SHANK2/SHANK3/SLC24A1/ZMYND8	ASDEmbryo
11	GO:0099177	regulation of trans-synaptic signaling	0.001469557	CACNA1D/EPHB1/EPHB2/GRIA1/GRIN2B/IGSF9B/MECP2/ PRKAR1B/RELN/SHANK2/SHANK3/SLC24A1/ZMYND8	ASDEmbryo
11	GO:0031641	regulation of myelination	0.001545985	CTNNB1/DICER1/PTPRZ1/TENM4	ASDEmbryo
11	GO:0008038	neuron recognition	0.002109407	DSCAM/EPHB2/ROBO3/SEMA5A	ASDEmbryo
11	GO:0042987	amyloid precursor protein catabolic process	0.002109407	APH1A/DYRK1A/GSAP/LRRTM3	ASDEmbryo
11	GO:0035176	social behavior	0.002269715	CHD8/MECP2/SHANK2/SHANK3	ASDEmbryo
11	GO:0050435	amyloid-beta metabolic process	0.002269715	APH1A/DYRK1A/GSAP/LRRTM3	ASDEmbryo
11	GO:0070509	calcium ion import	0.002341547	CACNA1D/CACNA1E/CACNA1H/CTNNB1/SLC24A1	ASDEmbryo
11	GO:0007368	determination of left/right symmetry	0.002459454	DISP1/DNAH11/DYNC2H1/GATA4/IFT57/MICAL2	ASDEmbryo
11	GO:0048593	camera-type eye morphogenesis	0.002459454	CDON/CTNNB1/DSCAM/EPHB1/EPHB2/LRP5	ASDEmbryo
11	GO:0032412	regulation of ion transmembrane transporter activity	0.002519524	CACNA1D/CFTR/EPHB2/GRIA1/GRIN2B/PTPN3/RELN/SHANK2/SHANK3	ASDEmbryo
11	GO:0048013	ephrin receptor signaling pathway	0.002592125	APH1A/EPHB1/EPHB2/EPHB6/GRIN2B	ASDEmbryo
11	GO:0051703	intraspecies interaction between organisms	0.002615137	CHD8/MECP2/SHANK2/SHANK3	ASDEmbryo
11	GO:0050954	sensory perception of mechanical stimulus	0.002881221	CACNA1D/CNTN5/ITGA2/MYO7B/NAV2/SCN1A/TECTA	ASDEmbryo
11	GO:0051056	regulation of small GTPase mediated signal transduction	0.002891532	ARHGAP26/ARHGEF3/CDON/ECT2/EPHB2/MADD/RELN/RHOBTB2/SRGAP3/TRIO	ASDEmbryo
11	GO:0034329	cell junction assembly	0.00290003	CNTN5/CTNNB1/DSCAM/ECT2/EPHB1/EPHB2/ ITGA2/LRRTM3/MECP2/SHANK2/SHANK3/TNS1	ASDEmbryo
11	GO:0010469	regulation of signaling receptor activity	0.002974982	EPHB2/GRIA1/GRIN2B/MUC4/RELN/SHANK2/SHANK3	ASDEmbryo
11	GO:0022898	regulation of transmembrane transporter activity	0.003300835	CACNA1D/CFTR/EPHB2/GRIA1/GRIN2B/PTPN3/RELN/SHANK2/SHANK3	ASDEmbryo
11	GO:0008361	regulation of cell size	0.003698372	DSCAM/KCNMA1/P2RX7/PLXNA4/SEMA5A/SHANK3/SIN3A	ASDEmbryo
11	GO:0031346	positive regulation of cell projection organization	0.003991515	ALK/CNTN1/DSCAM/ITGA2/P2RX7/PLXNA4/RELN/SEMA5A/SHANK2/SHANK3/ZMYND8	ASDEmbryo
11	GO:0010976	positive regulation of neuron projection development	0.004558306	ALK/CNTN1/DSCAM/PLXNA4/RELN/SEMA5A/SHANK2/SHANK3/ZMYND8	ASDEmbryo
11	GO:0032409	regulation of transporter activity	0.004660685	CACNA1D/CFTR/EPHB2/GRIA1/GRIN2B/PTPN3/RELN/SHANK2/SHANK3	ASDEmbryo
11	GO:0050803	regulation of synapse structure or activity	0.004826449	EPHB1/EPHB2/GRIN2B/LRRTM3/MX2/RELN/SHANK3/ZMYND8	ASDEmbryo
11	GO:0007062	sister chromatid cohesion	0.004890721	ATRX/CTNNB1/POGZ/TNKS	ASDEmbryo
11	GO:0006814	sodium ion transport	0.004946844	ATP1A3/CACNA1H/CNTN1/P2RX7/PTPN3/SCN1A/SIK1/SLC24A1	ASDEmbryo
11	GO:0007612	learning	0.005102521	EPHB2/MECP2/PIAS1/RELN/SHANK2/SHANK3	ASDEmbryo
11	GO:1905268	negative regulation of chromatin organization	0.005173177	DNMT1/PHF8/SIN3A/TRIP12	ASDEmbryo
11	GO:0035335	peptidyl-tyrosine dephosphorylation	0.005344076	DNAJC6/PTPN13/PTPN3/PTPRU/PTPRZ1	ASDEmbryo
11	GO:1902275	regulation of chromatin organization	0.005538392	ATRX/CTNNB1/DNMT1/MECP2/PHF8/SIN3A/TRIP12	ASDEmbryo
11	GO:0031056	regulation of histone modification	0.005814743	ATRX/CTNNB1/DNMT1/MECP2/SIN3A/TRIP12	ASDEmbryo
11	GO:0019233	sensory perception of pain	0.006027696	EPHB1/ITGA2/MECP2/P2RX7/SCN1A	ASDEmbryo
11	GO:0031060	regulation of histone methylation	0.006085591	ATRX/CTNNB1/DNMT1/MECP2	ASDEmbryo
11	GO:0051983	regulation of chromosome segregation	0.00626863	ATRX/CTNNB1/ECT2/SPDL1/TNKS	ASDEmbryo
11	GO:0032508	DNA duplex unwinding	0.006516252	ATRX/CHD2/CHD6/CHD8/NAV2	ASDEmbryo
11	GO:0034401	chromatin organization involved in regulation of transcription	0.007231865	CTNNB1/DNMT1/KMT2D/MECP2/PHF8/SIN3A	ASDEmbryo
11	GO:0048592	eye morphogenesis	0.007231865	CDON/CTNNB1/DSCAM/EPHB1/EPHB2/LRP5	ASDEmbryo
11	GO:0042982	amyloid precursor protein metabolic process	0.007460326	APH1A/DYRK1A/GSAP/LRRTM3	ASDEmbryo
11	GO:0050891	multicellular organismal water homeostasis	0.007460326	CFTR/HAS2/PRKAR1B/SFN	ASDEmbryo
11	GO:0021537	telencephalon development	0.007532983	ALK/ARX/CDON/CTNNB1/EPHB2/PLXNA4/RELN/SHANK3	ASDEmbryo
11	GO:0046822	regulation of nucleocytoplasmic transport	0.007575492	ECT2/MX2/SFN/TCF7L2/THOC2	ASDEmbryo
11	GO:0030004	cellular monovalent inorganic cation homeostasis	0.008147655	ATP1A3/CFTR/DMXL2/KCNMA1/TM9SF4	ASDEmbryo
11	GO:0006109	regulation of carbohydrate metabolic process	0.008193926	HAS2/NUP210/NUP93/P2RX7/PASK/PPP1R3C/SIK1	ASDEmbryo
11	GO:0051145	smooth muscle cell differentiation	0.008218445	CTNNB1/DNMT1/MECP2/PIAS1	ASDEmbryo
11	GO:0086003	cardiac muscle cell contraction	0.008615741	CACNA1D/CTNNA3/GATA4/SCN1A	ASDEmbryo
11	GO:0032392	DNA geometric change	0.008749148	ATRX/CHD2/CHD6/CHD8/NAV2	ASDEmbryo
11	GO:0034968	histone lysine methylation	0.008749148	ATRX/CTNNB1/DNMT1/KMT2D/MECP2	ASDEmbryo
11	GO:0090276	regulation of peptide hormone secretion	0.008821385	CACNA1D/CACNA1E/CFTR/LRP5/PASK/RPH3AL/TCF7L2	ASDEmbryo
11	GO:0046883	regulation of hormone secretion	0.00896404	CACNA1D/CACNA1E/CFTR/GDF9/LRP5/PASK/RPH3AL/TCF7L2	ASDEmbryo
11	GO:0002791	regulation of peptide secretion	0.009002783	CACNA1D/CACNA1E/CFTR/DISP1/LRP5/P2RX7/PASK/RPH3AL/SEC24A/TCF7L2	ASDEmbryo
11	GO:0045685	regulation of glial cell differentiation	0.009025395	CTNNB1/DICER1/PTPRZ1/TENM4	ASDEmbryo
11	GO:0050805	negative regulation of synaptic transmission	0.009025395	GRIA1/SHANK2/SHANK3/SLC24A1	ASDEmbryo
12	GO:0050808	synapse organization	3.93E-05	ABL1/ADAM10/DISC1/GJA10/MUSK/MYH10/NLGN2/ NLGN3/PCDHB13/PLXND1/PPFIA1/SLC6A1/SYNGAP1/WNT5A	ASDEmbryo
12	GO:0036465	synaptic vesicle recycling	5.47E-05	AP3D1/ITSN2/NLGN2/NLGN3/SCRIB/SNAP91	ASDEmbryo
12	GO:0007409	axonogenesis	0.00012315	ABL1/DISC1/GLI2/MARK2/MYH10/NFIB/NLGN3/ PLXND1/PRKCA/SPAST/SYNGAP1/TNN/TUBB3/WNT5A	ASDEmbryo
12	GO:0022612	gland morphogenesis	0.000132513	CAPN1/GLI2/NFIB/PLXND1/SCRIB/SLC9A3R1/WNT5A	ASDEmbryo
12	GO:0097479	synaptic vesicle localization	0.000160582	AP3D1/DTNBP1/NLGN2/NLGN3/SNAP91	ASDEmbryo
12	GO:0022604	regulation of cell morphogenesis	0.000176466	ABL1/ADAM10/DISC1/EPB41L3/ITSN2/KANK1/MARK2/MYH10/ NLGN3/PLEKHO1/PLXND1/SLC9A3R1/SYNGAP1/WNT5A	ASDEmbryo
12	GO:0048488	synaptic vesicle endocytosis	0.00023934	ITSN2/NLGN2/NLGN3/SCRIB/SNAP91	ASDEmbryo
12	GO:0140238	presynaptic endocytosis	0.00023934	ITSN2/NLGN2/NLGN3/SCRIB/SNAP91	ASDEmbryo

12	GO:0030010	establishment of cell polarity	0.000292798	KANK1/MARK2/MYO18A/NDC80/SCRIB/SLC9A3R1/WNT5A	ASDEmbryo
12	GO:0031532	actin cytoskeleton reorganization	0.000377645	ABL1/BCAR1/CDC42BPA/DTNBP1/INSRR/NRK	ASDEmbryo
12	GO:0046578	regulation of Ras protein signal transduction	0.00038344	ABL1/CYTH4/DENND4B/KANK1/LZTR1/MYO9B/STAMBP/SYNGAP1	ASDEmbryo
12	GO:0000281	mitotic cytokinesis	0.000481367	MYH10/SPAST/STAMBP/USP8/ZFYVE26	ASDEmbryo
12	GO:0045197	establishment or maintenance of epithelial cell apical/basal polarity	0.000652232	MARK2/SCRIB/SLC9A3R1/WNT5A	ASDEmbryo
12	GO:0043393	regulation of protein binding	0.000668077	ABL1/DISC1/DTNBP1/EP300/MARK2/PLXND1/WFIKKN2/WNT5A	ASDEmbryo
12	GO:0060443	mammary gland morphogenesis	0.000710877	CAPN1/GLI2/SCRIB/WNT5A	ASDEmbryo
12	GO:0035088	establishment or maintenance of apical/basal cell polarity	0.000909111	MARK2/SCRIB/SLC9A3R1/WNT5A	ASDEmbryo
12	GO:0061245	establishment or maintenance of bipolar cell polarity	0.000909111	MARK2/SCRIB/SLC9A3R1/WNT5A	ASDEmbryo
12	GO:0099173	postsynapse organization	0.000992133	ADAM10/MUSK/MYH10/NLGN2/NLGN3/SYNGAP1/WNT5A	ASDEmbryo
12	GO:0046580	negative regulation of Ras protein signal transduction	0.001230044	KANK1/LZTR1/STAMBP/SYNGAP1	ASDEmbryo
12	GO:0007416	synapse assembly	0.001417434	GJA10/MUSK/NLGN2/NLGN3/PCDHB13/PLXND1/WNT5A	ASDEmbryo
12	GO:0050803	regulation of synapse structure or activity	0.001528312	ABL1/ADAM10/DISC1/MUSK/NLGN2/NLGN3/SYNGAP1/WNT5A	ASDEmbryo
12	GO:0060563	neuroepithelial cell differentiation	0.001622886	ABL1/SCRIB/SLC9A3R1/TMC1	ASDEmbryo
12	GO:0009913	epidermal cell differentiation	0.00178903	CAPN1/FLNB/KRT85/PCSK6/PKP4/SCRIB/SLC9A3R1/TGM3/TMC1/WNT5A	ASDEmbryo
12	GO:0051058	negative regulation of small GTPase mediated signal transduction	0.001969404	KANK1/LZTR1/STAMBP/SYNGAP1	ASDEmbryo
12	GO:0034329	cell junction assembly	0.001973999	ABL1/EPB41L3/GJA10/MUSK/NLGN2/NLGN3/PCDHB13/PKP4/PLXND1/PRKCA/WNT5A	ASDEmbryo
12	GO:0010769	regulation of cell morphogenesis involved in differentiation	0.002040798	ABL1/ADAM10/DISC1/KANK1/MARK2/NLGN3/PLXND1/SYNGAP1/WNT5A	ASDEmbryo
12	GO:0061640	cytoskeleton-dependent cytokinesis	0.002116639	MYH10/SPAST/STAMBP/USP8/ZFYVE26	ASDEmbryo
12	GO:0099504	synaptic vesicle cycle	0.002212909	AP3D1/DTNBP1/ITSN2/NLGN2/NLGN3/SCRIB/SNAP91	ASDEmbryo
12	GO:0031032	actomyosin structure organization	0.002276139	ABL1/ARHGEF10L/CDC42BPA/EPB41L3/MYH10/MYO18A/PPFIA1	ASDEmbryo
12	GO:0002066	columnar/cuboidal epithelial cell development	0.002655368	SCRIB/SLC9A3R1/TMC1/WNT5A	ASDEmbryo
12	GO:0010977	negative regulation of neuron projection development	0.002745975	CERS2/ITM2C/KANK1/NLGN3/SYNGAP1/WNT5A	ASDEmbryo
12	GO:0008360	regulation of cell shape	0.002927542	EPB41L3/MARK2/MYH10/PLEKHO1/PLXND1/SLC9A3R1	ASDEmbryo
12	GO:0030902	hindbrain development	0.002927542	ABL1/GLI2/MYH10/NFIB/RERE/SCRIB	ASDEmbryo
12	GO:0022037	metencephalon development	0.003732518	ABL1/GLI2/MYH10/RERE/SCRIB	ASDEmbryo
12	GO:0007163	establishment or maintenance of cell polarity	0.003859312	KANK1/MARK2/MYO18A/NDC80/SCRIB/SLC9A3R1/WNT5A	ASDEmbryo
12	GO:0099003	vesicle-mediated transport in synapse	0.003859312	AP3D1/DTNBP1/ITSN2/NLGN2/NLGN3/SCRIB/SNAP91	ASDEmbryo
12	GO:0015800	acidic amino acid transport	0.00386795	PPFIA1/SLC38A7/SLC6A1/SLC9A3R1	ASDEmbryo
12	GO:0008544	epidermis development	0.004058137	CAPN1/FLNB/GLI2/KRT85/PCSK6/PKP4/SCRIB/SLC9A3R1/TGM3/TMC1/WNT5A	ASDEmbryo
12	GO:0018107	peptidyl-threonine phosphorylation	0.004169378	CAD/CDC42BPA/MARK2/PRKCA/WNT5A	ASDEmbryo
12	GO:0007265	Ras protein signal transduction	0.004225679	ABL1/CYTH4/DENND4B/KANK1/LZTR1/MYO9B/STAMBP/SYNGAP1/USP8	ASDEmbryo
12	GO:0051966	regulation of synaptic transmission, glutamatergic	0.004273197	DISC1/GRIK1/NLGN2/NLGN3	ASDEmbryo
12	GO:0000910	cytokinesis	0.004720577	MYH10/PKP4/SPAST/STAMBP/USP8/ZFYVE26	ASDEmbryo
12	GO:1990138	neuron projection extension	0.004720577	ABL1/DISC1/ITSN2/NLGN3/TNN/WNT5A	ASDEmbryo
12	GO:0050807	regulation of synapse organization	0.00479745	ABL1/ADAM10/DISC1/MUSK/NLGN2/NLGN3/WNT5A	ASDEmbryo
12	GO:0051648	vesicle localization	0.00479745	AP3D1/DTNBP1/NLGN2/NLGN3/PIK3CG/SCRIB/SNAP91	ASDEmbryo
12	GO:0002065	columnar/cuboidal epithelial cell differentiation	0.004807125	ABL1/SCRIB/SLC9A3R1/TMC1/WNT5A	ASDEmbryo
12	GO:0034765	regulation of ion transmembrane transport	0.00487878	ABL1/CACNA1A/CAPN1/CLCNKB/NLGN2/NLGN3/PIK3CG/SCN2A/SLC8A1/SLC9A3R1/TMC1	ASDEmbryo
12	GO:0015718	monocarboxylic acid transport	0.004991265	ABCC4/PNPLA8/SLC16A3/SLC38A7/SLC6A1/SLC9A3R1	ASDEmbryo
12	GO:0035725	sodium ion transmembrane transport	0.004991265	SCN2A/SCNN1D/SLC6A1/SLC8A1/SLC9A3R1/SLC9A5	ASDEmbryo
12	GO:0018210	peptidyl-threonine modification	0.005698142	CAD/CDC42BPA/MARK2/PRKCA/WNT5A	ASDEmbryo
12	GO:0051656	establishment of organelle localization	0.005899169	AP3D1/DTNBP1/MYH10/NDC80/PIK3CG/SCRIB/SLC9A3R1/SNAP91/SPAST/TMEM201	ASDEmbryo
12	GO:0051098	regulation of binding	0.006162546	ABL1/DISC1/DTNBP1/EP300/MARK2/MMP8/PLXND1/WFIKKN2/WNT5A	ASDEmbryo
12	GO:0006814	sodium ion transport	0.006304041	SCN2A/SCNN1D/SLC38A7/SLC6A1/SLC8A1/SLC9A3R1/SLC9A5	ASDEmbryo
12	GO:0021954	central nervous system neuron development	0.006451894	DISC1/GLI2/NFIB/WNT5A	ASDEmbryo
12	GO:0046328	regulation of JNK cascade	0.006518694	DTNBP1/MAPK8IP1/MMP8/NRK/SH3RF3/WNT5A	ASDEmbryo
12	GO:0050770	regulation of axonogenesis	0.006860855	ABL1/DISC1/MARK2/PLXND1/SYNGAP1/WNT5A	ASDEmbryo
12	GO:0016358	dendrite development	0.007177864	ADAM10/ARID1B/DISC1/DTNBP1/NLGN3/RERE/SYNGAP1	ASDEmbryo
12	GO:0031345	negative regulation of cell projection organization	0.007215863	CERS2/ITM2C/KANK1/NLGN3/SYNGAP1/WNT5A	ASDEmbryo
12	GO:0032092	positive regulation of protein binding	0.007315673	ABL1/EP300/PLXND1/WNT5A	ASDEmbryo
12	GO:0035418	protein localization to synapse	0.007315673	ADAM10/NLGN2/SCRIB/WNT5A	ASDEmbryo
12	GO:0072384	organelle transport along microtubule	0.007315673	AP3D1/DTNBP1/SPAST/TMEM201	ASDEmbryo
12	GO:0099111	microtubule-based transport	0.007583986	AP3D1/DNAH5/DTNBP1/SLC9A3R1/SPAST/TMEM201	ASDEmbryo
12	GO:0050804	modulation of chemical synaptic transmission	0.00824119	ABL1/CACNA1A/DISC1/DTNBP1/GRIK1/NLGN2/NLGN3/SLC6A1/SYNGAP1/USP8	ASDEmbryo
12	GO:0046330	positive regulation of JNK cascade	0.008300823	MAPK8IP1/MMP8/NRK/SH3RF3/WNT5A	ASDEmbryo
12	GO:0099177	regulation of trans-synaptic signaling	0.008362523	ABL1/CACNA1A/DISC1/DTNBP1/GRIK1/NLGN2/NLGN3/SLC6A1/SYNGAP1/USP8	ASDEmbryo
12	GO:0043506	regulation of JUN kinase activity	0.00858139	DTNBP1/MAPK8IP1/NRK/WNT5A	ASDEmbryo
12	GO:1903351	cellular response to dopamine	0.008918765	ABL1/DTNBP1/GNA11/SLC9A3R1	ASDEmbryo
12	GO:0048839	inner ear development	0.008979485	ADAM10/GLI2/SCRIB/SLC9A3R1/TMC1/WNT5A	ASDEmbryo
12	GO:0051056	regulation of small GTPase mediated signal transduction	0.00908729	ABL1/CYTH4/DENND4B/KANK1/LZTR1/MYO9B/STAMBP/SYNGAP1	ASDEmbryo
12	GO:0051899	membrane depolarization	0.009264689	ABL1/CACNA1A/SCN2A/SLC8A1	ASDEmbryo
12	GO:1903350	response to dopamine	0.009264689	ABL1/DTNBP1/GNA11/SLC9A3R1	ASDEmbryo
12	GO:0030879	mammary gland development	0.009325839	CAD/CAPN1/GLI2/SCRIB/WNT5A	ASDEmbryo
13	GO:0007409	axonogenesis	1.61E-05	ACTB/ADNP/CHL1/COL25A1/EPHA7/ITGB1/L1CAM/LAMA1/ LAMA3/LMX1A/MACF1/MAP1B/MYPN/PHOX2B/PTEN/TBR1	ASDEmbryo
13	GO:0006359	regulation of transcription by RNA polymerase III	6.16E-05	AR/BRF1/BRF2/ZNF345	ASDEmbryo
13	GO:0006383	transcription by RNA polymerase III	8.29E-05	AR/BRF1/BRF2/SNAPC5/ZNF345	ASDEmbryo
13	GO:0033555	multicellular organismal response to stress	8.72E-05	EDNRB/EIF4G1/NOS1/PENK/PTEN/USP46	ASDEmbryo
13	GO:0061912	selective autophagy	0.000288737	BAG3/BECN1/RB1CC1/SESN2/SPTLC2	ASDEmbryo
13	GO:0007160	cell-matrix adhesion	0.000374269	CASK/ITGA2B/ITGAL/ITGB1/L1CAM/MACF1/PTEN/PTPRJ/SLC9A1	ASDEmbryo

Additional file 2 Table S4

Cluster	Description	pvalue	gene
1	female sex differentiation	0.0023338	KMT2B/LHX8/MERTK/ZFPM2
2	spindle organization	0.0016627	CLASP2/EML1/RMDN1/TTK/TUBGCP6
3	meiotic cell cycle	0.0001853	ACTR3/CNTD1/DMRTC2/MEI1/TEX15/TTK/TUBGCP6
4	urogenital system development	0.0011143	JAG1/LGR4/NOTCH1/PCSK5/PROX1/TP63
5	meiosis I cell cycle process	0.0002307	CNTD1/DMRTC2/ESPL1/ING2/MEI1
6	genitalia development	0.0001499	BMP5/LGR4/ROR2/TP63
7	germ cell development	0.0076853	DMRTC2/ING2/KMT2B/MEI1/TSSK1B
8	in utero embryonic development	0.0002814	ACVR1/DSC3/FURIN/MIB1/MYH6/NOTCH1/PLG/SKIL/TRIM28/ZFPM2/ZMIZ1
9	reproductive structure development	0.0098392	CUL7/KMT2B/LGR4/NOTCH1/PLG/PSAPL1/TP63/TRIM28/ZFPM2
10	meiotic chromosome segregation	0.0001296	BUB1/MEI1/MLH3/TEX15/TTK
11	female sex differentiation	0.0003238	ADCYAP1R1/KMT2B/LHX8/MERTK/ROBO2/TP63/ZFPM2
12	development of primary female sexual characteristics	0.0057934	ADCYAP1R1/KMT2B/LHX8/ROBO2/ZFPM2

Additional file 2 Table S5

Gene	Description	Evidence
ABCE1	embryonic lethality at E7/delayed the resumption of meiosis (GVBD) and affected the extrusion of first polar body	MGI/PMID: 28380459
ACP3; ACPP	abnormal prostate gland dorsolateral lobe morphology/Human prostatic acid phosphatase (PAP, ACPP) and CD5 antigen-like (CD5L) were two proteins verified by ELISA to be differentially expressed between MII and GV oocytes	MGI/PMID: 35185790
ACTR3	Mice homozygous for a null allele die prior to E4.5 and exhibit abnormal embryogenesis/the asymmetric MII spindle position is dynamically maintained as a result of balanced forces governed by the Arp2/3 complex.	MGI/PMID: 21874009
ADCYAP1R1	female fertility	MGI
ADNP2	abnormal uterus morphology	MGI
AIM2	Elevated expression of IL-18 but not IL-1β gene is associated with NALP3 and AIM2 inflammasome in Polycystic Ovary Syndrome	PMID: 31935500
ARHGAP10	abnormal ovary morphology	MGI
BICC1	infertility; premature death	MGI
BMP5	ectopic ovary/Efficient studies of long-distance Bmp5 gene regulation using bacterial artificial chromosomes	MGI/PMID: 10677507
BUB1	embryonic lethality during organogenesis, complete penetrance/ Bub1 prevents chromosome misalignment and precocious anaphase during mouse oocyte meiosis	MGI/PMID: 16969117
C1QL2	female infertility	MGI
CANX	Calreticulin is required for development of the cumulus oocyte complex and female fertility	PMID: 26388295
CDHR5	abnormal female reproductive system morphology/Analysis of CDKN1C in fetal growth restriction and pregnancy loss	MGI/PMID: 31497289
CDK8	embryonic lethality before implantation, complete penetrance/Sequential analysis of global gene expression profiles in immature and in vitro matured bovine oocytes: potential molecular markers of oocyte maturation.	MGI/PMID: 21410957
CDKN1C	female infertility	MGI
CLASP2	PAR-1 and the microtubule-associated proteins CLASP2 and dynactin-p50 have specific localization on mouse meiotic and first mitotic spindles	PMID: 16123238
CNTD1	abnormal female meiosis; female infertility	MGI
CPEB3	oocyte degeneration; reduced female fertility/CPEB3 deficiency in mice affect ovarian follicle development and causes premature ovarian insufficiency	PMID: 34930897
CREBBP	Death in infancy; embryonic lethality during organogenesis, incomplete penetrance/ Distribution of co-activators CBP and p300 during mouse oocyte and embryo development	MGI/PMID: 16596650
DCAF1; VPRBP	embryonic lethality, complete penetrance/CRL4 complex regulates mammalian oocyte survival and reprogramming by activation of TET proteins.	MGI/PMID: 24357321
EEF2K	abnormal oocyte morphology; abnormal female reproductive system physiology	MGI
EIF2AK4	decreased litter size/GCN2 activation and eIF2alpha phosphorylation in the maturation of mouse oocytes	MGI/PMID: 18996085
EML1	preweaning lethality, incomplete penetrance/Echinoderm Microtubule Associated Protein Like 1 Is Indispensable for Oocyte Spindle Assembly and Meiotic Progression in Mice	MGI/PMID: 34124073
EMP2	decreased litter size; reduced fertility	MGI
EN2	reduced fertility	MGI
ESPL1	female infertility/Single-cell transcriptome analysis of human oocyte ageing	MGI/PMID: 34037315
EVC	postnatal lethality, complete penetrance; infertility	MGI
FAM20C	female infertility	MGI
FBN2	reduced fertility	MGI
FIGN	postnatal lethality, incomplete penetrance; reduced fertility; female infertility	MGI

FMR1	abnormal female germ cell morphology; abnormal ovary morphology	MGI
FURIN	abnormal oocyte morphology; decreased mature ovarian follicle number/ Oocyte-specific deletion of furin leads to female infertility by causing early secondary follicle arrest in mice	MGI/PMID: 28569793
GDF15	Presence of growth/differentiation factor-15 cytokine in human follicular fluid, granulosa cells, and oocytes	PMID: 29948426
GPATCH3	blind uterus	MGI
HHIP	neonatal lethality, complete penetrance/Dominant activation of the hedgehog signaling pathway alters development of the female reproductive tract/Hedgehog signaling regulates the basement membrane remodeling during folliculogenesis in the neonatal mouse ovary	MGI/PMID: 21809434/ PMID: 32542407
HTR2B	embryonic lethality during organogenesis, incomplete penetrance; reduced fertility/ Evolutionary transcriptomics implicates new genes and pathways in human pregnancy and adverse pregnancy outcomes./Human Cumulus Cells in Long-Term In Vitro Culture Reflect Differential Expression Profile of Genes Responsible for Planned Cell Death and Aging-A Study of New Molecular Markers	MGI/PMID: 34623259/ PMID: 32455542
HTT	Female sexual dysfunction	MGI
IL23A	The uterine immunological changes may be responsible for repeated implantation failure	PMID: 32120158
ITGB5	RUNX2, GPX3 and PTX3 gene expression profiling in cumulus cells are reflective oocyte/ embryo competence and potentially reliable predictors of embryo developmental competence in PCOS patients; Seven genes (RUNX2, PSAT1, ADAMTS9, CXCL1, CXCL2, CXCL3, and ITGB5) were targeted from our previous cDNA microarray data which isolated genes related to oocyte nuclear maturation in PCOS patients.	PMID: 24279306
JAG1	Activation of Notch Signaling by Oocytes and Jag1 in Mouse Ovarian Granulosa Cells.	PMID: 31609444
JAK3	premature death/Disrupted expression of long non-coding RNAs in the human oocyte: the possible epigenetic culprits leading to recurrent oocyte maturation arrest	MGI/PMID: 36018477
JMJD1C	Expression pattern of JMJD1C in oocytes and its impact on early embryonic development	PMID: 26782472
KLHL20	abnormal fertility/fecundity	MGI
KMT2B	female infertility/MLL2 is required in oocytes for bulk histone 3 lysine 4 trimethylation and transcriptional silencing	MGI/PMID: 20808952
LGR4	reduced female fertility	MGI
LHX8	Females show autophagy of oocytes associated with DNA damage, ovary fibrosis, and premature ovarian failure./ Heterozygous loss-of-function variants in LHX8 cause female infertility characterized by oocyte maturation arrest	MGI/PMID: 36029299
LRRK2	preweaning lethality, incomplete penetrance/ LRRK2 regulates actin assembly for spindle migration and mitochondrial function in mouse oocyte meiosis.	MGI/PMID: 34918122
LTBP1	lethality throughout fetal growth and development, incomplete penetrance/ Latent TGF-β binding protein-1 deficiency decreases female fertility.	MGI/PMID: 27956181
MAP2	Intracellular localization of MAP2-related protein (O-map) in prophase I and metaphase II oocytes of Xenopus.	PMID: 2031853
MCF2L2	Family-based association study of the MCF2L2 gene and polycystic ovary syndrome.	PMID: 19648752
MEI1	both sexes exhibit meiotic defects and are infertile; female infertility/ Novel biallelic mutations in MEI1: expanding the phenotypic spectrum to human embryonic arrest and recurrent implantation failure.	MGI/PMID: 34037756
MERTK	vagina atresia/Granulosa cells provide elimination of apoptotic oocytes through unconventional autophagy-assisted phagocytosis	MGI/PMID: 32531067
MLH3	abnormal female meiosis; female infertility/Meiotic arrest and aneuploidy in MLH3-deficient mice.	MGI/PMID: 12091911
MTCL1; SOGA2	female infertility	MGI
MTOR	embryonic lethality/Oocyte stage-specific effects of MTOR determine granulosa cell fate and oocyte quality in mice.	MGI/PMID: 29784807

MYT1	postnatal lethality, complete penetrance/A two-step inactivation mechanism of Myt1 ensures CDK1/cyclin B activation and meiosis I entry.	MGI/PMID: 20362450
NEK5	NEK5 regulates cell cycle progression during mouse oocyte maturation and preimplantation embryonic development.	PMID: 31304658
NLRP5	female infertility/Genetic factors as potential molecular markers of human oocyte and embryo quality.	MGI/PMID: 33895934
NME3	The NME gene family in zebrafish oogenesis and early development	PMID: 21394481
NPPB	Porcine natriuretic peptide type B (pNPPB) maintains mouse oocyte meiotic arrest via natriuretic peptide receptor 2 (NPR2) in cumulus cells.	PMID: 24615855
ORAI3	Downregulation of store-operated Ca2+ entry during mammalian meiosis is required for the egg-to-embryo transition	PMID: 23424198
PKD1L2	female infertility	MGI
PLG	reduced female fertility; Abnormality of the ovary	MGI
PRKCA	Disrupted expression of long non-coding RNAs in the human oocyte: the possible epigenetic culprits leading to recurrent oocyte maturation arrest.	PMID: 36018477
PTGFR	abnormal ovarian secretion	MGI
PTPRK	ZP3 is Required for Germinal Vesicle Breakdown in Mouse Oocyte Meiosis. We identified Protein tyrosine phosphatase, receptor type K (Ptprk), Aryl hydrocarbon receptor-interacting protein-like 1 (Aipl1), and Diaphanous related formin 2 (Diaph2) as potential candidates, and established a working model to explain how ZP3 affects GVBD	PMID: 28145526
REC8	abnormal female meiosis; female infertility	MGI
SENP1	lethality throughout fetal growth and development, complete penetrance/ Stromal Senp1 promotes mouse early folliculogenesis by regulating BMP4 expression.	MGI/PMID: 28770041
STK36	Female infertility	MGI
TMEFF2	lethality at weaning, complete penetrance/Global gene analysis of oocytes from early stages in human folliculogenesis shows high expression of novel genes in reproduction.	MGI/PMID: 22238370
TNS2; TENC1	reduced female fertility	MGI
TRIM28	Trim28 is required for epigenetic stability during mouse oocyte to embryo transition.	PMID: 22442485
TTK	reduced female fertility; abnormal female meiosis/ Mps1 kinase-dependent Sgo2 centromere localization mediates cohesion protection in mouse oocyte meiosis I.	MGI/PMID: 28947820
TULP4	abnormal uterus morphology	MGI
VCAN	embryonic lethality during organogenesis, complete penetrance/ Positive effects of amphiregulin on human oocyte maturation and its molecular drivers in patients with polycystic ovary syndrome.	MGI/PMID: 34741172
WDR48	decreased oocyte number; reduced female fertility	MGI
ZFPM2	Sex reversal; abnormal ovary morphology/GATA4 deficiency impairs ovarian function in adult mice.	MGI/PMID: 21248289
ZMIZ1	embryonic lethality during organogenesis, complete penetrance/ Molecular characterization of corona radiata cells from patients with diminished ovarian reserve using microarray and microfluidic-based gene expression profiling.	MGI/PMID: 22246450
ZNF84	A gene expression signature shared by human mature oocytes and embryonic stem cells	PMID: 19128516
ZP1	abnormal granulosa cell morphology; reduced female fertility	MGI
ZP2	female infertility/ZP2 pathogenic variants cause in vitro fertilization failure and female infertility.	MGI/PMID: 29895852

MGI: Mouse Genome Informatics (http://www.informatics.jax.org/); PMID: PubMed ID

Additional file 2 Table S6

ID	Age	Duration of infertility	IVF/ICSI Cycles	IVF/ICSI Time	Total Number of Oocytes Retrieved	MII	Fertilized Oocyte	Cleaved Embryos
Trio 1	32	7	1	2016-12	20	16	6	2
			2	2017-07	17	14	7	1
			3	2020-08	25	23	13	1
Trio 2	29	2	1	2015-08	13	13	10	2
			2	2015-11	21	19	14	2
Trio 3	38	15	1	2007-12	15	NA	7	2
			2	2011-07	18	NA	4	0
			3	2015-05	9	NA	3	0
			4	2015-07	3	3	1	0
			5	2016-12	9	8	5	0
			6	2017-03	7	5	5	1
			7	2017-09	3	2	2	0
			8	2019-03	4	NA	NA	0
Case 1	31	3	1	2015-02	NA	NA	NA	NA
			2	2015-04	NA	NA	NA	NA
			3	2015-12	1	1	1	0
Case 2	NA	NA	1	NA	Oocytes were arrested at GV			
			2	NA	Oocytes were arrested at GV			
Case 3	27	6	1	2020-10	21	8	8	0
Case 4	34	4	1	NA	She had undergone three cycles and 0 cleaved embryos were obtained			
			2	NA				
			3	NA				
Case 5	29	5	1	2021-05	11	11	10	1
			2	2021-06	13	11	11	0
Case 6	32	6	1	2018-11	20	5	4	0
			2	2019-04	28	15	8	0
			3	2019-07	24	16	14	0
Case 7	30	7	1	2016-03	8	7	0	0
			2	2016-05	12	7	7	0
Case 8	35	9	1	2017-05	3	3	2	2
			2	2017-09	9	7	6	2
			3	2018-05	6	4	4	0
Case 9	32	8	1	2012-05	6	NA	4	4
			2	NA	15	NA	12	1
			3	2016-01	12	NA	9	1
			4	2016-06	9	9	9	0
			5	2016-08	6	4	4	0
			6	2016-10	12	10	8	2
			7	2016-12	12	10	9	1

NA, not available.

Additional file 2 Table S7

ID	Position	cDNA change	Protein change	MT[a]	PP2[a]	gnomAD[b]	ACMGG classification (Evidence)	Phenotype	Inheri-tance
Trio 1	chr2:220116433	c.G229A	p.E77K	D	B	0	Pathogenic (PS2+PS3+PS4+PM2)	Embryonic arrest	*De novo*
Trio 2	chr2:220115564	c.T857C	p.L286P	D	P	0	Pathogenic (PS2+ PS3+PS4+PM2)	Embryonic arrest	*De novo*
Trio 3	chr2:220115381	c.G1040A	p.C347Y	D	D	0	Pathogenic (PS2+ PS3+PS4+PM2)	Embryonic arrest	*De novo*
Case 1	chr2:220116040	c.C381G	p.D127E	D	B	4.12E-06	Pathogenic (PS3+PS4+PM2)	Embryonic arrest	Unknown
Case 2	chr2:220115777	c.G644A	p.R215H	D	B	3.98E-06	Pathogenic (PS3+PS4+PM2)	Oocyte Maturation Arrest	Unknown
Case 3	chr2:220115736	c.C685T	p.R229C	D	D	0	Pathogenic (PS3+PS4+PM2)	Embryonic arrest	Unknown
Case 4	chr2:220115736	c.C685T	p.R229C	D	B	0	Pathogenic (PS3+PS4+PM2)	Embryonic arrest	Unknown
Case 5	chr2:220115603	c.C818T	p.A273V	D	D	0	Likely pathogenic (PS4+PM2+BS3)	Embryonic arrest	Unknown
Case 6	chr2:220115571	c.G850A	p.E284K	D	D	0	Pathogenic (PS4+PS3+PM2)	Embryonic arrest	Unknown
Case 7	chr2:220115514	c.G907C	p.V303L	D	B	0	Pathogenic (PS4+PS3+PM2)	Oocyte Maturation Arrest	Unknown
Case 8	chr2:220115480	c.C941T	p.A314V	D	D	0	Pathogenic (PS4+PS3+PM2)	Embryonic arrest	Unknown
Case 9	chr2:220115303	c.G1118A	p.R373H	D	D	3.98E-06	Pathogenic (PS4+PS3+PM2)	Embryonic arrest	Unknown

[a] Mutation assessment by MT (Mutation Taster) and PP2 (PolyPhen-2); B, benign; P, possibly damaging; D, damaging;

[b] gnomAD, Frequency of corresponding mutations in gnomAD database.

Additional file 2 Table S8

ID	cDNA Change	Name	Primer (5' to 3')	Application
Trio 1	c.G229A	*TUBA4A*_F1	GACTGAAAGGCAAGAACGAGA	Sanger sequencing
		*TUBA4A*_R1	AGCCCTGAAAATTCCTAGCCG	Sanger sequencing
Trio 2	c.T857C	*TUBA4A*_F2	TCATAGGAGTCGATGCCCACC	Sanger sequencing
		*TUBA4A*_R2	TCACAGCTTCTCTGCGCTTT	Sanger sequencing
Trio 3	c.G1040A	*TUBA4A*_F3	AGGGATAAGGGTCATGAACAGC	Sanger sequencing
		*TUBA4A*_R3	TGCAGAAAAGGCATACCACG	Sanger sequencing
Case 1	c.C381G	*TUBA4A*_F6	CGGCGGCAGATGTCATAGAT	Sanger sequencing
		*TUBA4A*_R6	GCCCCTCCCCTACCTAAACT	Sanger sequencing
Case 2	c.G644A	*TUBA4A*_F7	GGAAGTGGATGCGAGGGTAG	Sanger sequencing
		*TUBA4A*_R7	AGCCAGGTGATAGAGTCCCT	Sanger sequencing
Case 3	c.C685T	*TUBA4A*_F8	GGAAGTGGATGCGAGGGTAG	Sanger sequencing
		*TUBA4A*_R8	AGCCAGGTGATAGAGTCCCT	Sanger sequencing
Case 4	c.C685T	*TUBA4A*_F9	GGAAGTGGATGCGAGGGTAG	Sanger sequencing
		*TUBA4A*_R9	AGCCAGGTGATAGAGTCCCT	Sanger sequencing
Case 5	c.C818T	*TUBA4A*_F10	ATCCTTGGGCACCACATCTC	Sanger sequencing
		*TUBA4A*_R10	GTTCTTGCCTTTCAGTCTGACC	Sanger sequencing
Case 6	c.G850A	*TUBA4A*_F11	ACGCCCTCTTGGCATACATC	Sanger sequencing
		*TUBA4A*_R11	TTCTTGCCTTTCAGTCTGACC	Sanger sequencing
Case 7	c.G907C	*TUBA4A*_F12	CATCCTCGTCCTCATAGGAGTC	Sanger sequencing
		*TUBA4A*_R12	TACACCAACCTCAATCGCCT	Sanger sequencing
Case 8	c.C941T	*TUBA4A*_F13	GAGGGAACAAGAAACCGTGC	Sanger sequencing
		*TUBA4A*_R13	ATCACAGCTTCTCTGCGCTT	Sanger sequencing
Case 9	c.G1118A	*TUBA4A*_F14	CTGGGGTTATGCTTCCTGGG	Sanger sequencing
		*TUBA4A*_R14	TAAAGTGTGATCCCCGGCAC	Sanger sequencing
		TUBA4A-hCDS-F	ATGCGTGAATGCATCTCAGT	cloning human CDS
		TUBA4A-hCDS-R	AGGATGAGGGAGAAGAATAA	cloning human CDS
		TUBA4A-hRT-F	GGCAAGGAGATCATTGACCCAG	real-time PCR
		TUBA4A-hRT-R	CATCAGGAGTGAGGTGAAGCCA	real-time PCR
		ACTIN-hRT-F	CACCATTGGCAATGAGCGGTTC	real-time PCR
		ACTIN-hRT-R	AGGTCTTTGCGGATGTCCACGT	real-time PCR

Abbreviations: F forward, R reverse, CDS coding sequence
